# ZNF471 modulates EMT and functions as methylation regulated tumor suppressor with diagnostic and prognostic significance in cervical cancer

**DOI:** 10.1007/s10565-021-09582-4

**Published:** 2021-02-10

**Authors:** Samatha Bhat, Shama Prasada Kabekkodu, Divya Adiga, Rayzel Fernandes, Vaibhav Shukla, Poonam Bhandari, Deeksha Pandey, Krishna Sharan, Kapaettu Satyamoorthy

**Affiliations:** 1 Department of Cell and Molecular Biology, Manipal School of Life Sciences, Manipal Academy of Higher Education, Manipal, Karnataka 576104 India; 2 Department of Obstetrics & Gynaecology, Kasturba Medical College, Manipal Academy of Higher Education, Manipal, Karnataka 576104 India; 3 Department of Radiotherapy and Oncology, Kasturba Medical College, Manipal Academy of Higher Education, Manipal, Karnataka 576104 India

**Keywords:** Promoter methylation, ZNF471, EMT, Co-methylation, Cervical cancer

## Abstract

Cervical cancer (CC) is a leading cause of cancer-related death among women in developing countries. However, the underlying mechanisms and molecular targets for therapy remain to be fully understood. We investigated the epigenetic regulation, biological functions, and clinical utility of zinc-finger protein 471 (ZNF471) in CC. Analysis of cervical tissues and five independent public datasets of CC showed significant hypermethylation of the *ZNF471* gene promoter. In CC cell lines, promoter DNA methylation was inversely correlated with *ZNF471* expression. The sensitivity and specificity of the *ZNF471* hypermethylation for squamous intraepithelial lesion (SIL) vs tumor and normal vs tumor was above 85% with AUC of 0.937. High methylation and low *ZNF471* expression predicted poor overall and recurrence-free survival. We identified −686 to +114 bp as *ZNF471* promoter, regulated by methylation using transient transfection and luciferase assays. The promoter CpG site methylation of *ZNF471* was significantly different among cancer types and tumor grades. Gal4-based heterologous luciferase reporter gene assays revealed that *ZNF471* acts as a transcriptional repressor. The retroviral mediated overexpression of *ZNF471* in SiHa and CaSki cells inhibited growth, proliferation, cell migration, invasion; delayed cell cycle progression in vitro by increasing cell doubling time; and reduced tumor growth in vivo in nude mice. ZNF471 overexpression inhibited key members of epithelial-mesenchymal transition (EMT), Wnt, and PI3K-AKT signaling pathways. ZNF471 inhibited EMT by directly targeting vimentin as analyzed by bioinformatic analysis, ChIP-PCR, and western blotting. Thus, *ZNF471* CpG specific promoter methylation may determine the prognosis of CC and could function as a potential tumor suppressor by targeting EMT signaling.

**Supplementary Information:**

The online version contains supplementary material available at 10.1007/s10565-021-09582-4.

## Introduction

Cervical cancer (CC) is the most common malignancy affecting women in developing countries and accounted for 5,70,000 cases and more than 3,11,000 deaths in 2018. CC mortality is declining due to early detection through exfoliative cytology and the development of novel therapeutic strategies (Wheeler 2007). However, in India and China, both the incidence and mortality of CC are on the rise (Giftson Senapathy et al. 2011). This is due to disease diagnosis at advanced stages and lack of coherent follow-up leading to poor response to treatment. The molecular targets and underlying mechanisms leading to CC remain elusive and require detailed investigation. Multi-step cervical carcinogenesis is associated with HPV infection coupled with genetic and epigenetic changes. In contrast to genetic changes, epigenetic modifications are generally reversible, making it amenable for potential therapeutic interventions. Thus, elucidation of DNA methylation regulated genes may help in identifying targets for clinical application and may decipher the molecular mechanisms leading to the pathogenesis of CC (Bhat et al. 2017b; Bhat et al. 2016; Kabekkodu et al. 2014; Li et al. 2016). Zinc finger proteins (ZNFs) play a critical role in diverse biological functions such as cell proliferation, differentiation, apoptosis, and carcinogenesis (Lupo et al. 2013). For example, *ZNF545*, *ZNF569*, *ZNF383*, and *ZNF331* are reported to act as tumor suppressor genes, while *ZNF147*, *ZNF217*, *ZNF278*, *GLI1*, and *Evi9* promote tumor progression (Huang et al. 2006; Wang et al. 2013; Xiao et al. 2014; Yu et al. 2013). We have reported the hypermethylation of multiple gene promoters in CC (Bhat et al. 2017b). Hypermethylation of *ZNF471* is reported in squamous cell carcinoma of the tongue, colorectal cancer, oropharyngeal squamous cell carcinoma, esophageal cancer, breast cancer, and aging (Bhat et al. 2017a; Lleras et al. 2011; Marttila et al. 2015; Mitchell et al. 2014; Sun et al. 2020; Tao et al. 2020). However, the cause and consequences of *ZNF471* hypermethylation in CC are yet to be established. Here, we have evaluated the gene expression, epigenetic regulation, biological functions, and clinical relevance of *ZNF471* in CC. We show that *ZNF471* acts as a potential tumor suppressor by regulating epithelial-mesenchymal transition (EMT), and methylation of specific CpG sites occurs within *ZNF471* promoter in different tumors and their grades.

## Materials and methods

### Cell lines

Three CC cell lines (SiHa, HeLa and CaSki), seven non-CC cell lines (SCC4, Jurkat, CAL27, HT29, HCT15, MCF7, and MDAMB231) and Phoenix cells were purchased from ATCC, USA, and sub-cultured according to the protocol available at www.atcc.org/. The human diploid dermal fibroblast cell line was established at Manipal School of Life Sciences, MAHE, and Manipal. SiHa and CaSki cell lines were maintained in DMEM and RPMI media supplemented with 10% fetal bovine serum (FBS). Tumor cell lines were authenticated for its origin through STR analysis (Supplementary File 1).

### Patient samples and public datasets

Normal (*n*=20), the squamous intraepithelial lesion (SIL, *n*=20), and malignant tissues (SCC, *n*=20) were obtained from participants visiting Kasturba Medical College (KMC), Manipal, India, with the prior approval from Institutional Ethical Committee of Kasturba Hospital, MAHE. Exfoliative cells were collected from normal and participants with SIL who visited KMC for regular cervical screening, while primary tumors were collected from freshly diagnosed cases who have not undergone any chemotherapy and/or radiotherapy and exhibited no other diagnosed cancers using the punch biopsy method. The tumor tissues consisted of more than 80% of tumor cells as reviewed by pathologists and classified according to Bethesda System. The median age was 46, 53, and 56.5 years, respectively, for normal, SIL, and SCC participants (Supplementary Table 1). A cross-validation study was performed using independent public databases consisting of 608 samples (normal (*n*=208), SIL [LSIL+HSIL (*n*=48)], SCC (*n*=349)] and 3 CC cell lines) (Supplementary Table 2). The clinicopathological characteristics of public data are shown in Supplementary Table 3 (Burk et al. 2017; Farkas et al. 2013; Teschendorff et al. 2012; Zhuang et al. 2012).

### Quantitative DNA methylation analysis

DNA was extracted from clinical samples and cell lines by PureLink Genomic DNA Mini Kit (ThermoFisher Scientific, USA). DNA methylation analysis at single-base resolution was performed by bisulfite-based Sanger sequencing as published earlier (Bhat et al. 2017a; Kabekkodu et al. 2014). The primers used in the study are listed in Supplementary Table 4.

### Demethylation experiments and RT-PCR

Cells (3×10^5^) were seeded in 10 cm culture dishes and treated with 5-aza-2-deoxy cytidine at concentrations of 5 μM, 10 μM, and 20 μM (Calbiochem, USA) for 3 days. RNA was extracted using TRIZOL® Reagent (Invitrogen, USA), and cDNA was synthesized using High-Capacity cDNA Archive Kit (Thermofisher Scientific, USA) and used for gene expression analysis by semiquantitative RT-PCR (Supplementary Table 4). Bisulfite-based Sanger sequencing was performed using DNA isolated from SiHa and CaSki before and after treatment with 5 μM of 5-aza-2-deoxy cytidine as described above.

### Promoter characterization

The region spanning −686 to +114 bp with respect to the transcription start site (TSS) of *ZNF471* was cloned into *Kpn*I and *Hind*III sites of the pGL3-Basic vector to generate pGL3-ZNF471 (−686 to +114 bp)-luc (Promega, USA). *SssI* methylated promoter constructs were also used in the experiment (Supplementary Table 4). Six deletion constructs of pGL3-ZNF471 (−686 to +114 bp)-luc, namely, pGL3-ZNF471 (−526 to +118 bp)-luc, pGL3-ZNF471 (−305 to +118 bp)-luc, pGL3-ZNF471 (−201 to +118 bp)-luc, pGL3-ZNF471 (del −509 to −305 bp)-luc, pGL3-ZNF471 (del −301 to −201 bp)-luc, pGL3-ZNF471 (del −161 to +79 bp)-luc, were prepared by restriction digestion approach and verified by DNA sequencing. Promoter activity after 48 h of transfection was measured using the dual luciferase assay kit (Promega, USA) (Kabekkodu et al. 2014).

### Construction of ZNF471 retroviral expression vector and generation of stable clones

The human cDNA encoding complete *ZNF471* (Open Biosystems, USA) was cloned into *Bam*HI and *Xho*I restriction sites of retroviral expression vector pMXs-IRES-GFP (Cell Biolabs, USA) to generate pMXs-ZNF471-IRES-GFP. The retroviral mediated transduction of SiHa and CaSki cells and isolation of stable cells expressing *ZNF471* were performed as described previously (Kabekkodu et al. 2014). Upon transduction, cells expressing GFP were isolated by cell sorting using FACS Calibur (Becton-Dickinson, USA), confirmed by RT-PCR and western blot analysis.

### Western blot analysis

Cells were lysed in RIPA buffer, proteins were separated on a 10% SDS-polyacrylamide gel by electrophoresis and transferred onto Nitran membrane (Sigma-Aldrich, USA). These were blocked with 5% non-fat dry milk and incubated with primary antibody (ZNF471 (1:3000; Sigma, USA), p-AKT (Ser473), total AKT, anti-CTNNB1, non-phospho-CTNNB1, CDH1, p-ELK-1(Ser383), total ELK-1, SNAI2, CCND1, c-MYC, DVL2, WNT3A, p-LRP6, total LRP6, β-actin (1:3000, Cell Signaling Technologies, USA), RAF1, (1:3000, Santa Cruz Technologies, USA) and SNAII, CDH2, VIM (1:3000, Cloud Clone, USA), Ki67, and Caspase 3 (1:3000, Novus Biological, USA)) overnight at 4°C and subsequently by secondary antibodies either anti-mouse IgG-HRP or anti-rabbit IgG-HRP (1:5000, Cell Signaling, USA). Proteins were visualized by SuperSignal™ West Pico Chemiluminescent Substrate (Thermo Scientific, USA), and imaging was performed with Image Quant LAS 4000 (GE Healthcare, USA).

### Heterologous Gal4 reporter assay

The human cDNA encoding complete ZNF471 (Open Biosystems, USA) was cloned into the *Sma*I site of pSG424 (Addgene, USA) to obtain pSG424-ZNF471. SiHa and HepG2 cells were transfected with 0.4 μg of pSG424, pSG424-ZNF471 and 1 μg of pGL2-GAL4-UAS-Luc (Addgene, USA) using Lipofectamine LTX (Life Technologies, USA) in 6-well plates. Luciferase assay was performed 48 h post-transfection using the dual luciferase assay kit (Promega, USA). The data were normalized as published previously (Agarwal et al. 1999).

### Chromatin immunoprecipitation (ChIP) PCR

SiHa cells ectopically expressing ZNF471 were cross-linked with formaldehyde, and chromatin was isolated and sheared by CD-200. Bioruptor® Sonication System (Diagenode, USA) to obtain an average fragment size of 200–500 bp. Sheared chromatin was precipitated with anti-ZNF471 antibody (HPA066695; Sigma-Aldrich, USA). Equal concentration of non-specific IgG (Jackson Laboratory, USA) was used as control. The immunoprecipitated protein-DNA complex was captured using protein A/G immunomagnetic beads (Sino Biological Inc, China), and DNA was isolated by standard phenol chloroform and ethanol precipitation method. PCR was performed to identify the enriched genes (Supplementary Table 4).

### Anchorage-dependent colony formation assay

In brief, 500 cells were plated in duplicates in a 6-cm petri plate. After 12 days, the medium was removed; cells were washed with PBS and stained with 0.5% crystal violet in methanol for 10–15 min. Excessive stains were removed by washing with distilled water, plates were photographed, and colonies were counted (Hu et al. 2013; Kabekkodu et al. 2014).

### Anchorage-independent colony formation assay

In 6-well plates, 1×10^3^ SiHa cells/well were mixed with 2 mL of 0.3% Nobel agar (DMEM+ 10% FBS) and was overlaid on top of 3 mL of 0.6% bottom agar base (DMEM+ 20% FBS), 0.5 mL of complete medium was added every week, and at the end of 4 weeks, colonies were counted (Kabekkodu et al. 2014; Kaneda et al. 2004).

### Cell doubling and growth curve analysis

About 2×10^4^ cells were plated in 35 mm petri dishes for a 5-day growth curve analysis. The cells were trypsinized at each time point and counted using a hemocytometer. The cell doubling time was calculated using a cell doubling time calculator (http://www.doubling-time.com/compute.php).

### Actin-phalloidin staining and confocal microscopy

The transfected cells were cultured on a coverslip, fixed with 4% paraformaldehyde for 10 min, and stained with 15 μg of Alexa Fluor phalloidin (Sigma, USA) for 30 min. The cells were washed with PBS, stained with Hoechst stain (1μg/mL) for 5 min, washed briefly with PBS, and mounted onto a glass slide using VECTASHIELD (Vector Laboratories, USA). The images were captured and analyzed using a Leica SP8 confocal microscope and Leica application suite (Leica Microsystems, Germany).

### Cell cycle analysis

Cells were synchronized by serum starvation for 24 h, followed by culturing the cells with complete medium for 24 h. Cells were collected by trypsinization, fixed in 70% ethanol for overnight at 4°C, treated with RNase-A (10 ug/mL; Sigma-Aldrich, USA), and stained with propidium iodide (50 μg/mL; Sigma-Aldrich, USA) for 30 min at room temperature in the dark and analyzed in a FACS Calibur flow cytometer and Cell Quest software (BD Biosciences, USA).

### Annexin-V staining

Cells were treated with 1 μM doxorubicin for 1 h, washed with PBS, and cultured in the presence of complete medium for 24 h. The apoptosis rate was quantified by flow cytometry after staining with Annexin-V and PI (BD Biosciences, USA) (khaki-khatibi et al. 2019).

### Cell migration and invasion assay

Transfected cells were grown to confluence in 6-well plates and starved for 24 h, and a scratch was made using the sterile microtip. Subsequently, a fresh medium with 10% FBS was added, and the migration of cells into the wound area was monitored until the wounds are closed. The migration rate/index was calculated as published previously (Xu et al. 2012). In brief, 1×10^5^ transfected cells were seeded on wells containing agarose spots (fibronectin (20 μg/mL) or type I collagen (100 μg/mL)) in serum-free DMEM medium and incubated at 37°C for 24–48 h. The movement of the cell onto the agarose spot was captured using the Rolera emc2 camera and CK-41 microscope (Olympus, Japan) and analyzed by Image-Pro Plus software (Media Cybernetics, USA) (Wiggins and Rappoport 2010).

### Induction of epithelial to mesenchymal transition (EMT)

Transfected cells were treated with FGF-1 (5 ng/mL), TGF-β (2 ng/mL), IL-6 (25 ng/mL), EGF-1 (50 ng/mL), and TNF-α (10 ng/mL) for 72 h. The images were captured as described above. Semi-quantitative RT-PCR was performed for genes such as *VIM*, *CTNNB1*, *CDH1*, *CDH2*, *TW1*, *TW2*, *SNAI1*, *SNAI2*, and *ZEB1* with β-actin as an internal control. The primer sequence, annealing temperature, and amplicon size are listed in Supplementary Table 4. The NIH Image J software (http://imagej.nih.gov/ij/) was used to analyze the relative gene expression.

### In vivo tumorigenicity assay, hematoxylin and eosin (H&E), and Masson’s trichrome staining

The animal experiments were performed with prior approval from the Institutional Animal Ethics Committee (IAEC) of Manipal Academy of Higher Education, Manipal, Karnataka, India. Female athymic BALB/c nude mice (4–5 weeks old, 16–19 g weight) were maintained under a controlled specific pathogen-free (SPF) environment, with 20–25°C temperature, 40–60% humidity, and 12-h light or dark cycle. Animals were handled with aseptic procedures, under laminar air flow in a SPF facility. The experimental animals were sheltered in a cage (*n* = 3 per cage) and maintained on an ad libitum diet and autoclaved water. Control and ZNF471 expressing SiHa and CaSki cells (1×10^7^ cells/mL) were mixed with Matrigel and injected (*n* = 5 per group) subcutaneously into the lower flank. Isoflurane (4%) was used as an anesthetic agent during inoculation. Dimensions of the palpable tumor were analyzed externally after 10 days of injection, with digital calipers. The tumor volume was calculated with the equation *V* = *ab*^2^/2 (*a*, length; b, width). Animals were sacrificed using ketamine (90 mg/kg) followed by cervical dislocation, and tumor tissues were removed. Images were taken immediately after excision. Tumor cryosections were used for H&E and Masson’s trichrome staining and evaluated by pathologists.

### Bioinformatic and statistical analysis

Student’s *t*-test, ANOVA, and Fisher’s exact test were performed using Microsoft Excel (Microsoft Office, USA) and GraphPad Prism free online version (GraphPad Software, USA). PCA, scatter plot matrix, and co-methylation analysis was performed using the comet R program (Bailey et al. 2009). The sensitivity and specificity analyses were executed using MedCalc (http://www.medcalc.org/ calc/diagnostic_test.php) and randomForest and ROCR using R package, respectively. In brief, samples with a β-value of 0.2 and above were considered as methylated and are being used as a cutoff value for sensitivity and specificity analysis. For random forest (RF) analysis, the methylation level per sample was calculated by taking the average methylation across the CpG sites. Samples were randomly selected as training and validation sets by the software. Using the tree building algorithm (bootstrap sampling method), 1000 random trees were built, and the misclassification error (OOB error) was calculated by the confusion matrix. The area under the curve (AUC) were determined using RandomForest and ROCR packages.

Kaplan–Meier survival (overall survival (OS) and recurrence-free survival (RFS)) was performed for TCGA-CESC methylation and expression data using GraphPad Prism 5 free online version and KM-plotter (https://kmplot.com/analysis/index.php?p=service), respectively. Log-rank (Mantel-Cox) and Gehan-Breslow-Wilcoxon tests were used to calculate the hazard ratio. *P*-value ≤ 0.05 was considered as statistically significant.

## Results

### ZNF471 is hypermethylated in SIL and SCC

**Fig. 1:**
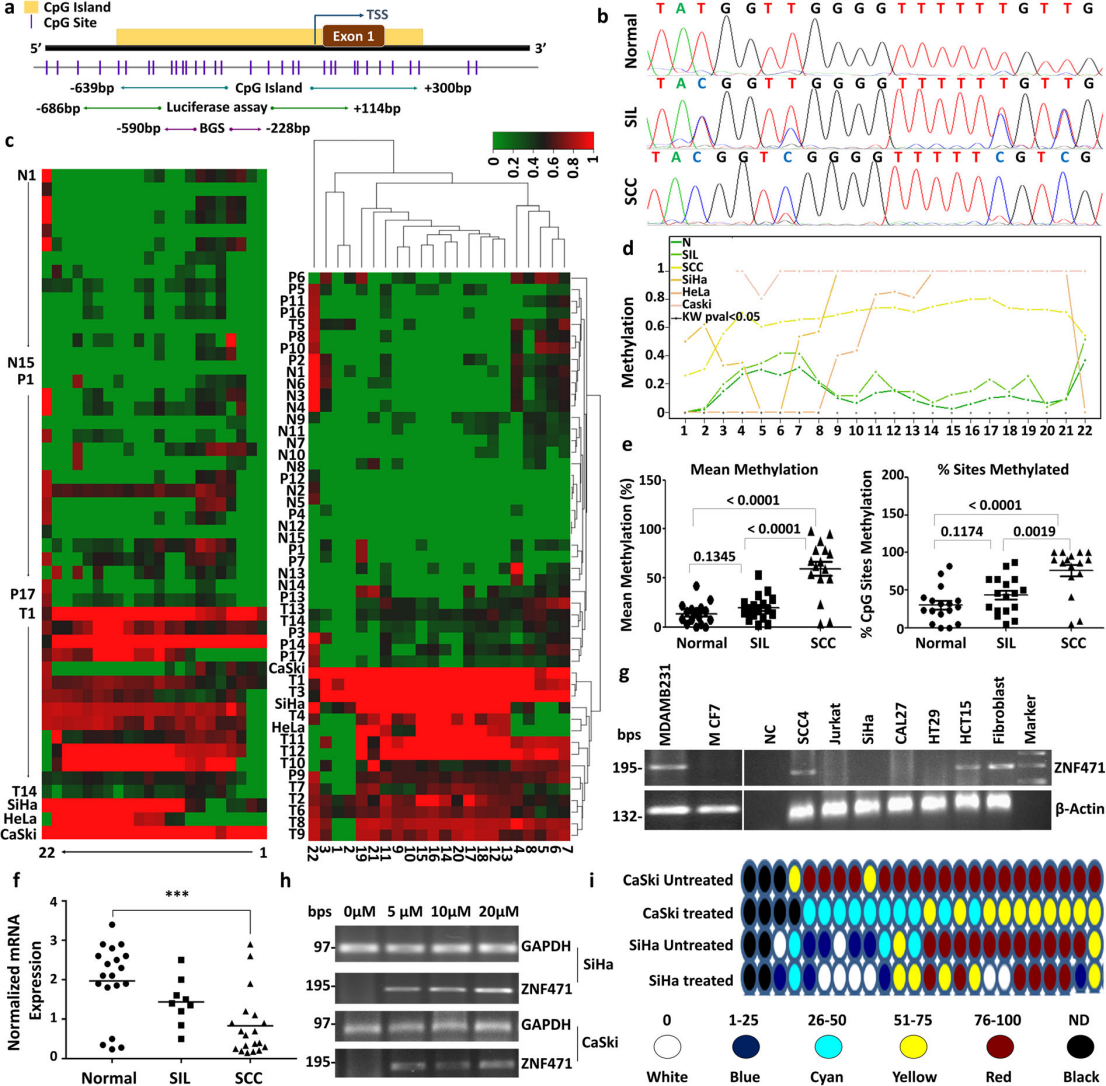
DNA methylation analysis of the *ZNF471* promoter. *ZNF471* expression is downregulated by promoter methylation in primary CC tissue samples, cell lines. **a** Genomic organization of *ZNF471* representing the region used for methylation analysis and luciferase assay. **b** The electropherogram of unmethylated and methylated *ZNF471* promoter in normal SIL and SCC tissues, respectively. **c** Hierarchical clustering of 22 CpG sites based on methylation level in normal, SIL, SCC, and 3 CC cell lines (SiHa, HeLa and CaSki) by Euclidean distance and average linkage method using Heatmapper tool (http://www.heatmapper.ca/). The DNA methylation level showed substantial variability of hypermethylation in general and at specific CpG sites in SIL, SCC, and CC cell lines as opposed to normal samples. **d** The overall methylation profile of individual CpG sites in normal, SIL, SCC samples, and CC cell lines. The mean methylations of normal, SIL, and SCC tissues were 13.23 ± 2.587, 19.46 ± 3.127, and 59.36 ± 7.154, respectively. The Kruskal–Wallis test *P*-value < 0.05 as analyzed by the Methylation plotter tool (http://gattaca.imppc.org:3838/methylation plotter/). **e** The dot plot representation of *ZNF471* promoter methylation in individual samples in our study. *ZNF471* methylation was significantly different between normal and SCC and SIL and SCC samples, respectively (*P* < 0.0001). **f** *ZNF471* expression in normal (*n*=20), SIL(*n*=10), and SCC (*n*=20). As per one-way ANOVA analysis *ZNF471* expression was significantly downregulated in SIL and SCC when compared with normal samples (*P* < 0.0001). **g** *ZNF471* expression was analyzed in 1 cervical cancer (SiHa), 7 non-cervical cancer (MDAMB231, MCF7, SCC4, Jurkat, CAL27, HT29, and HCT15), and a diploid fibroblast cell lines by semi-quantitative RT-PCR with β-actin as an internal control. SiHa, MCF7, Jurkat, Cal27, and HT29 showed the absence of *ZNF471* expression. MDAMB231, SCC4, HCT15, and normal diploid fibroblast showed *ZNF471* expression. **h** *ZNF471* expression was silenced in SiHa and CaSki cell lines. Both SiHa and CaSki cell lines showed reactivation upon treatment with 5 aza-2-deoxy cytidine (5 μM, 10 μM, and 20 μM) for 3 days. GAPDH was used as an internal control. **i** The treatment with 5 μM of 5-aza-2-deoxy cytidine reduced the DNA methylation level of multiple CpG sites throughout the *ZNF471* promoter in SiHa and CaSki cells

We have analyzed DNA methylation and gene expression of *ZNF471* in our samples and cohort of samples from 5 independent datasets (E-GEOD-30760, E-GEOD-30759, E-GEOD-41384, E-GEOD-46306, and TCGA) (Supplementary Tables 2 and 3). In our samples, DNA methylation (promoter and individual CpGs) levels between −590 to −228 were significantly higher in tumor tissues when compared with normal and SIL samples (*P*<0.05; Fig. 1a–d). Analysis of the public datasets revealed that the ZNF471 gene promoter is significantly hypermethylated in SIL and tumor tissues when compared with normal samples (Supplementary Fig. 1). Methylation of *ZNF471* promoter of SIL (LSIL and HSIL) at specific CpG sites (positions: 6, 7, and 15 to 19) was also different from normal and tumor tissues (Fig. 1c–e).

### *ZNF471* expression is silenced due to promoter methylation

**Fig. 2:**
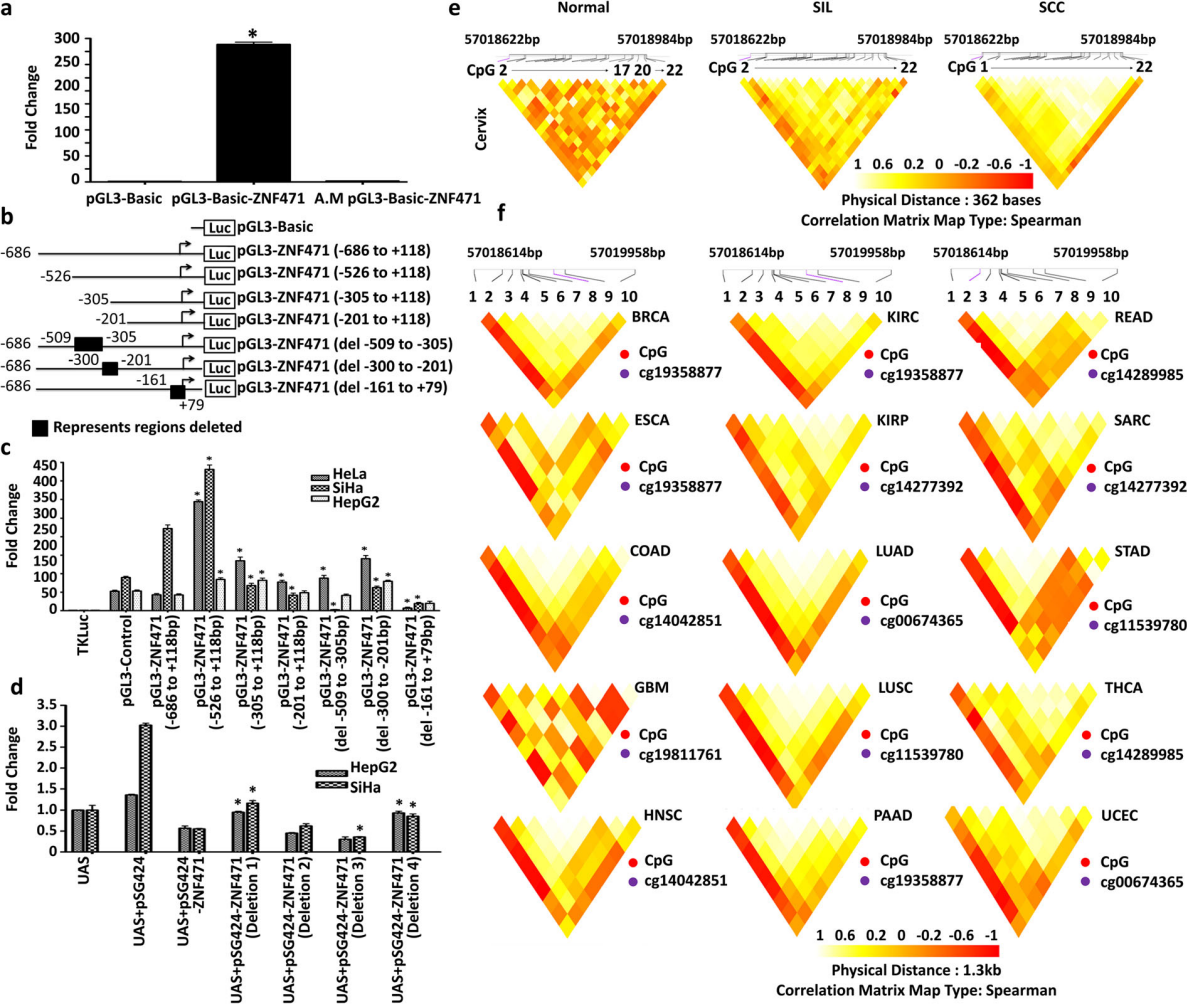
*ZNF471* promoter is regulated by methylation, acts as a transcriptional repressor, and shows co-methylation of specific CpG sites **a** To identify the promoter region of *ZNF471* and the impact of methylation on its activity, 5′ upstream region of *ZNF471* (−686 to +118) was cloned into pGL3 Basic vector with (artificially methylated (A.M) pGL3-Basic-ZNF471) and without methylation (pGL3-Basic-ZNF471) and transfected into SiHa cells, and luciferase assay was performed. The *ZNF471* promoter construct showed nearly 220-fold higher promoter activity when compared with vector alone. The region −686 to +118 bp of *ZNF471* showed robust promoter activity, which was diminished upon artificial methylation (*P*<0.0001). **b** Schematic representation of the *ZNF471* promoter used in a luciferase reporter assay. **c** To identify the core promoter of *ZNF471*, we constructed a series of 5′ deletion constructs and constructs with deletion of specific regions and transfected into SiHa, HeLa, and HepG2 cell lines. Our results demonstrated that pGL3-ZNF471 (−526 to +118 bp) showed robust promoter activity. The promoter activity of pGL3-ZNF471(−526 to +118 bp) was significantly higher than the full-length promoter construct. **d** ZNF471 acts as a transcriptional repressor. ZNF471 represses transcriptional activity in Gal4-based reporter system in SiHa and HepG2 cells. The transfection of pSG424 and pGL2-GAL4-UAS 6X showed robust luciferase activity (SiHa 5.6-fold; HepG2 3.78-fold, *P*<0.05). Transfection of vector expressing *ZNF471* (pSG424-ZNF471) significantly decreased luciferase activity (SiHa 5.6 to 0.6; HepG2 3.78 to 0.59; *P*<0.05). Histograms represent mean ± SD for three independent experiments. * *P*<0.05 by independent Student’s *t*-test was considered as statistically significant. **e** Unique co-methylation of specific CpG sites in our (normal, SIL, and SCC) tissues. The co-methylation of several CpG sites was common between SIL and tumor samples, while they were significantly different from normal samples. Further, the co-occurrence of methylation in specific CpG is unique to normal, SIL, and SCC, respectively. **f** Co-methylation analysis of multiple cancer types using the TCGA dataset. Methylation level and co-methylation patterns at specific CpG sites were significantly different in different types of cancer. Each cancer showed a distinct level of methylation at specific CpG sites differentiating each cancer. The extent of co-methylation pattern involving the CpG site is also unique for each cancer (1-cg00711090, 2-cg14289985, 3-cg14277392, 4-cg14042851, 5-cg11539780, 6-cg19811761, 7-cg00674365, 8-cg19358877, 9-cg24713204, 10-cg02823803)

*ZNF471*gene expression was significantly downregulated in SIL (*n*=10) and primary SCC tissues (*n*=20) as opposed to normal samples (*n*=20) (*P*<0.0001; Fig. 1f). SiHa, CaSki, MCF7, Jurkat, Cal27, and HT29 showed the absence of *ZNF471* expression when compared with normal diploid fibroblasts (Fig. 1 g and h). Among the CC cell lines, SiHa and CaSki showed complete absence of *ZNF471* expression. Treatment of these cell lines (SiHa and CaSki) with 5-Aza-2′-deoxycytidine restored its expression (Fig. 1h). Treatment with Aza-2′-deoxycytidine in SiHa and CaSki cells reduced DNA methylation at multiple CpG sites within the *ZNF471* promoter as analyzed by bisulfite genomic sequencing (Fig. 1i). Cloned *ZNF471* promoter constructs showed robust transcriptional activity than the vector alone, which was diminished upon artificial methylation (*P*<0.05; Fig. 2a). To identify the core promoter of *ZNF471*, deletion constructs were prepared in a luciferase expression vector. In transient transfection assays, pGL3-ZNF471 (−526 bp to +118 bp)-luc showed high luciferase activity in SiHa and HeLa as opposed to other constructs indicating the transcriptionally active region. The results were further confirmed by transfection assays using deletion constructs (Fig. 2b and c).

### *ZNF471* is a transcriptional repressor

GAL4-DBD dual-luciferase assays were performed to analyze the role of ZNF471 in transcription. The transfection of pSG424 and pGL2-GAL4-UAS 6X showed robust luciferase activity, which was decreased upon transfection with a ZNF471 expression vector (Fig. 2d). These results suggested that ZNF471 functions as a transcriptional repressor.

### Co-methylation and CpG sites

We observed a distinct pattern of methylation of specific CpG sites (CpG site 9 to CpG20) within *ZNF471* promoter in normal, SIL, and SCC tissues (Fig. 2e). CpG methylation and clustering of specific CpG sites could distinguish different cancer types (Fig. 2f), which was further confirmed by analysis of public datasets (Fig. 2f; Supplementary Fig. 2). The linear regression analysis of our data and the TCGA cohort identified a significant negative correlation between methylation and expression of *ZNF471* (*P*<0.05; Supplementary Fig. 2c; Supplementary Table 5). The Wilcoxon signed-rank test showed significantly higher methylation (*P*<0.0001) in cg14277392, cg02823803, cg24713204, cg14289985, cg00711090, and cg00674365 and strong association with CC. This was further confirmed by heat map and hierarchical clustering analysis by Euclidian distance and average linkage analysis (Supplementary Fig. 2d). The heat map of *ZNF471* methylation, along with gene expression of matched samples, showed two clusters. The first cluster consisted of samples with higher methylation and lower gene expression, while the second small cluster consisted of samples with higher *ZNF471* expression and lower methylation and/or unmethylation. The results suggest that methylation of specific CpG sites within the *ZNF471* promoter is relevant for the regulation of *ZNF471* expression and is an important indicator of carcinogenesis.

By correlation matrix and applying Spearman’s correlation approach, we identified the unique and distinct pattern of co-methylation of CpG sites in normal, SIL, and tumor (*n*=20 each; Fig. 2e). The co-methylation of CpG sites were different and distinct in SIL as opposed to tumor samples. We observed a distinct pattern of methylation of specific CpG sites (CpG site 9 to CpG20) within *ZNF471* promoter in normal, SIL, and SCC tissues (Fig. 2e). Interestingly, each cancer showed a unique and distinct pattern of co-methylation signatures whose methylation level and site varied across multiple cancers with the potential to distinguish different cancer types (Fig. 2f; Supplementary Fig. 2). From a clinical perspective, mapping co-methylated CpG sites can be useful for (i) patient stratification and (ii) fingerprinting cancer. From a biological perspective, co-methylation at multiple CpG sites may be necessary for complete silencing or long-lasting effect of methylation on the silencing of the gene.

### Diagnostic significance of *ZNF471* methylation

To assess the clinical utility, we performed sensitivity, specificity, and ROC analyses for 22 CpG sites (from −590 to −228 bp) from our results (Supplementary Table 6) and two CpG sites (from −468 to +245) of public datasets (cg14289985 and cg24713204) (Supplementary Table 7) which fall under the region sequenced in our study (CpG island no 77 located in chromosome 19). The principal component analysis (PCA) showed that cg14289985 distinguished normal, LSIL, HSIL, and SCC tissues (Supplementary Fig. 2e). Methylation of cg24713204 distinguished normal, SIL, and SCC samples (Supplementary Fig. 2e). However, methylation level at cg24713204 did not distinguish LSIL from HSIL (Supplementary Fig. 2e). Besides, the methylation levels of cg14289985 and cg24713204 significantly correlated with various histological grades of CC (Supplementary Fig. 2f). Comparative ROC analysis of our samples showed AUCs of 0.70, 0.90, and 0.86 for normal vs. SIL, normal vs. SCC, and SIL vs. SCC, respectively (Supplementary Table 6). ROC analysis of 5 independent data sets further confirmed our results (Supplementary Table 7). Taken together, our results showed the utility of *ZNF471* promoter methylation for early diagnosis of CC. Similarly, individuals with higher *ZNF471* promoter methylation showed significantly shorter OS and RFS by Log-rank (Mantel-Cox) univariate test using the Kaplan–Meier method (*P* ≤ 0.05; Supplementary Figs. 2g and 3). Besides, lower expression was associated with significantly shorter OS (*P* ≤ 0.05; Supplementary Fig. 3a). The methylation level of *ZNF471* was significantly correlated with clinicopathological characteristics such as stage, grade, lymph node metastasis, and histological type (Supplementary Fig. 4).

### *ZNF471* inhibits the proliferation and growth of SiHa and CaSki cells

**Fig. 3:**
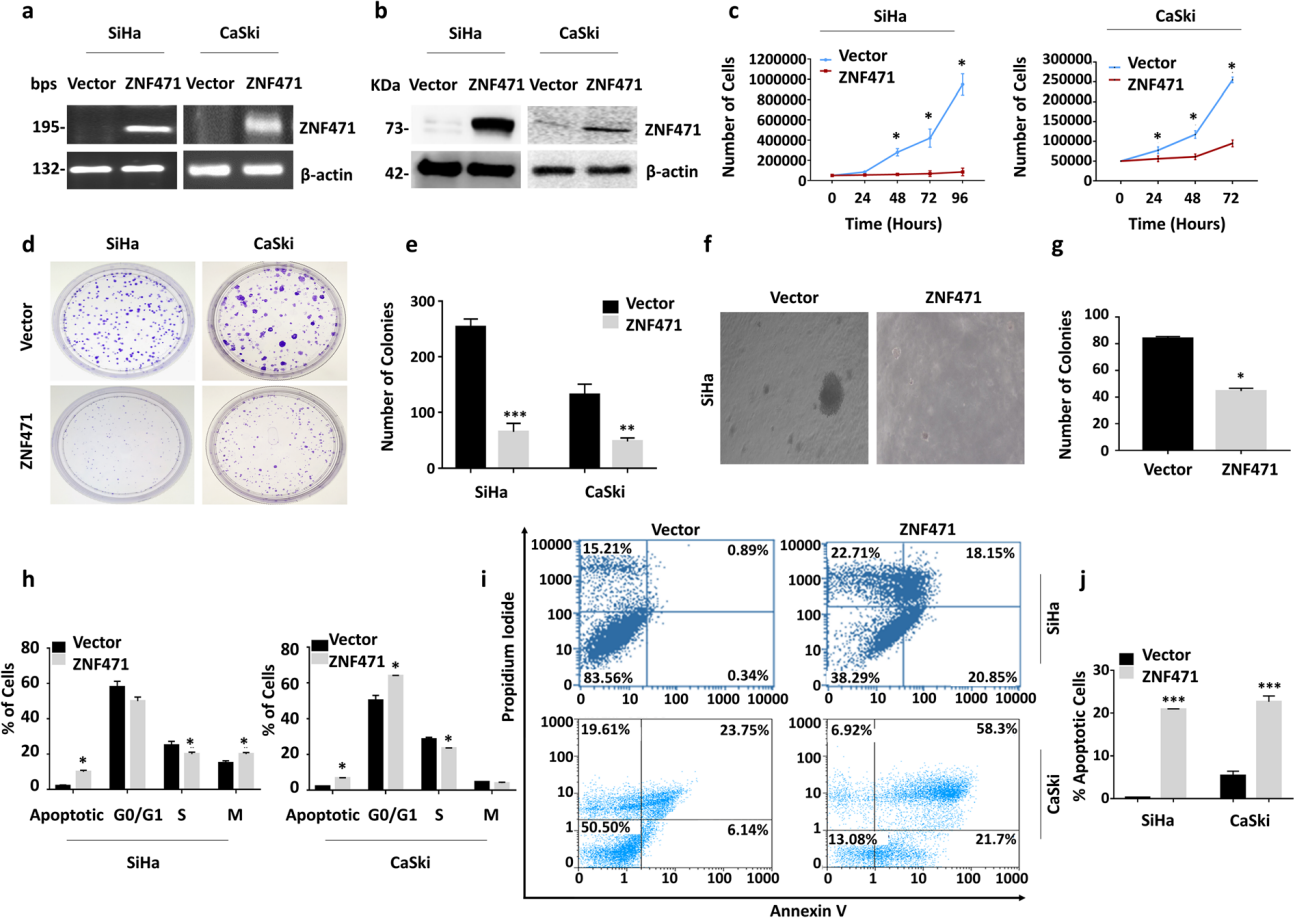
Effect of ectopic expression of *ZNF471* on tumor growth and proliferation. **a, b** RT-PCR and western blot analysis confirming the expression upon ectopic expression *ZNF471* in SiHa and CaSki cells with β-actin as an internal control. **c** Line graph showing the proliferation rate of ZNF471 expressing cells in comparison with vector control. A growth curve assay was performed between ZNF471 expressing and control cells. Ectopic expression of *ZNF471* significantly inhibited cell proliferation resulting in a delay in cell doubling time (*P*<0.05). The cell doubling analysis was performed using the doubling time calculator (http://www.doubling-time.com/compute.php). **d** ZNF471 inhibits anchorage-dependent tumor growth in vitro in SiHa and CaSki cells. **e** Quantitative analysis of colony formation. The colony formation assay showed that ZNF471 expression reduced both the number and size of the colony when compared with vector-transfected control cells (*P*<0.05). **f, g** Image of anchorage-independent soft agar colony-forming assay and its quantitative analysis. Our results showed that ZNF471 expression decreased the colony size and number in SiHa cells when compared with control cells (*P*<0.05). **h** The cell cycle analysis in empty vector and ZNF471-transfected SiHa and CaSki cells. Flow cytometry analysis of SiHa cells expressing ZNF471 revealed significant decrease in the number of cells in S phase with a concurrent increase in a number of cells at G2/M phase when compared with control cells (S phase 25.5 ± 0.3 vs. 19.7 ± 1.4; G2/M 11.8±1.1 vs. 18.8±1.2; *P*<0.05). In CaSki cells, ZNF471 expression induced apoptosis and growth arrest at G0/G1 phase (G0/G1 phase 50.255 ± 2.77742 vs 64.025 ± 0.190919; S phase 28.59 ± 1.004092 vs 23.45 ± 0.212132). **i, j** ZNF471 overexpression induces apoptosis in ZNF471 expressing SiHa and CaSki cells. Percentages of Annexin-V positive cells were significantly higher in ZNF471 expressing cells when compared with vector-transfected cells (SiHa 0.305 ± 0.0495 vs 20.92 ± 0.09899; CaSki 6.145 ± 0.982878 vs 21.7 ± 1.357645). The bar graphs represent the mean ± SD of triplicate experiments performed in duplicates. * *P*<0.05 by independent Student’s *t*-test was considered as statistically significant

Expressions of *ZNF471* upon stable transfection in SiHa and CaSki cells were confirmed by RT-PCR and western blotting (Fig. 3 a and b). *ZNF471* overexpression reduced cell proliferation by increasing the cell doubling time (SiHa-ZNF471, 48.52 h; CaSki-ZNF471, 72 h) compared with control cells (SiHa-Vector, 21.04 h; CaSki-Vector, 30.63) (*P*<0.05; Fig. 3c). The anchorage-dependent assay showed the tumor growth suppressive function of ZNF471 in SiHa and CaSki cells (*P*<0.005; Fig. 3 d and e). The findings were further confirmed by anchorage independent assay in SiHa cells (*P*<0.005; Fig. 3 f and g).

### *ZNF471* expression changes cell size, shape, and morphology, induces actin cytoskeletal rearrangement, and induces cell cycle arrest

**Fig. 4:**
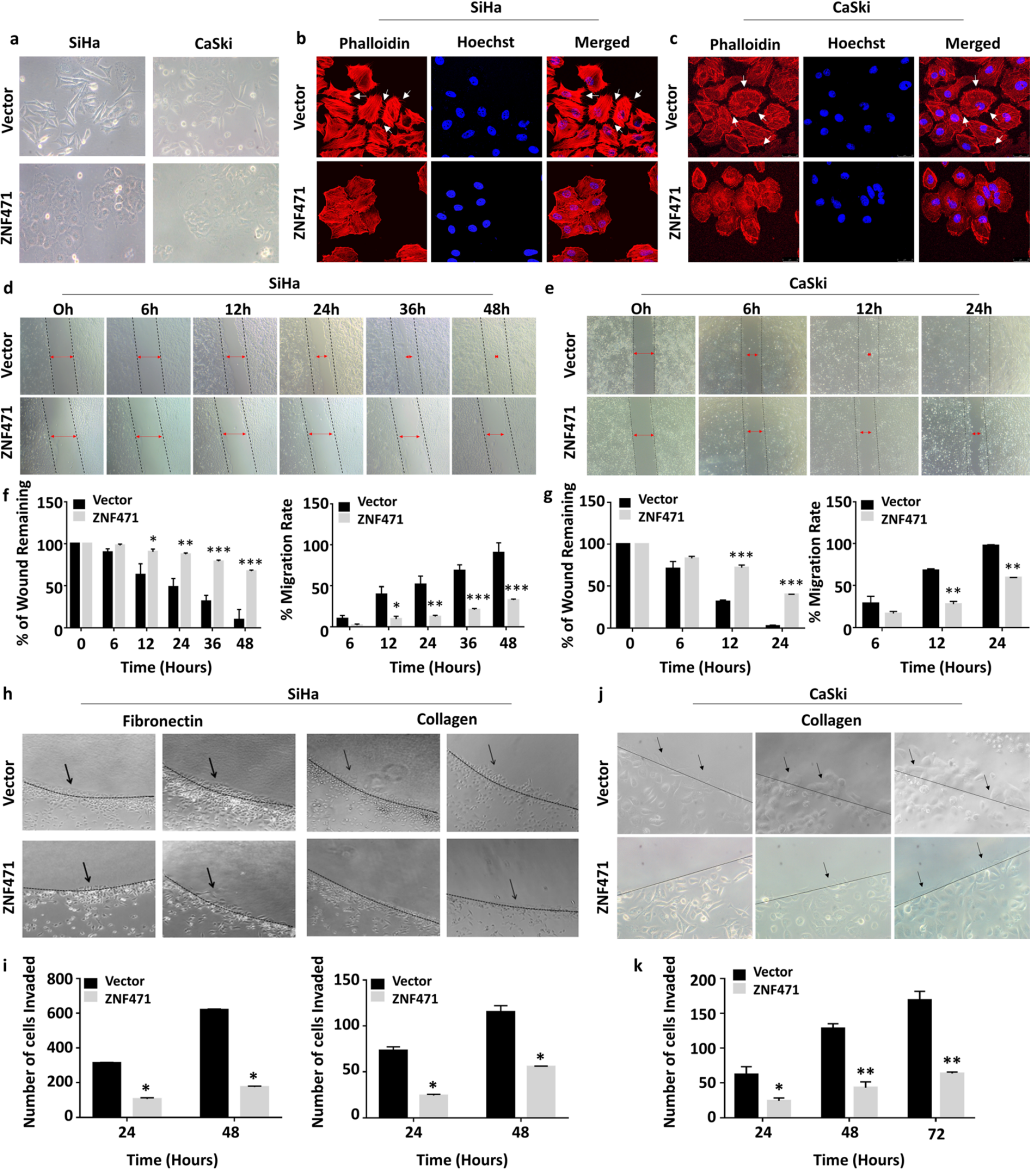
ZNF471 expression induces morphological change and inhibits migration and invasion of SiHa and CaSki cells in vitro. **a** Bright field microscopic images showing cell morphology of ZNF471 expressing and control SiHa and CaSki cells (×20 magnification). **b, c** Actin-phalloidin staining showing rearrangement of actin fibers leading to the increased cell-to-cell adhesion and decreased lamellipodia and filopodia in ZNF471 expressing cells when compared with control cells. Lamellipodia are indicated by arrows (×63 magnification). Wound-healing assay was performed to identify the effect of ZNF471 on cell migration in vitro. **d** Bright field microscopic images of wound healing assay in SiHa cells (×10 magnification). The wound was completely closed in control cells by 48 h. However, in ZNF471 expressing SiHa cells, wound was still intact even after 48 h. **e** Wound healing assay in CaSki cells (×10 magnification). The wound was completely closed in control cells by 24 h. However, in ZNF471 expressing CaSki cells, wound was still intact even after 24 h. **f** Bar graphs showing the quantitative analysis of cell migration in SiHa cells. The percentage of wound remaining was significantly more upon ectopic expression of *ZNF471* in SiHa cells when compared with vector-transfected control cells (6 h, 97.9% vs. 89.9%; 12 h, 90.2% vs. 62.9%; 24 h, 87.3% vs. 48.2%; 36 h, 78.9% vs. 31.5%; 48 h, 67.2% vs. 9.7%; *P*<0.05). The cell migration rate was significantly inhibited in ZNF471 expressing cells as opposed to control cells (6 h, 2.1% vs 10.1%; 12 h, 9.7% vs. 39.6%; 24 h, 12.6% vs. 51.7%; 36 h, 21.0% vs. 68.4%; 48 h, 32.7% vs 90.2%; *P*<0.05). **g** Bar graphs showing the quantitative analysis of cell migration in CaSki cells. The percentage of wound remaining was significantly more upon ectopic expression of *ZNF471* in CaSki cells when compared with vector-transfected control cells (6 h, 70.9% vs 83.07%; 12 h, 31.8% vs 71.9%; 24 h, 2.4% vs 40.4%; *P*<0.05). The cell migration rate was significantly reduced in ZNF471 expressing CaSki cells as opposed to control cells (6 h, 29.04% vs 16.9%; 12 h, 68.1% vs 28.1%; 24 h, 97.5% vs 59.5%; *P*<0.05). **h** ZNF471 inhibits invasion of SiHa cells toward fibronectin and collagen-coated agarose spots (×10 magnification). **i** ZNF471 expressing cells were found to be more concentrated near the agarose junction, and very few cells penetrated the agarose spot even after 48 h (24 h, 109.25±9.54 vs 317.25±12.72; 48 h, 187±11.42 vs 317.25±14.7; *P*=0.0029 and *P*=0.003) for fibronectin and (24 h, 26±3.2 vs 73±6.36; 48 h, 52±2.47 vs 117±2.76; *P*=0.01 and *P*=0.001) for collagen. **j** ZNF471 inhibits invasion of CaSki cells toward collagen-coated agarose spot (×20 magnification). **k** Bar graph representing the number of CaSki cells invading the agarose spot (24 h, 62±11 vs 24±4.2; 48 h, 128±7 vs 43±8.5; 72 h, 169±13 vs 63.5±2.1; *P*<0.05). The bar graph represents the mean ± SD of triplicates experiments performed in duplicates. * *P*<0.05 by independent Student’s *t*-test was considered as statistically significant

Cell cycle analysis after *ZNF471* overexpression showed a significant decrease in S-phase cells with a concurrent increase in G2/M phase cells as opposed to control cells (*P*<0.05; Fig. 3h). Ectopic expression of *ZNF471* in SiHa cells showed significantly higher apoptotic cells compared with vector-transfected control cells (0.34% vs 20.85%; Fig. 3i, j). In CaSki cells, ZNF471 overexpression induced apoptosis (6.14% vs 21.7%; Fig. 3i, j) and growth arrest at G0/G1 phase when compared with mock transfected cells (*P*<0.05; Fig 3h). Overexpression of *ZNF471* markedly changed cell morphology and actin cytoskeleton arrangement, increased cell size, organized cell-cell contact, and reduced the number, length, and arrangement of lamellipodia as opposed to control cells (Fig. 4a–c)

### *ZNF471* overexpression inhibits migration and invasion

Overexpression of *ZNF471* significantly slowed the migration of SiHa and CaSki cells, which was evident from the inability to induce wound closure when compared to controls (*P*<0.05; Fig. 4d–g). *ZNF471* expressing SiHa cells significantly inhibited invasion into fibronectin and collagen type I coated agarose spots (*P*<0.05; Fig. 4 h and i). *ZNF471* expression in CaSki cells significantly inhibited invasion into collagen type I coated agarose spots (*P*<0.05; Fig. 4 j and k).

### *ZNF471* inhibits tumor growth in vivo

**Fig. 5:**
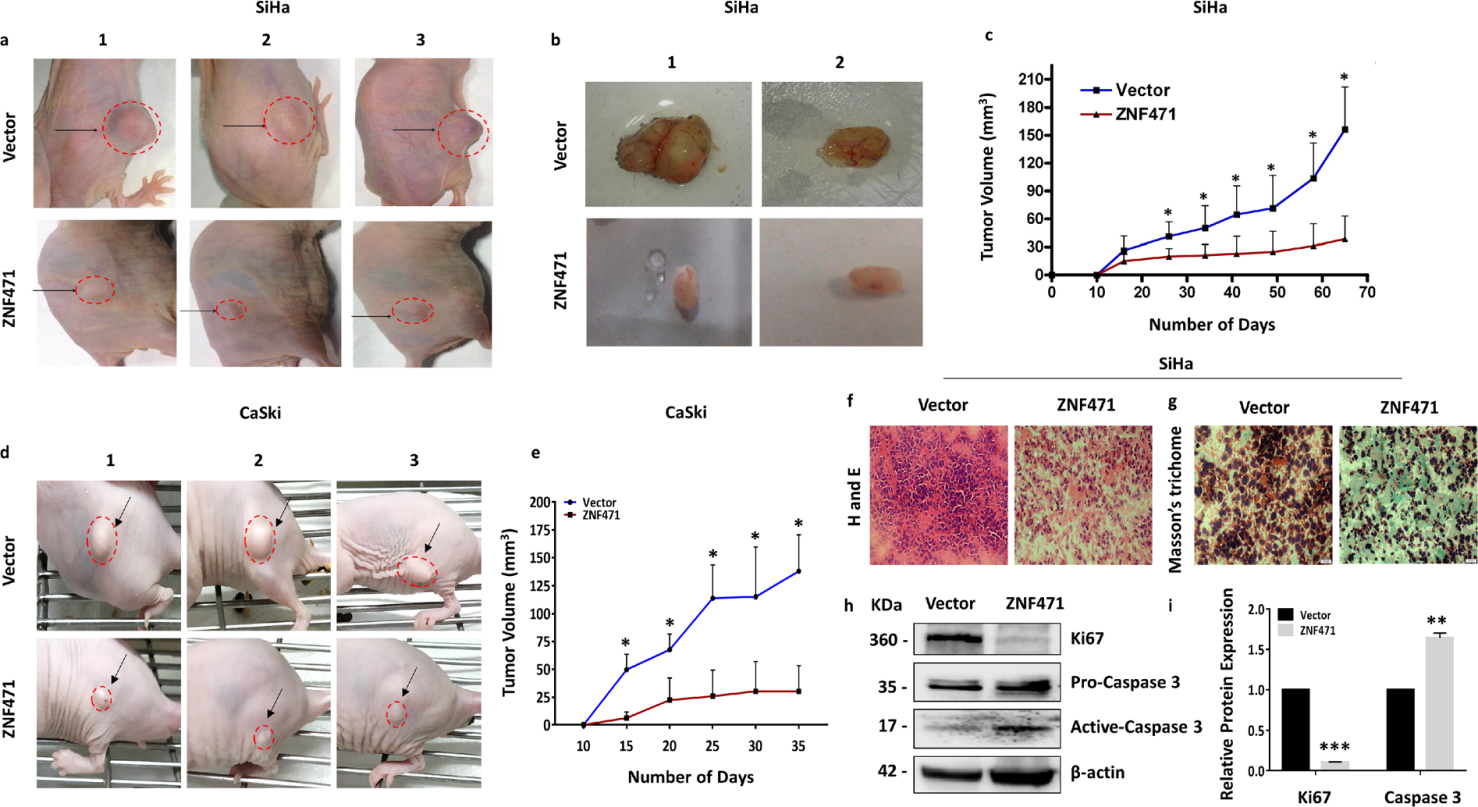
ZNF471 inhibits tumor growth in vivo in nude mice. **a** Images of tumor growth in nude mice injected subcutaneously with empty vector or ZNF471 transfected SiHa cells. **b** Image showing the blood vessel in the tumor isolated from mice received control cells as opposed to ZNF471 expressing SiHa cells. **c** Tumor growth curve of control and ZNF471-expressing SiHa cells. The control cells formed significantly larger and progressive tumors when compared with ZNF471 expressing cells. **d** Images of CaSki xenografts. **e** ZNF471 expression significantly inhibited growth of CaSki xenograft. **f** Image of the H&E staining of the tumor xenografts showing the distinct histological difference between control and ZNF471 expressing SiHa cells. ZNF471 expression induced morphological change, increased size, decreased cell scattering, and decreased tumor cell density with lesser pleomorphic cells and reduced number of abnormal mitosis. In contrast, ZNF471 negative cells were predominantly/increasingly pleomorphic, spindle shaped, showing prominent nuclei with the altered nucleus to cytoplasmic ratio and abnormal mitosis. **g** Image of Masson’s trichrome staining of tumor xenograft with and without ZNF471 expression in SiHa cells. ZNF471 expression inhibited collagen degradation in vivo. “*” indicates statistical significance (*P < 0.05). **h** Western blot analysis of Ki67 and Caspase 3 in SiHa xenograft samples. **i** Bar graph showing significant reduction in Ki67 expression and elevation in active Caspase-3 level in ZNF471 expressing CaSki cells

*ZNF471* expression significantly suppressed in vivo tumor growth in nude mice grafted with SiHa (Fig. 5a–c) and CaSki cells (Fig. 5 d and e)*.* H & E staining of tumor tissue cryosection (5 μm thickness) revealed visible histological differences between the tumors developed from mice receiving *ZNF471* expressing cells and control SiHa cells (Fig. 5f). Masson’s trichrome staining displayed a significant increase in collagen in *ZNF471* expressing tumors as opposed to control tumors (Fig. 5g). Ectopic expression of *ZNF471* reduced the expression of Ki67 and activated Caspase 3 when compared with vector-transfected control xenograft cells (Fig. 5 h and i).

### ZNF471 is essential to inhibit EMT in SiHa cells

**Fig. 6:**
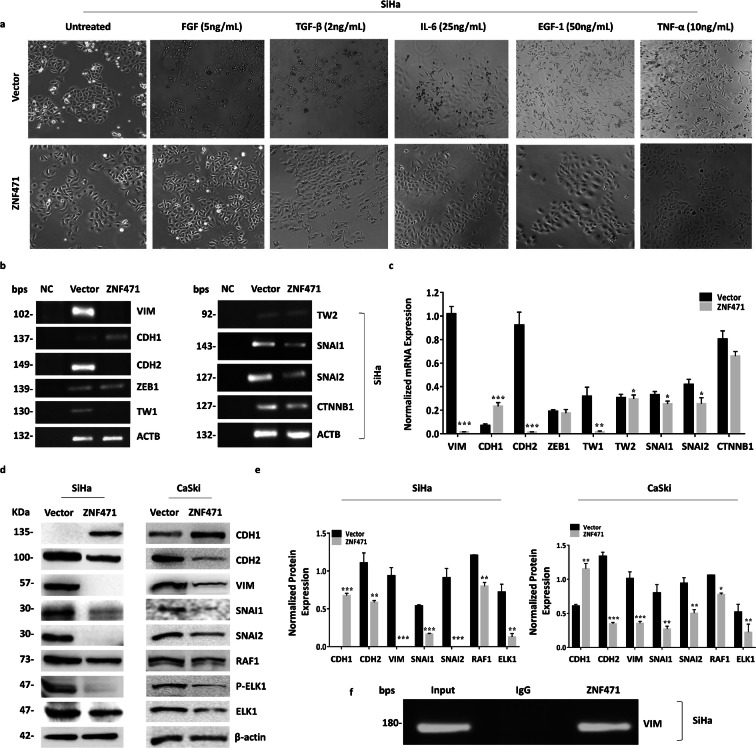
*ZNF471* inhibits the expression of EMT markers. **a** Bright field microscopic images of SiHa cell morphology in the presence of EMT inducers. The expression of ZNF471 did not show any morphological changes except for TGF-β, while control cells showed mesenchymal phenotypes with all the EMT inducers. Control and ZNF471 expressing SiHa cells were treated with FGF-2 (5 ng/mL), TGF-β (2 ng/mL), IL-6 (25 ng/mL), EGF1 (50 ng/mL), and TNF-α (10 ng/mL) for 72 h to assess the EMT. **b** RT-PCR analysis of the basal level of epithelial and mesenchymal markers in control and ZNF471 expressing SiHa cells. The expression ZNF471 significantly up-regulated *CDH1*, inhibited *VIM*, *CDH2*, and *TW1*, and marginally reduced *SNAI1*, *SNAI2*, and *CTNNB1* without changing *ZEB1* and *TW2* at mRNA level. **c** Bar graph representing the densitometry analysis of epithelial and mesenchymal marker levels. β-actin was used for normalization as an internal control in RT-PCR experiments. “*” indicates statistical significance (*P < 0.05). **d** Western blot analysis for EMT associated proteins. Presence of ZNF471 significantly downregulated mesenchymal markers (CDH2, VIM, ELK1, and RAF1) and EMT transcription factors (SNAI1, SNAI2) with the concomitant increase in the level of CDH1 in SiHa and CaSki cells. **e** Bar graphs showing normalized protein levels of EMT associated proteins in SiHa and CaSki cells. **f** Gel image of ChIP-PCR indicating direct binding of ZNF471 to *VIM* promoter

Treatment of SiHa cells with FGF-2, IL-6, EGF-1, and TNF-α resulted in elongation of the cells, loss of cell-to-cell adhesion, and increased cell scattering. *ZNF471* expressing cells did not show any appreciable morphological changes. TGF-β treatment resulted in morphological changes both in control and *ZNF471* expressing cells (Fig. 6a). Overexpression of *ZNF471* in SiHa cells resulted in the up-regulation of *CDH1* and downregulation of *VIM*, *CDH2*, and *TW1* when compared with control cells by RT-PCR analysis (*P*<0.05; Fig. 6 b and c). CDH1 protein level was significantly elevated, whereas CDH2, VIM, SNAI1, SNAI2, RAF1, and p-ELK1 levels were reduced upon *ZNF471* overexpression in SiHa and CaSki cells (Fig. 6 d and e). As per Pearson correlation analysis, *ZNF471* promoter methylation were positively correlated with *CDH1* while negatively correlated with the expression of *CDH2*, *VIM*, *ZEB1*, *TWIST1*, *SNAI1*, and *CTNNB1* (Supplementary Fig. 5; Supplementary Table 8). Further, expression analysis of *ZNF471* along with one epithelial and 9 mesenchymal markers in the GSE9750 dataset identified the downregulation of *ZNF471* and *CDH1* with concomitant up-regulation of *ZEB1* and *TWIST1* in tumor tissues when compared with normal samples (*P*<0.05; Supplementary Fig. 6).

### ZNF471 directly binds to the VIM promoter

By luciferase reporter assay, RT-PCR, and western blotting, we showed ZNF471 as a transcriptional repressor and inhibitor of EMT. To identify the target genes, we predicted the ZNF471 binding sites, and the results were confirmed by ChIP-PCR. Among the EMT genes predicted, ZNF471 was predicted to bind to *VIM*. Our ChIP-PCR results further confirmed the same in SiHa cells (Fig. 6f).

### ZNF471 negatively regulates key members of the Wnt/ β-catenin signaling pathway

**Fig. 7:**
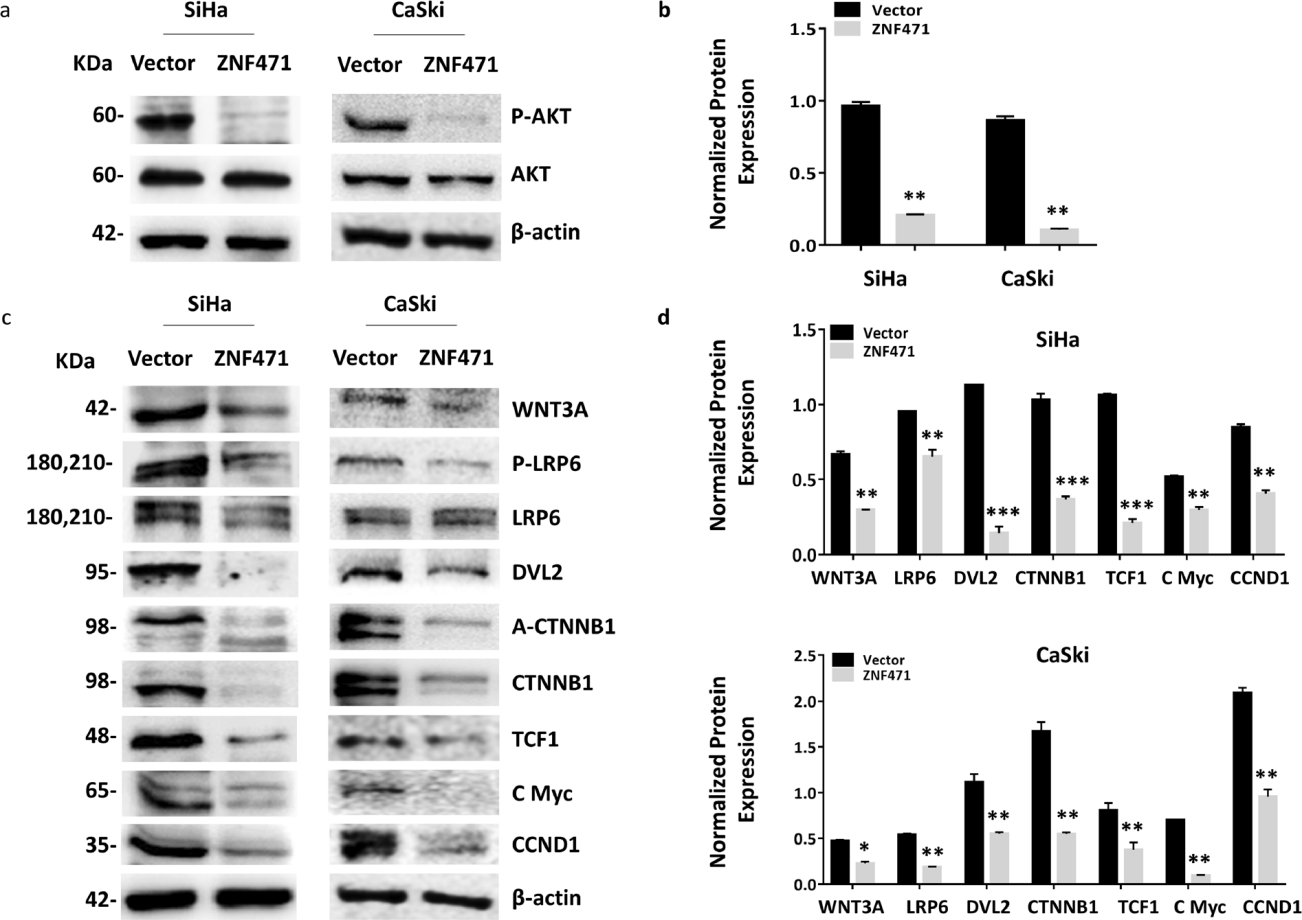
ZNF471 negatively regulates Wnt signaling. **a** Western blot analysis revealed the inhibition of AKT phosphorylation in the presence of ZNF471 in SiHa and CaSki cells. **b** Bar graph showing reduced levels of p-AKT in ZNF471 expressing SiHa and CaSki cells. **c** Relative levels of Wnt associated proteins showing negative regulation of WNT3A, LRP6, CTNNB1, DVL2, TCF1, C-MYC, and CCND1 in ZNF471 expressing cells when compared with control SiHa cells. **d** Bar graph showing normalized protein levels of Wnt associated proteins in SiHa and CaSki cells

Overexpression of *ZNF471* in SiHa and CaSki inhibited AKT1 (Ser473) phosphorylation without changing the level of total protein (Fig. 7 a and b). *ZNF471* repressed key members of Wnt/ β-catenin pathway in SiHa and CaSki cells. Western blot analysis revealed the downregulation of Wnt protein (WNT3A), Wnt receptor (p-LRP6), active form of CTNNB1, DVL2, TCF1, and Wnt activated target genes (c-MYC, CCND1) in *ZNF471* expressing cells when compared with empty vector-transfected SiHa and CaSki cells (Fig. 7 c and d).

## Discussion

Several tumor suppressor genes are hypermethylated and hence silenced in cancers (Ambrosini et al. 2018; Bhat et al. 2017a; Bhat et al. 2017b; Bhat et al. 2016; Hanley et al. 2017; Kabekkodu et al. 2014; Martin et al. 2015). Here, we showed evidence for distinct methylation marks conserved across clusters of specific CpG sites in *ZNF471* within various clinical diagnostic categories such as SIL and SCC, thus facilitating tumor typing. One of the significant findings of our study is the identification of CpG methylation and the co-occurrence of specific CpG sites during CC progression. Frequent methylation and co-occurrence of specific CpG sites could distinguish between different cancers (Vidal et al. 2017). These results suggest that methylation of CpG sites occurs differentially during cellular transformation and can play a significant role in the pathogenesis of CC. However, the exact molecular mechanism of site-specific CpG methylation and its relevance to carcinogenesis remains to be explored. Epigenetic changes in cancer are extensively studied for diagnosis, prognosis, and therapeutic interventions (Bhat et al. 2016; Gomih et al. 2018; Wheeler 2007; Zhang and Huang 2017). Since aberrant DNA methylation occurs very early during tumorigenesis, it could, therefore be used as an early diagnostic marker (Bhat et al. 2016; Das and Singal 2004; Karayan-Tapon et al. 2010; Wentzensen et al. 2009). The panel of distinct CpG sites identified in our study was found to be universal, robust, specific, and sensitive for the discrimination of clinical stages of CC and as a surrogate marker for early diagnosis and prognosis.

We have identified methylation of specific CpG sites and their co-occurrence during progression from SIL to SCC. These results indicate that co-methylation of specific CpG sites might be an essential event during carcinogenesis and can be useful for tumor classification. Although further validation is required, bisulfite sequencing of specific CpG sites within the *ZNF471* promoter or expression analysis by RT-PCR may add significant prognostic value at the time of CC diagnosis, treatment, and decision making. Cao et al. (2018) reported that *ZNF471* is downregulated by hypermethylation and acts as a tumor suppressor gene in gastric cancer (Stephen et al. 2012). We had previously reported hypermethylation of *ZNF471* in oral squamous cell carcinoma of the tongue (Bhat et al. 2017a). While we screened the methylation status of 22 CpG sites (−590 to −228 bp with respect to TSS), Cao et al. (2018) screened 8 CpG sites (−74 to +30 with respect to TSS). The methylation range across the 8 CpG sites was approximately 65 to 80% in tumor samples and 45 to 55% in adjacent normal samples. In our study, methylation in normal and tumor samples wherein the range of 0 to 41% (average 13.23 ± 2.587) and 3.1 to 94% (average 59.36 ± 7.154), respectively (Bhat et al. 2017a; Stephen et al. 2012).

Among the 22 CpG sites tested, CpG sites from 9 to 20 showed significant co-methylation in tumor samples as opposed to normal and SIL. Spearman’s correlation identified that the co-methylated CpG sites are significantly different between SIL and cervical tumors. We further extended this observation to investigate the utility of co-methylation of CpG sites for distinguishing different cancers using TCGA datasets. Our analysis suggested that co-methylation of CpG sites were different in different cancers with the potential to distinguish cancer. For example, in ESCA, cg14042851, cg11539780, and cg19811761 were significantly co-methylated when compared with GBM while cg00674365 and cg19358877 were significantly co-methylated in GBM as opposed to ESCA data set. Methylation in cg11539780, cg14042851, cg19811761, cg19358877, and cg00674365 showed significantly higher co-methylation in KIRC while cg11539780, cg19811761, cg00674365, cg19358877, and cg24713204 were co-methylated in KIRP.

Additionally, we have also identified unique CpG sites whose methylation is highly correlated with the methylation status of other CpG sites and is unique to each cancer and can also be used for distinguishing different cancers from each other. For example, the highly correlated co-methylated CpG site is cg19358877 in BRCA, cg14042851 in COAD, cg19358877 in ESCA, cg19811761 in GBM, and cg14277392 in KIRP. Similarly, it may also be used for distinguishing different histological grades of cancer. For instance, cg00674365 and cg11539780 are highly correlated with co-methylated CpG sites in lung adenocarcinoma (LUAD) and lung squamous cell carcinoma (LUSC), respectively. Similarly, cg19358877 and cg14277392 can be used for distinguishing kidney renal clear cell carcinoma (KIRC) and kidney renal papillary cell carcinoma (KIRP), respectively (Supplementary Table 9). However, further detailed studies are required before a further conclusion is drawn.

Mechanistically, ectopic expression of ZNF471 significantly inhibited the growth, proliferation, migration, and invasion of SiHa and CaSki cells in vitro and tumor growth in vivo. This may be linked to the induction of apoptosis and anti-proliferative effect due to arrest at S phase of the cell cycle. Similar to our study, Xiao et al. (2014) showed that ZNF545 inhibits cell proliferation in breast cancer by inducing apoptosis through c-Jun/AP1, BAX, p53, and Caspase 3-mediated pathways (Xiao et al. 2014). Yu et al. (2013) showed ZNF331 inhibits the growth and invasiveness of gastric cancer cells (Yu et al. 2013). Thus, our data strongly suggest that ZNF471 might act as a tumor suppressor by inhibiting the genes involved in proliferation, growth, migration, and invasion.

The treatment of ZNF471 overexpressed cells with EMT inducing agents such as EGF-1, TGF-β, TNF-α, FGF-2, and IL-6 did not induce significant morphological changes except for TGF-β in SiHa cells. However, treatment of control cells readily induced morphological changes similar to that of mesenchymal cells. Analysis of basal level expression of EMT markers by RT-PCR showed up-regulation of *CDH1* with concomitant inhibition of *VIM*, *CDH2*,and TW1 and a marginal reduction in the expression of *SNAI1*, *SNAI2*, and *CTNNB1* in ZNF471 overexpressing cells as opposed to vector-transfected control SiHa cells. Thus, reduced migration and invasion by ZNF471 involves inhibition of EMT and Wnt signaling in SiHa and CaSki cells as analyzed by western blotting. The interconnected Ras, PI3K, and ERK pathways regulate diverse cellular functions such as cell proliferation, differentiation, apoptosis, cell cycle, cell migration, and invasion through possibly targeting EMT associated genes. PI3K, ERK, Rac1/Cdc42 are the main effectors of Ras signaling regulating cell growth, cell cycle entry, cell survival, cytoskeleton reorganization, and cell motility (Lee et al. 2006). The PI3K pathway contributes to metastatic cell motility through the activation of Rac1 and Cdc42 (Yates 2011) and evades cell death by activating AKT-mediated survival pathways. Our results show that ZNF471 acts as a transcription repressor and inhibits EMT signaling via directly targeting *VIM* (Castellano and Downward 2011; Kolsch et al. 2008).

EMT induction is associated with the downregulation of *CDH1* and up-regulation of *VIM*, *CDH2*, and EMT transcription factors (EMT-TFs) such as *ZEB1*, *ZEB2*, *TW1*, *TW2*, *SNAI1*, and *SNAI2* (Cao et al. 2018; Lamouille et al. 2014; Larue and Bellacosa 2005). Cancer cells have multiple mechanisms of *CDH1* downregulation, which appears to be a prerequisite for EMT as *CDH1* has multiple binding sites for EMT-TFs. Moreover, the EMT promoting factors have shown to increase the expression of *ZEB1*, *ZEB2*, *TW1*, *TW2*, *SNAI1*, and *SNAI2* through RAS, SMAD, NOTCH, AKT, and MAPK signaling (Garg 2013). TWIST1 (TW1) were shown to promote migration and invasion via downregulation of *CDH1* and up-regulation of *CDH2* and *VIM* (Davidowitz et al. 2014; Mirantes et al. 2013) contributing to the loss of cell polarity, cell-to-cell contact, increased motility, and migration.

**Fig. 8:**
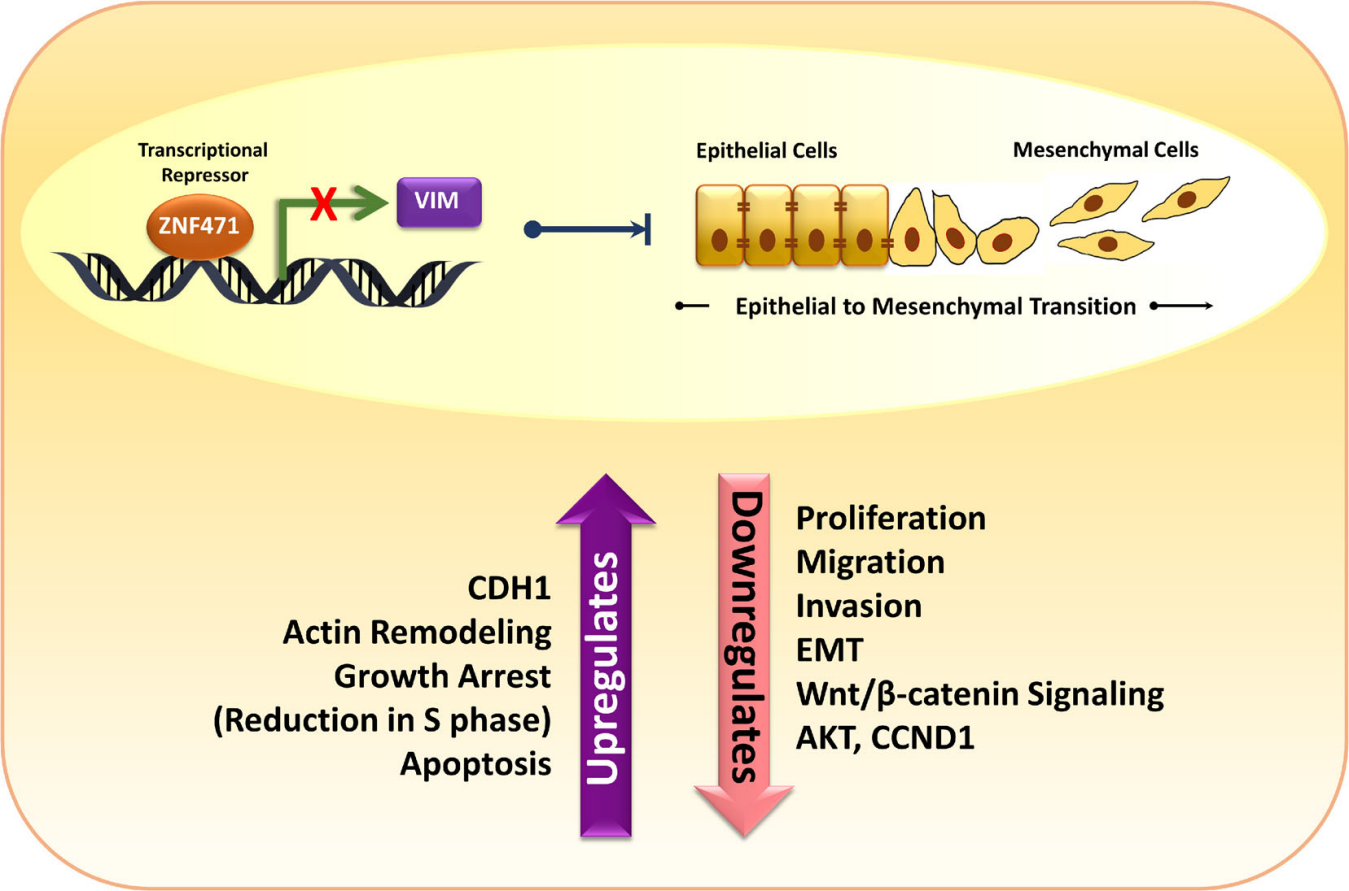
Proposed model for *ZNF471* mediated tumor suppression in cervical cancer

In summary, we performed a comprehensive investigation of the cause and consequences of *ZNF471* downregulation in CC. We showed that *ZNF471* is a methylation-regulated gene with promoter methylation driving its expression. Despite this, more detailed studies are required to identify the possible impact of methylation of enhancers and super-enhancers on *ZNF417* expression, if any. We identified ZNF471 as a novel potential tumor suppressor gene in CC that inhibits cancer cell growth, proliferation, invasion, and metastasis by arresting cell cycle progression and induction of EMT by up-regulation of CDH1 and downregulation of CDH2, VIM, and TW1 (Fig. 8). Besides, we have identified co-methylation of specific CpG sites of *ZNF471* promoter in CC, which can be used for distinguishing between cancers as well as different stages within a given cancer. Taken together, *ZNF471* methylation and expression analysis could serve as a potential biomarker for early detection and for developing novel therapeutic strategies for CC.

## Supplementary information

ESM 1 (PNG 1145 kb)

High resolution image (TIF 12970 kb)

ESM 2 (PNG 1127 kb)

High resolution image (TIF 21456 kb)

ESM 3 (PNG 1060 kb)

High resolution image (TIF 5014 kb)

ESM 4 (PNG 391 kb)

High resolution image (TIF 4365 kb)

ESM 5 (PNG 183 kb)

High resolution image (TIF 555 kb)

ESM 6 (PNG 1031 kb)

High resolution image (TIF 3726 kb)

ESM 7 (DOCX 27 kb)

ESM 8 (DOCX 27 kb)

ESM 9 (DOCX 15 kb)

ESM 10 (DOCX 52 kb)

ESM 11 (DOCX 22 kb)

ESM 12 (DOCX 15 kb)

ESM 13 (DOCX 48 kb)

ESM 14 (DOCX 36 kb)

ESM 15 (DOCX 15 kb)

ESM 16 (DOCX 15 kb)

## Data Availability

The authors declare that the data supporting the findings of this study are available within the paper and its Supplementary Information files. All other data are available from the corresponding author upon reasonable request.
