# Supplementary figures and images for: ZNF471 modulates EMT and functions as methylation regulated tumor suppressor with diagnostic and prognostic significance in cervical cancer
Source: Cell Biol Toxicol. 2021 Feb 10;37(5):731–49. doi: 10.1007/s10565-021-09582-4 (PMC8490246; doi:10.1007/s10565-021-09582-4)

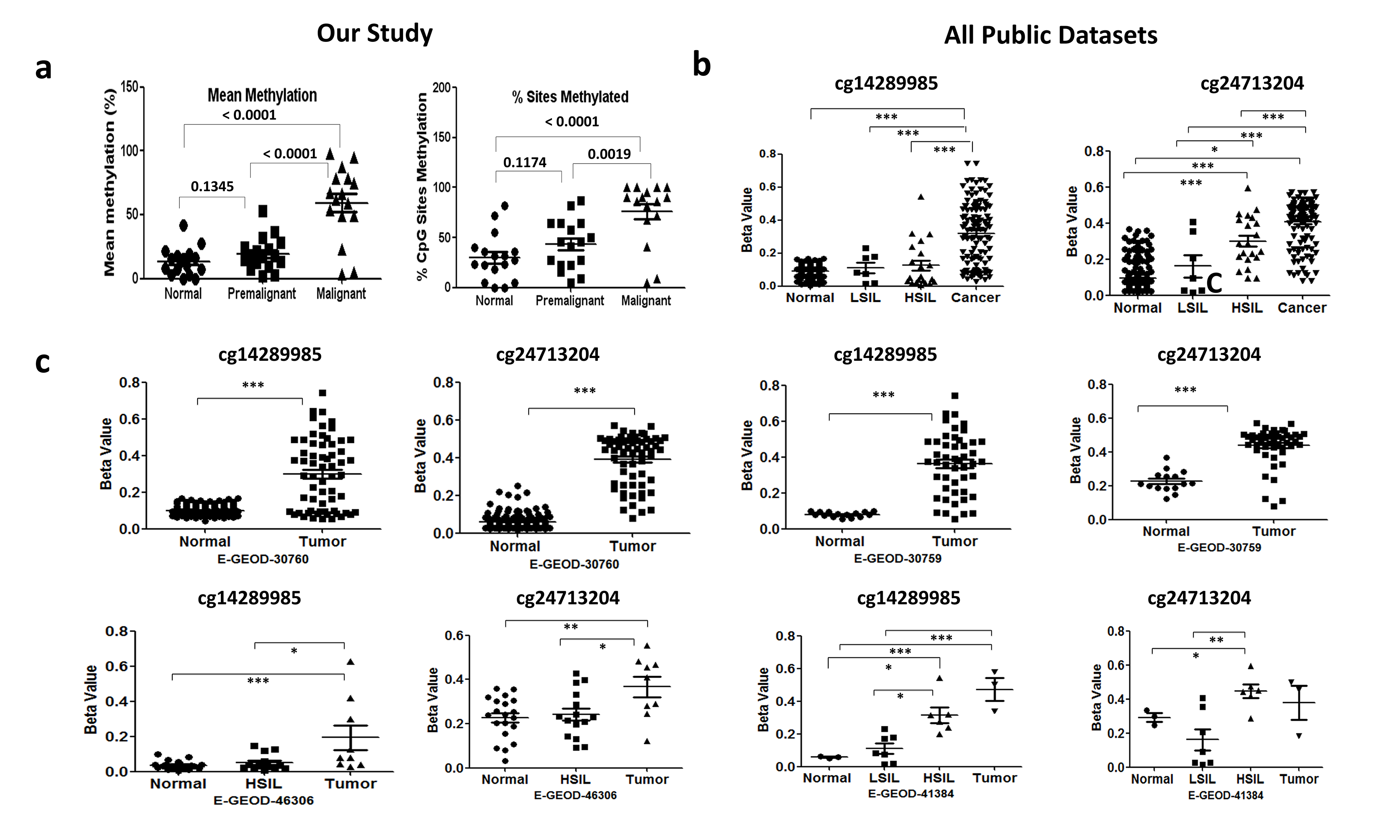

Supplement: Supplementary file 1 — (PNG 1145 kb) [file 10565_2021_9582_Fig9_ESM.png]

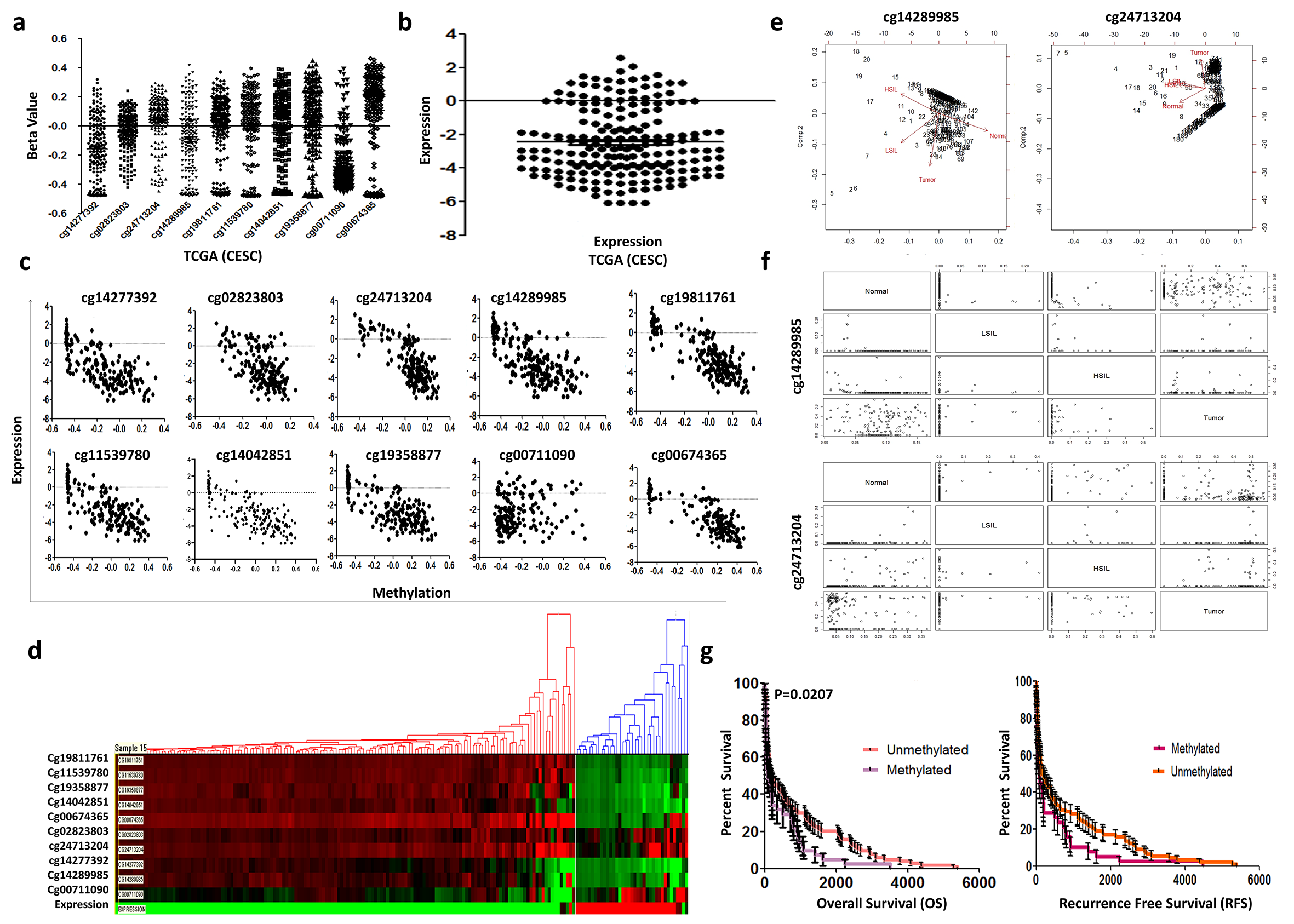

Supplement: Supplementary file 3 — (PNG 1127 kb) [file 10565_2021_9582_Fig10_ESM.png]

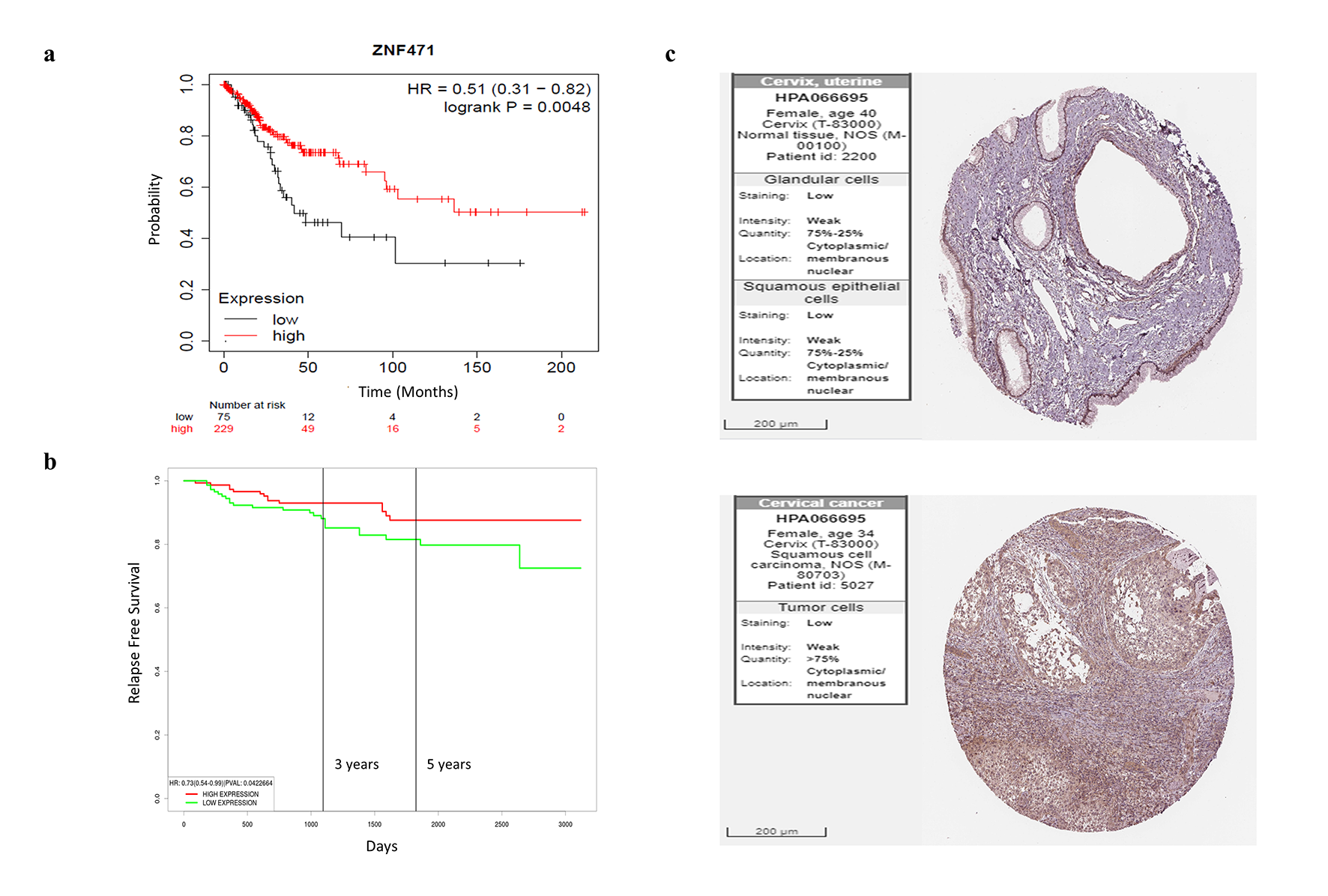

Supplement: Supplementary file 5 — (PNG 1060 kb) [file 10565_2021_9582_Fig11_ESM.png]

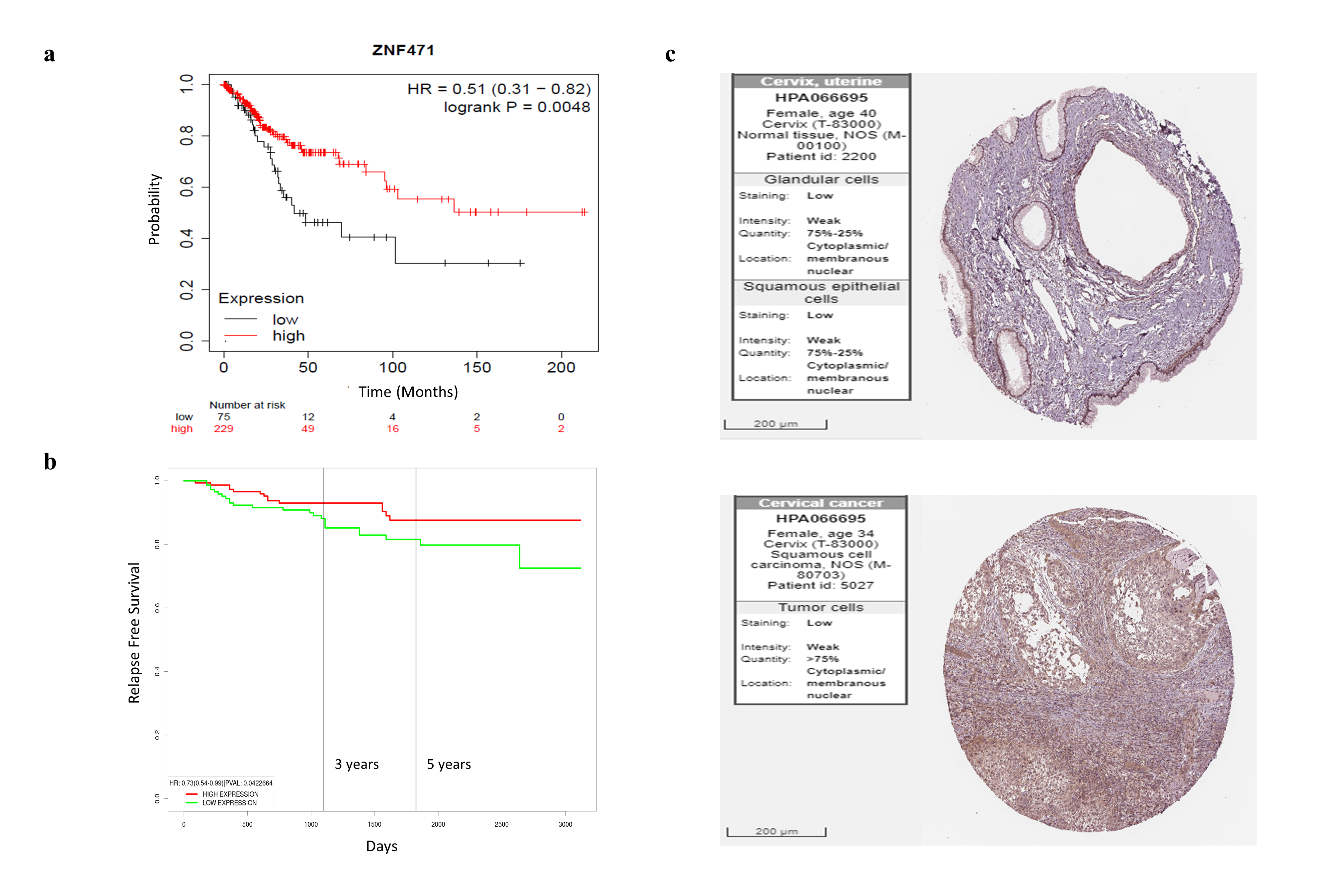

Supplement: Supplementary file 6 — High resolution image (TIF 5014 kb) [file 10565_2021_9582_MOESM3_ESM.tif]

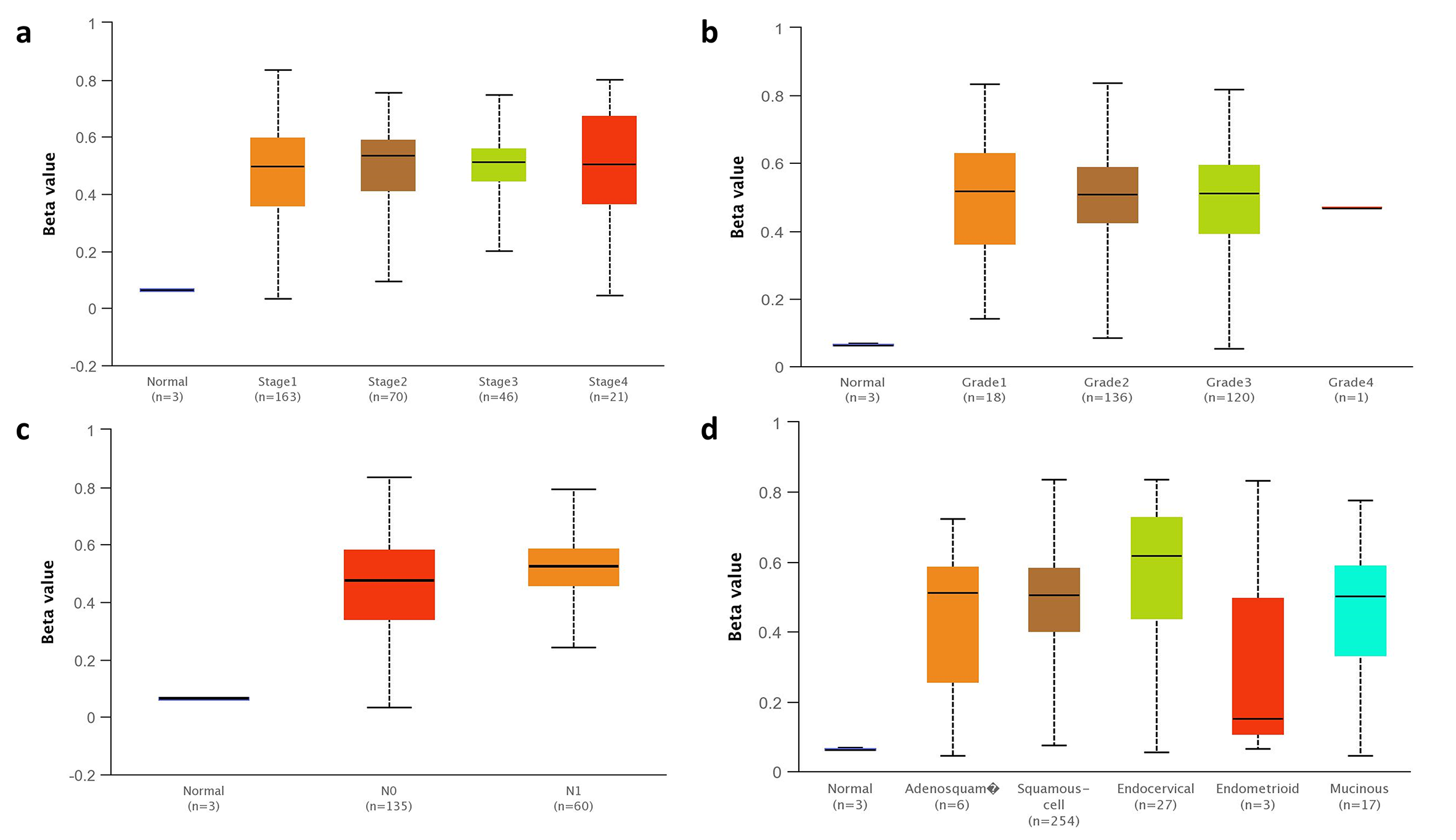

Supplement: Supplementary file 7 — (PNG 391 kb) [file 10565_2021_9582_Fig12_ESM.png]

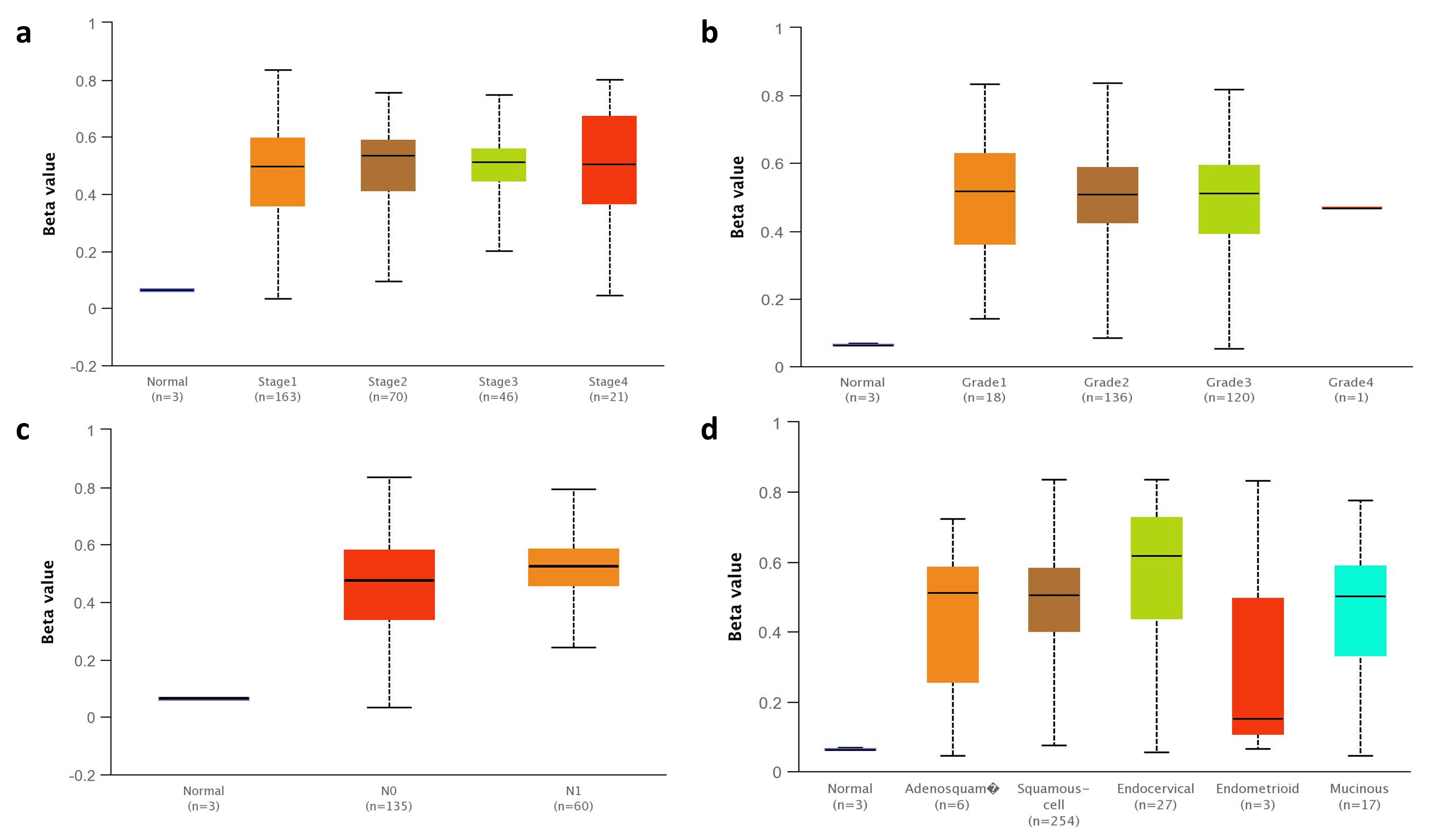

Supplement: Supplementary file 8 — High resolution image (TIF 4365 kb) [file 10565_2021_9582_MOESM4_ESM.tif]

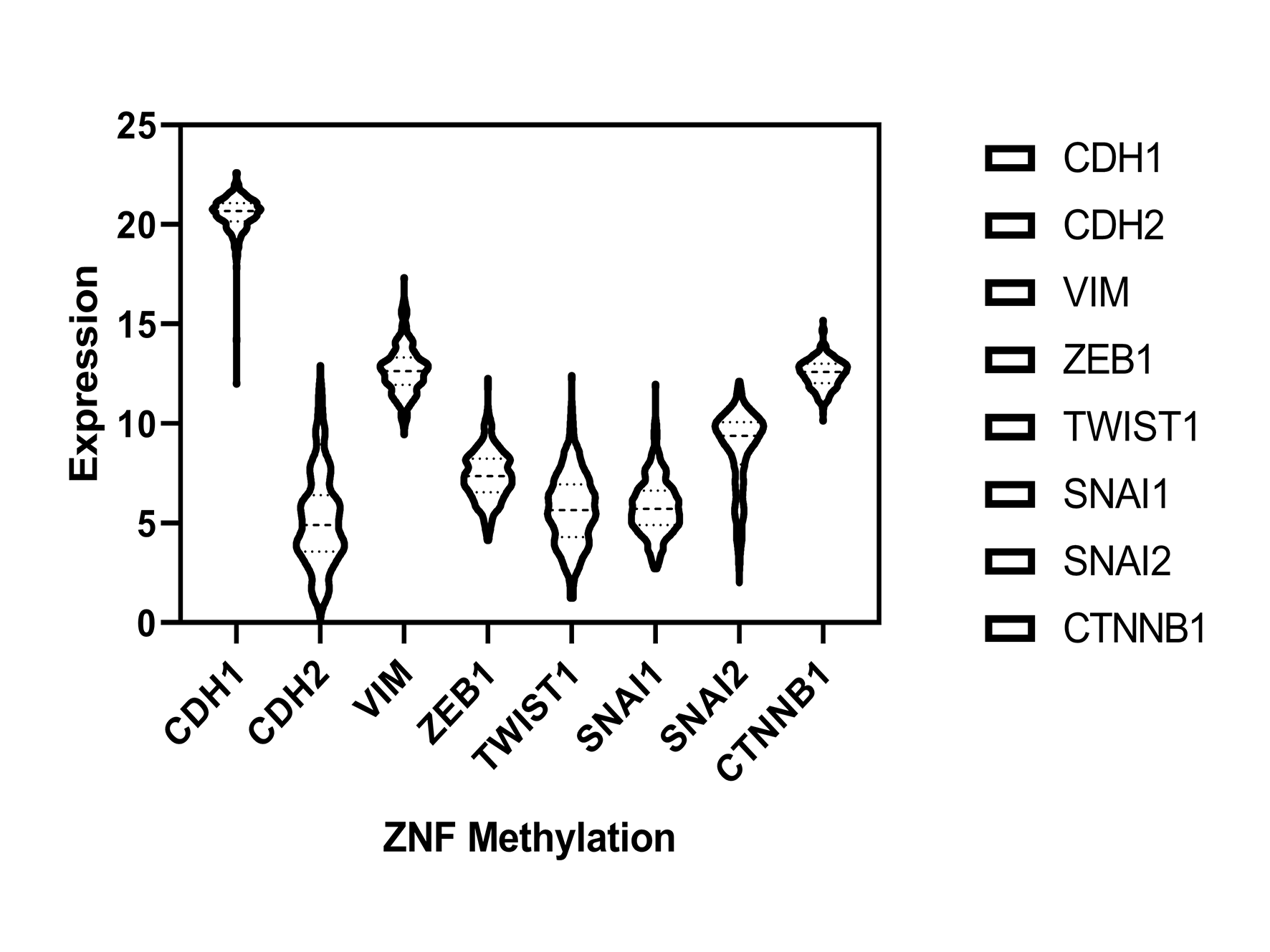

Supplement: Supplementary file 9 — (PNG 183 kb) [file 10565_2021_9582_Fig13_ESM.png]

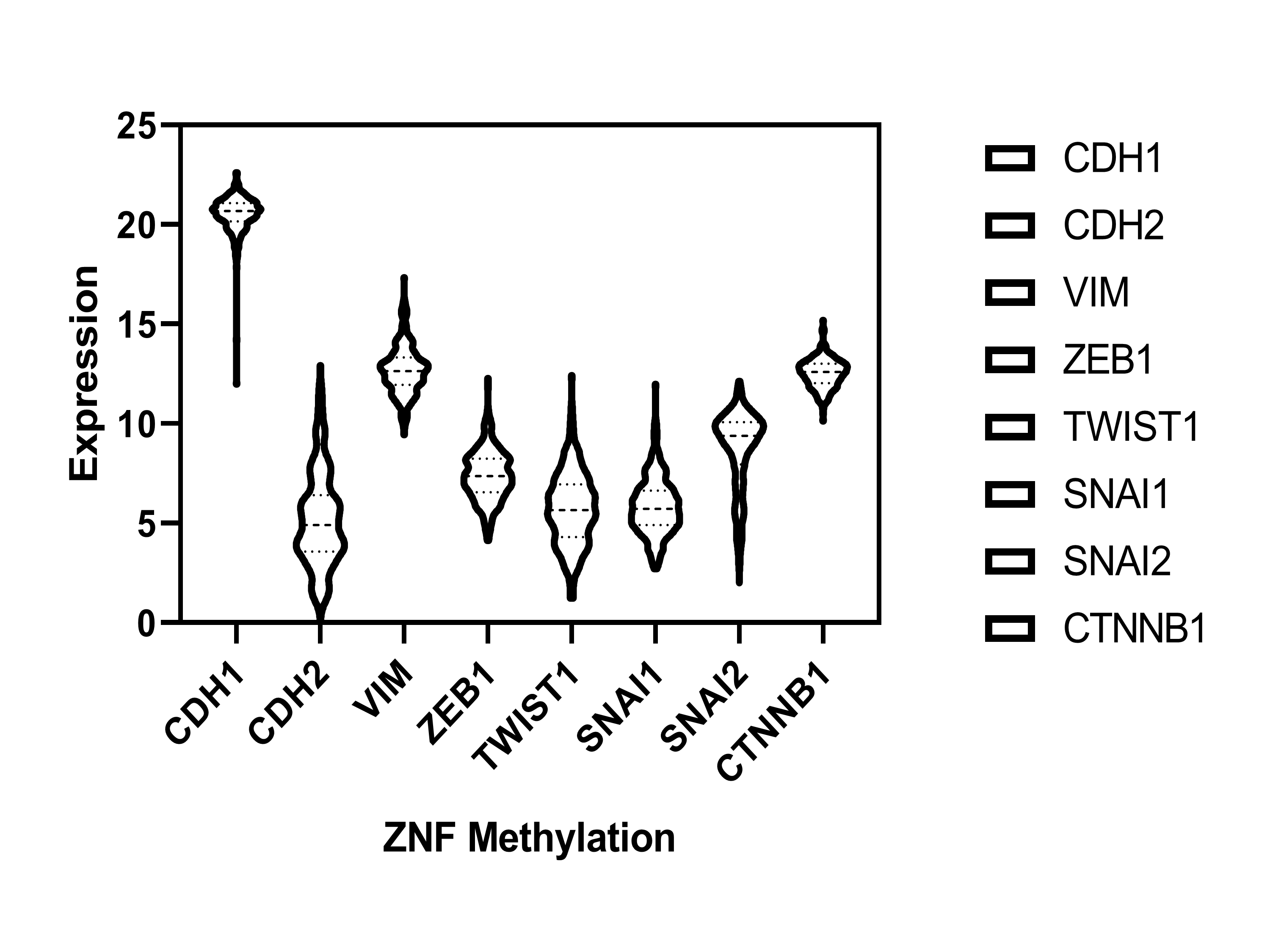

Supplement: Supplementary file 10 — High resolution image (TIF 555 kb) [file 10565_2021_9582_MOESM5_ESM.tif]

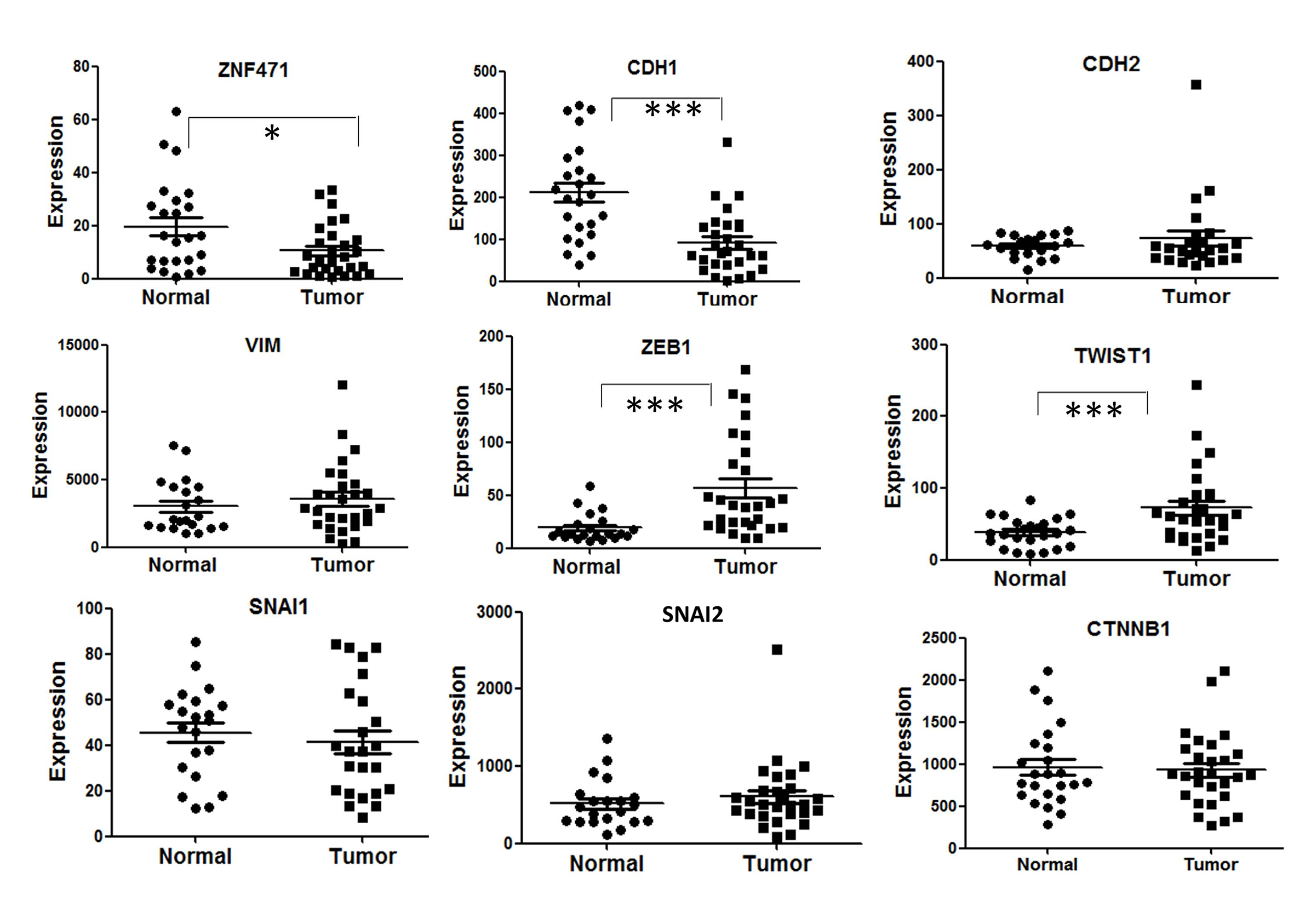

Supplement: Supplementary file 11 — (PNG 1031 kb) [file 10565_2021_9582_Fig14_ESM.png]

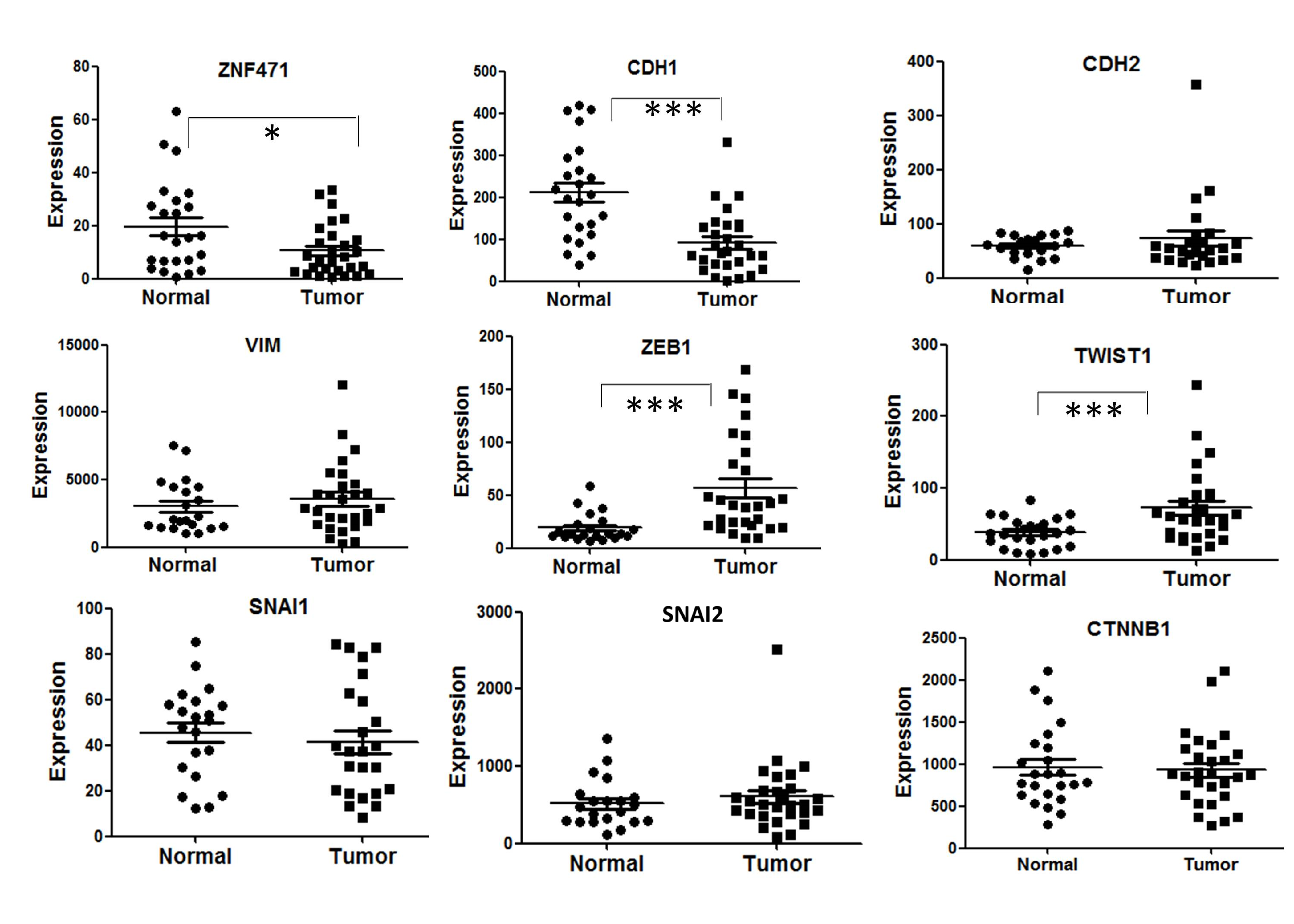

Supplement: Supplementary file 12 — High resolution image (TIF 3726 kb) [file 10565_2021_9582_MOESM6_ESM.tif]
