# Supplementary material for: ZNF471 modulates EMT and functions as methylation regulated tumor suppressor with diagnostic and prognostic significance in cervical cancer
Source: Cell Biol Toxicol. 2021 Feb 10;37(5):731–49. doi: 10.1007/s10565-021-09582-4 (PMC8490246; doi:10.1007/s10565-021-09582-4)
Supplement: Supplementary file 13 — (DOCX 27 kb) [file 10565_2021_9582_MOESM7_ESM.docx]

**Cell Line authentication Using GenePrint-10 System, Promega (STR Profiling)**

Cell lines with ≥80% match are considered to be related; derived from a common ancestry. Cell lines with between a 55% to 80% match require further analysis for authentication of relatedness.

| **Cell line** | **Loci** | **Allele (Repeat)** | **% Match** | **ATCC No.** | **Designation** |
| --- | --- | --- | --- | --- | --- |
| **HCT 15** | TH01 | 9.3 | 88 | CCL 225 | HCT15  Colorectal carcinoma, Human |
|  | D21S11 | 29,32.2 |  |  |  |
|  | D5S818 | 13 |  |  |  |
|  | D13S317 | 11 |  |  |  |
|  | D7S820 | 10,12 |  |  |  |
|  | D16S539 | 12,13 |  |  |  |
|  | AMEL | X, Y |  |  |  |
|  | v WA | 18,19 |  |  |  |
|  | TPOX | 8,11 |  |  |  |
|  | CSFIP0 | 12 |  |  |  |
|  | | | | | |
| **Cal27** | TH01 | 6,9.3 | 100 | CRL 2095 | Cal27  Tongue Carcinoma, Human |
|  | D21S11 | 28,29 |  |  |  |
|  | D5S818 | 11,12 |  |  |  |
|  | D13S317 | 10,11 |  |  |  |
|  | D7S820 | 9,10 |  |  |  |
|  | D16S539 | 11,12 |  |  |  |
|  | AMEL | X |  |  |  |
|  | v WA | 14,17 |  |  |  |
|  | TPOX | 8 |  |  |  |
|  | CSFIP0 | 10,12 |  |  |  |
|  | | | | | |
| **Jurkat** | TH01 | 6,9.3 | 100 | T1B152 | Jurkat, CloneE6, 1 Acute T cell Leukemia, Human |
|  | D21S11 | 30.2,33.2 |  |  |  |
|  | D5S818 | 9 |  |  |  |
|  | D13S317 | 8,12 |  |  |  |
|  | D7S820 | 8,12 |  |  |  |
|  | D16S539 | 11,12 |  |  |  |
|  | AMEL | X, Y |  |  |  |
|  | v WA | 17,18,19 |  |  |  |
|  | TPOX | 8,10 |  |  |  |
|  | CSFIP0 | 11,12 |  |  |  |
|  | | | | | |
| **SCC4** | TH01 | 9.3 | 100 | CRL 1624 | SCC4  Tongue carcinoma Human |
|  | D21S11 | 32.2 |  |  |  |
|  | D5S818 | 13 |  |  |  |
|  | D13S317 | 11,13 |  |  |  |
|  | D7S820 | 9,11 |  |  |  |
|  | D16S539 | 12 |  |  |  |
|  | AMEL | X, Y |  |  |  |
|  | v WA | 15,17 |  |  |  |
|  | TPOX | 8 |  |  |  |
|  | CSFIP0 | 11 |  |  |  |
|  | | | | | |
| **HT29** | TH01 | 6,9 | 100 | HTB 38 | HT - 29  Colon Adenocarcinoma Human |
|  | D21S11 | 29,30 |  |  |  |
|  | D5S818 | 11,12 |  |  |  |
|  | D13S317 | 11,12 |  |  |  |
|  | D7S820 | 10 |  |  |  |
|  | D16S539 | 11,12 |  |  |  |
|  | AMEL | X |  |  |  |
|  | v WA | 17,19 |  |  |  |
|  | TPOX | 8,9 |  |  |  |
|  | CSFIP0 | 11,12 |  |  |  |
|  | | | | | |
| **SiHa** | TH01 | 6,9 | 100 | HTB - 35 | SiHa  Cervical carcinoma  Human |
|  | D21S11 | 29,31 |  |  |  |
|  | D5S818 | 9 |  |  |  |
|  | D13S317 | 11 |  |  |  |
|  | D7S820 | 10 |  |  |  |
|  | D16S539 | 12 |  |  |  |
|  | AMEL | X |  |  |  |
|  | v WA | 14,17 |  |  |  |
|  | TPOX | 8 |  |  |  |
|  | CSFIP0 | 12 |  |  |  |
|  | | | | | |
| **CaSki** | TH01 | 7, OL | 100 | CRL - 1550 | CaSki  Cervical Carcinoma  Human |
|  | D21S11 | 30, OL |  |  |  |
|  | D5S818 | 11,13,16 |  |  |  |
|  | D13S317 | 8,12 |  |  |  |
|  | D7S820 | 8,11 |  |  |  |
|  | D16S539 | 8,11, 12, OL |  |  |  |
|  | AMEL | X |  |  |  |
|  | v WA | 14,16,17, OL |  |  |  |
|  | TPOX | 8, OL |  |  |  |
|  | CSFIP0 | 9,10 |  |  |  |
|  | | | | | |
| **MCF7** | TH01 | 6 | 100 | HTB - 22 | MCF7  Breast Adenocarcinoma |
|  | D21S11 | 30 |  |  |  |
|  | D5S818 | 11, 12, OL |  |  |  |
|  | D13S317 | 11 |  |  |  |
|  | D7S820 | 8,9 |  |  |  |
|  | D16S539 | 11,12 |  |  |  |
|  | AMEL | X |  |  |  |
|  | v WA | 14,15 |  |  |  |
|  | TPOX | 9,12 |  |  |  |
|  | CSFIP0 | 10 |  |  |  |
|  | | | | | |
| **MDAMB 231** | TH01 | 7,9.3 | 86 | HTB - 26 | MDAMB - 231  Adenocarcinoma  Human |
|  | D21S11 | 33.2 |  |  |  |
|  | D5S818 | 12 |  |  |  |
|  | D13S317 | 13 |  |  |  |
|  | D7S820 | 8,9 |  |  |  |
|  | D16S539 | 12 |  |  |  |
|  | AMEL | X |  |  |  |
|  | v WA | 15,18 |  |  |  |
|  | TPOX | 8,9 |  |  |  |
|  | CSFIP0 | 12,13 |  |  |  |
|  | | | | | |
| **HeLa** | TH01 | 4, 7 | 93 | CCL-2.2 | HeLa  S3Cervical Adenocarcinoma Human |
|  | D21S11 | 27,28 |  |  |  |
|  | D5S818 | 11,12 |  |  |  |
|  | D13S317 | OL |  |  |  |
|  | D7S820 | 8, 12 |  |  |  |
|  | D16S539 | 9, 10 |  |  |  |
|  | AMEL | X |  |  |  |
|  | v WA | 16,18 |  |  |  |
|  | TPOX | 8,12 |  |  |  |
|  | CSFIP0 | 9,10 |  |  |  |
|  | | | | | |
| **HepG2** | TH01 | 9 | 80 | HB-8065 | HepG2  Hepatocellular Carcinoma Human |
|  | D21S11 | 29,31 |  |  |  |
|  | D5S818 | 11,12 |  |  |  |
|  | D13S317 | 9,13 |  |  |  |
|  | D7S820 | 10 |  |  |  |
|  | D16S539 | 12 |  |  |  |
|  | AMEL | X, Y |  |  |  |
|  | v WA | 17 |  |  |  |
|  | TPOX | 8,9 |  |  |  |
|  | CSFIP0 | 10,11 |  |  |  |
|  | | | | | |
