# Supplementary material for: ZNF471 modulates EMT and functions as methylation regulated tumor suppressor with diagnostic and prognostic significance in cervical cancer
Source: Cell Biol Toxicol. 2021 Feb 10;37(5):731–49. doi: 10.1007/s10565-021-09582-4 (PMC8490246; doi:10.1007/s10565-021-09582-4)
Supplement: Supplementary file 14 — (DOCX 27 kb) [file 10565_2021_9582_MOESM8_ESM.docx]

| **Supplementary Table 1: DNA Methylation analysis of ZNF471 in cervical samples (Our data)** | | | | | |  |  |
| --- | --- | --- | --- | --- | --- | --- | --- |
| **Samples** | **Tissue** | **Age** | **HPV Status** | **ZNF471 Methylation (%)** | **P value** | **% ZNF471 Methylation** | **P value** |
|  |  |  |  | **(no. sites)** |  | **(Mean Methylation)** |  |
| **CL1** | SiHa |  | HPV16 | 82 |  | 77.83 |  |
| **CL2** | CaSki |  | HPV16 | 100 |  | 51.57 |  |
| **CL3** | HeLa |  | HPV18 | 68 |  | 99.11 |  |
|  |  |  |  |  |  |  |  |
| **N1** | Normal | 56 | N | 40.1 |  | 21.22 |  |
| **N2** | Normal | 45 | HPV16 | 4.5 |  | 2.59 |  |
| **N3** | Normal | 46 | N | 27.2 |  | 15.30 |  |
| **N4** | Normal | 52 | N | 31.2 |  | 15.99 |  |
| **N5** | Normal | 38 | HPV16 | 4.5 |  | 2.95 |  |
| **N6** | Normal | 46 | N | 31.2 |  | 17.48 |  |
| **N7** | Normal | 55 | N | 22.7 |  | 7.67 |  |
| **N8** | Normal | 56 | N | 18.2 |  | 7.18 |  |
| **N9** | Normal | 62 | N | 82 |  | 41.48 |  |
| **N10** | Normal | 45 | N | 55 |  | 19.24 |  |
| **N11** | Normal | 42 | N | 23 |  | 7.37 |  |
| **N12** | Normal | 51 | N | 36 |  | 13.06 |  |
| **N13** | Normal | 35 | N | 72 |  | 27.63 |  |
| **N14** | Normal | 45 | N | 0 |  | 0 |  |
| **N15** | Normal | 42 | N | 36 |  | 15.41 |  |
| **N16** | Normal | 55 | N | 23 |  | 10.28 |  |
| **N17** | Normal | 60 | N | 0 |  | 0 |  |
| **N18** | Normal | 53 | N | 0 |  | 0 |  |
| **N19** | Normal | 58 | N | 0 |  | 0 |  |
| **N20** | Normal | 61 | N | 0 |  | 0 |  |
|  |  |  |  | 29.80 ± 5.645 |  | 13.23 ± 2.59 |  |
|  |  |  |  |  |  |  |  |
| **P1** | HSIL | 65 | HPV18 | 27 |  | 13.62 |  |
| **P2** | HSIL | 55 | N | 46 |  | 21.59 |  |
| **P3** | HSIL | 40 | HPV16 | 64 |  | 27.99 |  |
| **P4** | HSIL | 74 | HPV16 | 9 |  | 2.41 |  |
| **P5** | HSIL | 74 | HPV16 | 46 |  | 16.67 |  |
| **P6** | HSIL | 53 | N | 58 |  | 16.67 |  |
| **P7** | HSIL | 53 | N | 16 |  | 7.28 |  |
| **P8** | LSIL | 20 | N | 41 |  | 17.53 |  |
| **P9** | LSIL | 54 | N | 87 |  | 53.02 |  |
| **P10** | LSIL | 46 | HPV16 | 22.7 |  | 14.79 |  |
| **P11** | LSIL | 53 | N | 27.2 |  | 12.32 |  |
| **P12** | LSIL | 46 | HPV16 | 5 |  | 1.83 |  |
| **P13** | LSIL | 40 | HPV16 | 82 |  | 36.35 |  |
| **P14** | LSIL | 32 | HPV6 | 64 |  | 31.87 |  |
| **P15** | HSIL | 55 | HPV16 | 50 |  | 24.61 |  |
| **P16** | HSIL | 28 | HPV16 | 22.7 |  | 9.35 |  |
| **P17** | HSIL | 36 | HPV16 | 64 |  | 22.89 |  |
| **P18** | HSIL | 41 | N | 11 |  | 3.42 |  |
| **P19** | HSIL | 53 | N | 13 |  | 4.85 |  |
| **P20** | HSIL | 49 | N | 9 |  | 2.85 |  |
|  |  |  |  | 43.04 ± 5.982 | N vs P: 0.1174 | 19.46 ± 3.13 | N vs P: 0.1345 |
|  |  |  |  |  |  |  |  |
| **T1** | LC-NK-SCC | 55 | HPV16 | 100 |  | 94.82 |  |
| **T2** | LC-K-SCC | 58 | HPV16 | 100 |  | 67.55 |  |
| **T3** | WD-SCC | 60 | HPV18 | 100 |  | 97.73 |  |
| **T4** | PD-SCC | 68 | N | 68 |  | 53.74 |  |
| **T5** | PD-SCC | 68 | HPV16 | 5 |  | 3.13 |  |
| **T6** | PD-SCC | 53 | HPV16 | 41 |  | 22.74 |  |
| **T7** | LC-NK-SCC | 63 | HPV16, HPV11 | 100 |  | 61.84 |  |
| **T8** | UD-SCC | 37 | HPV18 | 90 |  | 49.15 |  |
| **T9** | LC-NK-SCC | 45 | HPV16 | 90 |  | 74.76 |  |
| **T10** | UD-SCC | 71 | HPV16 | 90 |  | 66.25 |  |
| **T11** | LC-NK-SCC | 40 | HPV16 | 86 |  | 48.98 |  |
| **T12** | MD-SCC | 70 | HPV16 | 9 |  | 5.26 |  |
| **T13** | LC-NK-SCC | 50 | HPV16 | 86 |  | 78.40 |  |
| **T14** | UD-SCC | 50 | HPV16 | 81.8 |  | 78.72 |  |
| **T15** | MD-SCC | 55 | N | 72 |  | 59.32 |  |
| **T16** | MD-SCC | 67 | HPV18, HPV16 | 38 |  | 22.34 |  |
| **T17** | PD-SCC | 71 | HPV45 | 65 |  | 51.77 |  |
| **T18** | LC-K-SCC | 46 | HPV18 | 84 |  | 72.27 |  |
| **T19** | UD-SCC | 55 | N | 46 |  | 28.98 |  |
| **T20** | MD-SCC | 60 | HPV16 | 95.4 |  | 87.4 |  |
|  |  |  |  | 75.89 ± 7.729 | N vs P: 0.0019; | 59.36 ± 7.154 | N vs P: < 0.0001; |
|  |  |  |  |  | N vs T:< 0.0001 |  | N vs T:< 0.0001 |
| N: Normal, P: Premalignant, T: Tumor | | | | |  |  |  |
| PD-SCC: Poorly differentiated squamous cell carcinoma, LC –NK-SCC: Large cell non-keratinizing squamous cell carcinoma, LC –K-SCC: Large cell keratinizing squamous cell carcinoma, WD-SCC: Well differentiated squamous cell carcinoma, UD-SCC: Undifferentiated squamous cell carcinoma, MD-SCC: Moderately differentiated squamous cell carcinoma. | | | | | | | |
| N vs. P: Normal vs Premalignant, N vs. T: Normal vs. Tumor, P vs. T: Premalignant vs. Tumor | | | | | | |  |
