# Supplementary material for: ZNF471 modulates EMT and functions as methylation regulated tumor suppressor with diagnostic and prognostic significance in cervical cancer
Source: Cell Biol Toxicol. 2021 Feb 10;37(5):731–49. doi: 10.1007/s10565-021-09582-4 (PMC8490246; doi:10.1007/s10565-021-09582-4)
Supplement: Supplementary file 15 — (DOCX 15 kb) [file 10565_2021_9582_MOESM9_ESM.docx]

| **Supplementary Table 2: Number of data sets and samples analyzed** | | | | | |
| --- | --- | --- | --- | --- | --- |
|  | **Normal** | **SIL** | **Tumor** | **Cell Lines** | **Total Samples** |
| **Our data** | 17 | 17 | 14 | 3 | 51 |
| **E-GEOD-30760** | 153 |  | 62 |  | 215 |
| **E-GEOD-30759** | 15 |  | 48 |  | 63 |
| **E-GEOD-41384** | 3 | 13 | 3 |  | 19 |
| **E-GEOD-46306** | 20 | 18 | 6 |  | 44 |
| **TCGA (CESC)** | 3 |  | 306 |  | 309 |
| **Total Samples** | 211 | 48 | 439 | 3 | 701 |
