# Supplementary material for: ZNF471 modulates EMT and functions as methylation regulated tumor suppressor with diagnostic and prognostic significance in cervical cancer
Source: Cell Biol Toxicol. 2021 Feb 10;37(5):731–49. doi: 10.1007/s10565-021-09582-4 (PMC8490246; doi:10.1007/s10565-021-09582-4)
Supplement: Supplementary file 16 — (DOCX 52 kb) [file 10565_2021_9582_MOESM10_ESM.docx]

| **Supplementary Table 3: Clinico-pathological characteristics of TCGA (CESC) dataset** | | | | | | | | |  |  |  |
| --- | --- | --- | --- | --- | --- | --- | --- | --- | --- | --- | --- |
|  | **Sample** | **Down regulated** | **%** | **Upregulated** | **%** | **Not Altered** | **%** | **Methylation** | **%** | **Unmethylated** | **%** |
| **Smoking** |  |  |  |  |  |  |  |  |  |  |  |
| **Current reformed smoker for < or = 15 years** | 21 | 14 | 66.66667 | 1 | 4.761905 | 6 | 28.57143 | 11 | 52.38095 | 10 | 47.61905 |
| **Current reformed smoker for > 15 years** | 7 | 4 | 57.14286 | 1 | 14.28571 | 2 | 28.57143 | 4 | 57.14286 | 3 | 42.85714 |
| **Current smoker** | 29 | 20 | 68.96552 | 4 | 13.7931 | 5 | 17.24138 | 15 | 51.72414 | 14 | 48.27586 |
| **Lifelong Non-smoker** | 69 | 53 | 76.81159 | 5 | 7.246377 | 11 | 15.94203 | 38 | 55.07246 | 31 | 44.92754 |
| **Total** | 126 | 91 | 72.22222 | 11 | 8.730159 | 24 | 19.04762 | 68 | 53.96825 | 58 | 46.03175 |
| **No of Pregnancy** |  |  |  |  |  |  |  |  |  |  |  |
| **0** | 8 | 5 | 62.5 | 0 | 0 | 3 | 37.5 | 4 | 50 | 4 | 50 |
| **1** | 18 | 13 | 72.22222 | 1 | 5.555556 | 4 | 22.22222 | 12 | 66.66667 | 6 | 33.33333 |
| **2** | 29 | 19 | 65.51724 | 6 | 20.68966 | 4 | 13.7931 | 16 | 55.17241 | 13 | 44.82759 |
| **3** | 26 | 16 | 61.53846 | 1 | 3.846154 | 9 | 34.61538 | 11 | 42.30769 | 15 | 57.69231 |
| **4** | 20 | 14 | 70 | 2 | 10 | 4 | 20 | 9 | 45 | 5 | 25 |
| **5 and above** | 28 | 22 | 78.57143 | 2 | 7.142857 | 4 | 14.28571 | 16 | 57.14286 | 12 | 42.85714 |
| **Total** | 129 | 89 | 68.99225 | 12 | 9.302326 | 28 | 21.70543 | 68 | 52.71318 | 55 | 42.63566 |
| **Vital Status** |  |  |  |  |  |  |  |  |  |  |  |
| **Living** | 129 | 92 | 71.31783 | 37 | 28.68217 |  | 0 | 71 | 55.03876 | 58 | 44.96124 |
| **Decreased** | 23 | 18 | 78.26087 | 5 | 21.73913 | 4 | 17.3913 | 20 | 86.95652 | 3 | 13.04348 |
| **Total** | 152 | 110 | 72.36842 | 42 | 27.63158 | 4 | 2.631579 | 91 | 59.86842 | 61 | 40.13158 |
| **Response** |  |  |  |  |  |  |  |  |  |  |  |
| **Complete Response** | 32 | 23 | 71.875 | 5 | 15.625 | 4 | 12.5 | 14 | 43.75 | 18 | 56.25 |
| **Progression** | 4 | 3 | 75 | 0 | 0 | 1 | 25 | 2 | 50 | 2 | 50 |
| **Total** | 36 | 27 | 75 | 5 | 13.88889 | 5 | 13.88889 | 16 | 44.44444 | 20 | 55.55556 |
| **Stage** |  |  |  |  |  |  |  |  |  |  |  |
| **Stage I** | 3 | 3 | 100 | 0 | 0 | 0 | 0 | 2 | 66.66667 | 1 | 33.33333 |
| **Stage 1A** | 3 | 1 | 33.33333 | 1 | 33.33333 | 1 | 33.33333 | 2 | 66.66667 | 1 | 33.33333 |
| **Stage I B** | 24 | 21 | 87.5 | 1 | 4.166667 | 2 | 8.333333 | 12 | 50 | 12 | 50 |
| **Stage IB1** | 40 | 31 | 77.5 | 4 | 10 | 5 | 12.5 | 28 | 70 | 12 | 30 |
| **Stage IB2** | 21 | 12 | 57.14286 | 2 | 9.52381 | 7 | 33.33333 | 12 | 57.14286 | 9 | 42.85714 |
| **Stage II** | 3 | 3 | 100 | 0 | 0 | 0 | 0 | 1 | 33.33333 | 2 | 66.66667 |
| **Stage IIA** | 3 | 3 | 100 | 0 | 0 | 0 | 0 | 2 | 66.66667 | 1 | 33.33333 |
| **Stage IIA1** | 5 | 5 | 100 | 0 | 0 | 0 | 0 | 2 | 40 | 3 | 60 |
| **Stage IIA2** | 5 | 1 | 20 | 0 | 0 | 4 | 80 | 0 | 0 | 5 | 100 |
| **Stage IIB** | 13 | 9 | 69.23077 | 3 | 23.07692 | 2 | 15.38462 | 7 | 53.84615 | 6 | 46.15385 |
| **Stage IIIB** | 22 | 16 | 72.72727 | 0 | 0 | 6 | 27.27273 | 14 | 63.63636 | 8 | 36.36364 |
| **Stage IVA** | 7 | 2 | 28.57143 | 2 | 28.57143 | 3 | 42.85714 | 2 | 28.57143 | 5 | 71.42857 |
| **Total** | 149 | 107 | 71.81208 | 13 | 8.724832 | 30 | 20.13423 | 84 | 56.37584 | 65 | 43.62416 |
| **Metastasis** |  |  |  |  |  |  |  |  |  |  |  |
| **M0** | 64 | 47 | 73.4375 | 3 | 4.6875 | 14 | 21.875 | 34 | 53.125 | 30 | 46.875 |
| **M1** | 4 | 0 | 0 | 2 | 50 | 2 | 50 | 0 | 0 | 4 | 100 |
| **MX** | 53 | 38 | 71.69811 | 8 | 15.09434 | 7 | 13.20755 | 27 | 50.9434 | 22 | 41.50943 |
| **Total** | 121 | 85 | 70.24793 | 13 | 10.7438 | 23 | 19.00826 | 61 | 50.41322 | 56 | 46.28099 |
|  |  |  |  |  |  |  |  |  |  |  |  |
| **N0** | 76 | 50 | 65.78947 | 9 | 11.84211 | 17 | 22.36842 | 33 | 43.42105 | 37 | 48.68421 |
| **N1** | 36 | 27 | 75 | 2 | 5.555556 | 6 | 16.66667 | 21 | 58.33333 | 15 | 41.66667 |
| **Nx** | 12 | 10 | 83.33333 | 1 | 8.333333 | 1 | 8.333333 | 7 | 58.33333 | 5 | 41.66667 |
| **Total** | 124 | 87 | 70.16129 | 12 | 9.677419 | 24 | 19.35484 | 61 | 49.19355 | 57 | 45.96774 |
|  |  |  |  |  |  |  |  |  |  |  |  |
| **T1a** | 3 | 1 | 33.33333 | 1 | 33.33333 | 1 | 33.33333 | 1 | 33.33333 | 2 | 66.66667 |
| **T1b** | 16 | 13 | 81.25 | 1 | 6.25 | 2 | 12.5 | 8 | 50 | 8 | 50 |
| **T1b1** | 46 | 35 | 76.08696 | 4 | 8.695652 | 7 | 15.21739 | 31 | 67.3913 | 15 | 32.6087 |
| **T1b2** | 18 | 11 | 61.11111 | 2 | 11.11111 | 5 | 27.77778 | 9 | 50 | 9 | 50 |
| **T2** | 3 | 2 | 66.66667 | 0 | 0 | 1 | 33.33333 | 2 | 66.66667 | 1 | 33.33333 |
| **T2a** | 5 | 3 | 60 | 2 | 40 | 0 | 0 | 1 | 20 | 4 | 80 |
| **T2a1** | 6 | 6 | 100 | 0 | 0 | 0 | 0 | 3 | 50 | 3 | 50 |
| **T2a2** | 7 | 4 | 57.14286 | 0 | 0 | 3 | 42.85714 | 2 | 28.57143 | 5 | 71.42857 |
| **T2b** | 10 | 3 | 30 | 2 | 20 | 5 | 50 | 4 | 40 | 6 | 60 |
| **Tx** | 7 | 7 | 100 | 0 | 0 | 0 | 0 | 5 | 71.42857 | 2 | 28.57143 |
| **Total** | 121 | 85 | 70.24793 | 12 | 9.917355 | 24 | 19.83471 | 66 | 54.54545 | 55 | 45.45455 |
| **Overall Survival** |  |  |  |  |  |  |  |  |  |  |  |
| **0-6 months** | 77 | 54 | 70.12987 | 8 | 10.38961 | 15 | 19.48052 | 38 | 49.35065 | 36 | 46.75325 |
| **7-12 months** | 9 | 7 | 77.77778 | 0 | 0 | 2 | 22.22222 | 7 | 77.77778 | 2 | 22.22222 |
| **13-18 months** | 8 | 7 | 87.5 | 0 | 0 | 1 | 12.5 | 4 | 50 | 4 | 50 |
| **18-24 months** | 6 | 5 | 83.33333 | 0 | 0 | 1 | 16.66667 | 3 | 50 | 3 | 50 |
| **24-30 months** | 7 | 6 | 85.71429 | 0 | 0 | 1 | 14.28571 | 6 | 85.71429 | 1 | 14.28571 |
| **30-36 months** | 3 | 3 | 100 | 0 | 0 | 0 | 0 | 3 | 100 | 0 | 0 |
| **36-42 months** | 6 | 3 | 50 | 1 | 16.66667 | 2 | 33.33333 | 2 | 33.33333 | 4 | 66.66667 |
| **42-48 month and above** | 36 | 23 | 63.88889 | 4 | 11.11111 | 8 | 22.22222 | 16 | 44.44444 | 20 | 55.55556 |
| **Total** | 152 | 108 | 71.05 | 13 | 8.552632 | 30 | 19.73684 | 79 | 51.97368 | 70 | 46.05263 |
| **Vital status** |  |  |  |  |  |  |  |  |  |  |  |
| **Decreased** | 23 | 18 | 78.26087 | 2 | 8.695652 | 3 | 13.04348 | 15 | 65.21739 | 8 | 34.78261 |
| **Living** | 129 | 92 | 71.31783 | 11 | 8.527132 | 26 | 20.15504 | 40 | 31.00775 | 52 | 40.31008 |
| **Total** | 152 | 110 | 72.36842 | 13 | 8.552632 | 29 | 19.07895 | 55 | 36.18421 | 60 | 39.47368 |
| **Recurrence** |  |  |  |  |  |  |  |  |  |  |  |
| **Yes** | 57 |  |  |  |  |  |  |  | 0 |  | 0 |
| **No** | 131 |  |  |  |  |  |  |  | 0 |  | 0 |
| **Total** |  |  |  |  |  |  |  |  |  |  |  |
| **Keratinizing squamous cell carcinoma** | 32 | 24 | 75 | 1 | 3.125 | 7 | 21.875 | 20 | 62.5 | 12 | 37.5 |
| **Non-keratinizing squamous cell carcinoma** | 65 | 47 | 72.30769 | 6 | 9.230769 | 11 | 16.92308 | 33 | 50.76923 | 32 | 49.23077 |
| **Total** | 97 | 71 | 73.19588 | 7 | 7.216495 | 18 | 18.5567 | 53 | 54.63918 | 44 | 45.36082 |
| **Menopausal State** |  |  |  |  |  |  |  |  |  |  |  |
| **Peri** | 9 | 6 | 66.66667 | 0 | 0 | 3 | 33.33333 | 4 | 44.44444 | 5 | 55.55556 |
| **Post** | 48 | 30 | 62.5 | 5 | 10.41667 | 13 | 27.08333 | 25 | 52.08333 | 23 | 47.91667 |
| **Pre** | 68 | 54 | 79.41176 | 5 | 7.352941 | 9 | 13.23529 | 39 | 57.35294 | 29 | 42.64706 |
| **Total** | 125 | 90 | 72 | 10 | 8 | 25 | 20 | 68 | 54.4 | 57 | 45.6 |
| **Histology** |  |  |  |  |  |  |  |  |  |  |  |
| **G1** | 14 | 9 | 64.28571 | 0 | 0 | 5 | 35.71429 | 6 | 42.85714 | 8 | 57.14286 |
| **G2** | 70 | 50 | 71.42857 | 7 | 10 | 13 | 18.57143 | 39 | 55.71429 | 31 | 44.28571 |
| **G3** | 62 | 45 | 72.58065 | 6 | 9.677419 | 11 | 17.74194 | 38 | 61.29032 | 24 | 38.70968 |
| **Gx** | 5 | 5 | 100 | 0 | 0 | 0 | 0 | 2 | 40 | 3 | 60 |
| **Total** | 151 | 109 | 72.18543 | 13 | 8.609272 | 29 | 19.2053 | 85 | 56.29139 | 66 | 43.70861 |
|  |  |  |  |  |  |  |  |  |  |  |  |
| **Cervical Squamous Cell Carcinoma** | 125 | 98 | 78.4 | 7 | 5.6 | 20 | 16 | 70 | 56 | 55 | 44 |
| **Endocervical Type of Adenocarcinoma** | 23 | 13 | 56.52174 | 5 | 21.73913 | 5 | 21.73913 | 14 | 60.86957 | 9 | 39.13043 |
| **Total** | 148 | 111 | 75 | 12 | 8.108108 | 25 | 16.89189 | 84 | 56.75676 | 64 | 43.24324 |
| **Lympho-vascular Invasion Indicator** |  |  |  |  |  |  |  |  |  |  |  |
| **ABSENT** | 49 | 33 | 67.34694 | 4 | 8.163265 | 12 | 24.4898 | 25 | 51.02041 | 24 | 48.97959 |
| **PRESENT** | 55 | 39 | 70.90909 | 8 | 14.54545 | 9 | 16.36364 | 34 | 61.81818 | 21 | 38.18182 |
| **Total** | 104 | 72 | 69.23077 | 12 | 11.53846 | 21 | 20.19231 | 59 | 56.73077 | 45 | 43.26923 |
| **Corpus Uteri Involvement Indicator** |  |  |  |  |  |  |  |  |  |  |  |
| **ABSENT** | 71 | 54 | 76.05634 | 7 | 9.859155 | 10 | 14.08451 | 43 | 60.56338 | 28 | 39.43662 |
| **PRESENT** | 12 | 7 | 58.33333 | 0 | 0 | 5 | 41.66667 | 7 | 58.33333 | 5 | 41.66667 |
| **Total** | 83 | 61 | 73.49398 | 7 | 8.433735 | 15 | 18.07229 | 50 | 60.24096 | 33 | 39.75904 |

- **Clinico-pathological characteristics of E-GEOD-41384 dataset:**

|  |  | **cg14289985** | | **cg24713204** | |
| --- | --- | --- | --- | --- | --- |
|  |  | **Unmethylated** | **Methylated** | **Unmethylated** | **Methylated** |
| **Disease Status** | Normal | 3 | 0 | 3 | 0 |
|  | CIN1 | 3 | 0 | 3 | 0 |
|  | CIN2 | 3 | 1 | 2 | 2 |
|  | CIS | 1 | 5 | 1 | 5 |
|  | Tumor | 0 | 3 | 2 | 2 |
| **HPV Status** | Positive | 16 | 1 | 11 | 8 |
|  | Not Determined | 0 | 2 | 1 | 1 |
| **Total** | 19 | | | | |

- **Clinico-pathological characteristics of E-GEOD-46306 dataset:**

|  |  | **cg14289985** | | **cg24713204** | |
| --- | --- | --- | --- | --- | --- |
|  |  | **Unmethylated** | **Methylated** | **Unmethylated** | **Methylated** |
| **Disease Status** | Normal | 20 | 0 | 18 | 2 |
|  | CIN3 | 15 | 0 | 12 | 3 |
|  | Tumor | 6 | 3 | 4 | 5 |
| **HPV Status** | Positive | 21 | 3 | 16 | 8 |
|  | Negative | 20 | 0 | 18 | 2 |
| **Total** | 44 | | | | |

- **Clinico-pathological characteristics of E-GEOD-30759 dataset:**

|  |  | **cg14289985** | | **cg24713204** | |
| --- | --- | --- | --- | --- | --- |
|  |  | **Unmethylated** | **Methylated** | **Unmethylated** | **Methylated** |
| **Disease Status** | Normal | 15 | 0 | 15 | 0 |
|  | Tumor | 22 | 26 | 8 | 40 |
| **Stage** | NA | 15 | 1 | 15 | 1 |
|  | 1 | 16 | 10 | 3 | 23 |
|  | 2 | 5 | 5 | 1 | 9 |
|  | 3 | 4 | 7 | 4 | 7 |
| **Death Status** | NA | 15 | 0 | 15 | 0 |
|  | Not specified | 5 | 18 | 0 | 23 |
|  | Cancer | 16 | 9 | 8 | 17 |
| **Recurrence Free Survival (Years)** | NA | 15 | 0 | 15 | 0 |
|  | 0 to 2 | 9 | 10 | 5 | 14 |
|  | 2 to 4 | 5 | 2 | 1 | 6 |
|  | 4 to 6 | 0 | 1 | 0 | 1 |
|  | 6 to 8 | 4 | 2 | 2 | 4 |
|  | 8 to 10 & above | 3 | 11 | 0 | 14 |
| **Age (Years)** | 30 - 40 | 7 | 2 | 5 | 4 |
|  | 40 - 50 | 12 | 7 | 10 | 9 |
|  | 50 - 60 | 14 | 5 | 12 | 7 |
|  | 60 & above | 11 | 5 | 8 | 8 |
| **Relapse** | NA | 15 | 0 | 15 | 0 |
|  | Not specified | 9 | 14 | 2 | 21 |
|  | Yes | 13 | 12 | 6 | 19 |
| **Survival Time (Years)** | NA | 15 | 0 | 15 | 0 |
|  | 0 to 2 | 6 | 1 | 3 | 4 |
|  | 2 to 4 | 7 | 8 | 3 | 12 |
|  | 4 to 6 | 0 | 1 | 0 | 1 |
|  | 6 to 8 | 4 | 2 | 1 | 5 |
|  | 8 to 10 & above | 5 | 14 | 1 | 18 |
| **Total** | 63 | | | | |

- **Clinico-pathological characteristics of E-GEOD-30760 dataset:**

|  |  | **cg14289985** | | **cg24713204** | |
| --- | --- | --- | --- | --- | --- |
|  |  | **Unmethylated** | **Methylated** | **Unmethylated** | **Methylated** |
| **Disease Status** | Normal | 152 | 0 | 151 | 1 |
|  | Tumor | 27 | 36 | 12 | 51 |
| **Stage** | NA | 152 | 0 | 151 | 1 |
|  | Not specified | 4 | 12 | 1 | 15 |
|  | 1 | 13 | 13 | 7 | 19 |
|  | 2 | 5 | 5 | 2 | 8 |
|  | 3 | 6 | 5 | 2 | 9 |
| **Recurrence Free Survival (Years)** | NA | 152 | 0 | 151 | 1 |
|  | Not specified | 3 | 13 | 1 | 15 |
|  | 0 to 2 | 12 | 7 | 6 | 13 |
|  | 2 to 4 | 4 | 3 | 1 | 6 |
|  | 4 to 6 | 1 | 0 | 1 | 0 |
|  | 6 to 8 | 5 | 1 | 2 | 4 |
|  | 8 to 10 & above | 2 | 12 | 1 | 13 |
| **Age (Years)** | Less than 20 | 42 | 0 | 42 | 0 |
|  | 20-30 | 81 | 1 | 79 | 3 |
|  | 30-40 | 57 | 10 | 54 | 13 |
|  | 40-50 | 23 | 9 | 22 | 10 |
|  | 50 & above | 13 | 16 | 4 | 25 |
| **Relapse** | NA | 152 | 0 | 151 | 1 |
|  | Not specified | 12 | 26 | 3 | 35 |
|  | Yes | 15 | 10 | 9 | 16 |
| **Survival Time (Years)** | NA | 152 | 0 | 151 | 1 |
|  | Not specified | 3 | 12 | 1 | 14 |
|  | 0 to 2 | 2 | 5 | 0 | 7 |
|  | 2 to 4 | 10 | 5 | 6 | 9 |
|  | 4 to 6 | 1 | 0 | 1 | 0 |
|  | 6 to 8 | 5 | 1 | 2 | 4 |
|  | 8 to 10 & above | 6 | 13 | 2 | 17 |
| **Total** | 215 | | | | |
