# Supplementary material for: ZNF471 modulates EMT and functions as methylation regulated tumor suppressor with diagnostic and prognostic significance in cervical cancer
Source: Cell Biol Toxicol. 2021 Feb 10;37(5):731–49. doi: 10.1007/s10565-021-09582-4 (PMC8490246; doi:10.1007/s10565-021-09582-4)
Supplement: Supplementary file 17 — (DOCX 22 kb) [file 10565_2021_9582_MOESM11_ESM.docx]

| **Supplementary Table 4: List of primers used** **in the study** |  |  |  |
| --- | --- | --- | --- |
| **Primer Name** | **Sequence (5'-3')** | **Annealing Temperature** | **Product Size** |
| **Bisulfite Genomic Sequencing (BGS)** | |  |  |
| ZNF471-F | ATTTTTATTTTGTTAAGGGGTTGAA | 57.5°C | 363bp |
| ZNF471-R | AAAAACCAAAAACRTCCCTAAT |  |  |
| **Reverse Transcriptase PCR (RT-PCR)** | |  |  |
| ZNF471-F | ATGCTTTCAAACAGAGATCACAC | 60 °C | 195bp |
| ZNF471-R | CACAGGATGAGCTATCACTAAAA |  |  |
| VIM-RT-PCR-F | ATCCAAGTTTGCTGACCTCTCTGAG | 60°C | 102bp |
| VIM-RT-PCR-R | AGGGACTGCACCTGTCTCCGGT |  |  |
| CTNNB1-F | GATATTGGTGCCCAGGGA | 60°C | 127bp |
| CTNNB1-R | CACCCATCTCATGTTCCATC |  |  |
| TW1-F | GGCTCAGCTACGCCTTCTC | 60°C | 130bp |
| TW1-R | TCCTTCTCTGGAAACAATGACA |  |  |
| TW2-F | GCAAGAAGTCGAGCGAAGAT | 57.5°C | 92bp |
| TW2-R | GCTCTGCAGCTCCTCGAA |  |  |
| SNAI1-F | TATGCTGCCTTCCCAGGCTTG | 60°C | 143bp |
| SNAI1-R | ATGTGCATCTTGAGGGCACCC |  |  |
| SNAI2-F | ATCTGCGGCAAGGCGTTTTCCA | 60°C | 127bp |
| SNAI2-R | GAGCCCTCAGATTTGACCTGTC |  |  |
| ZEB1-F | TCCTGAGGCACCTGAAGAGG | 57.5°C | 139bp |
| ZEB1-R | CAGAGAGGTAAAGCGTTTATAGCC |  |  |
| CDH1-F | GCCTCCTGAAAAGAGAGTGGAAG | 60°C | 131bp |
| CDH1-R | TGGCAGTGTCTCTCCAAATCCG |  |  |
| CDH2-F | CCTCCAGAGTTTACTGCCATGAC | 60°C | 149bp |
| CDH2-R | GTAGGATCTCCGCCACTGATTC |  |  |
| GAPDH-F | GGCTCCCTTGGGTATATGGT | 60 °C | 97bp |
| GAPDH-R | TTGATTTTGGAGGGATCTCG |  |  |
| ACTB –F | GACGACATGGAGAAAATCTG | 60°C | 132bp |
| ACTB- R | ATGATCTGGGTCATCTTCTC |  |  |
| **Promoter Construct: Luciferase Assay** | |  |  |
| ZNF471-Lu-F | CTCTGCGGTACCTGTATCCCCCTGAA | 60°C | 904bp |
| ZNF471-Lu-R | GCCTCTAAGCTTGGGAAATGTAGTCG |  |  |
| **Cloning: Retrovirus vector** | | | |
| ZNF471-F | GCCCTGGATCCCCCAAGACACTGTT | 57.5°C | 2078bp |
| ZNF471-R | TGTTCCTCGAGCTATTCGGCCATCTT |  |  |
| **ChIP-PCR** |  |  |  |
| VIM - F | AGGCAAGTCGATGGACAGAG | 60°C | 180bp |
| VIM - R | AAGTCGCGGAGAAAGAAACA |  |  |
| The sequence underlined within the table represents the restriction sites incorporated for cloning. |  |  |  |
