# Supplementary material for: ZNF471 modulates EMT and functions as methylation regulated tumor suppressor with diagnostic and prognostic significance in cervical cancer
Source: Cell Biol Toxicol. 2021 Feb 10;37(5):731–49. doi: 10.1007/s10565-021-09582-4 (PMC8490246; doi:10.1007/s10565-021-09582-4)
Supplement: Supplementary file 18 — (DOCX 15 kb) [file 10565_2021_9582_MOESM12_ESM.docx]

| **Supplementary Table 5: Pearson’s correlation between *ZNF471* mRNA expression and the methylation percentage in TCGA cervical cancer datasets (CESC)** | | | | |
| --- | --- | --- | --- | --- |
|  |  |  |  |  |
|  | **Pearson r** | **95% confidence interval** | **P value (two-tailed)** | **R squared** |
| **cg14277392** | -0.7231 | -0.7850 to -0.6469 | < 0.0001 | 0.5228 |
| **cg02823803** | -0.5907 | -0.6766 to -0.4889 | < 0.0001 | 0.3489 |
| **cg24713204** | -0.747 | -0.8041 to -0.6761 | < 0.0001 | 0.558 |
| **cg14289985** | -0.682 | -0.7517 to -0.5971 | < 0.0001 | 0.4651 |
| **cg19811761** | -0.7867 | -0.8358 to -0.7253 | < 0.0001 | 0.619 |
| **cg11539780** | -0.7495 | -0.8062 to -0.6793 | < 0.0001 | 0.5618 |
| **cg14042851** | -0.7495 | -0.8062 to -0.6793 | < 0.0001 | 0.5618 |
| **cg19358877** | -0.7333 | -0.7932 to -0.6593 | < 0.0001 | 0.5377 |
| **cg00711090** | 0.1603 | 0.01751 to 0.2966 | 0.028 | 0.02568 |
| **cg00674365** | -0.7964 | -0.8435 to -0.7371 | < 0.0001 | 0.6342 |
