# Supplementary material for: ZNF471 modulates EMT and functions as methylation regulated tumor suppressor with diagnostic and prognostic significance in cervical cancer
Source: Cell Biol Toxicol. 2021 Feb 10;37(5):731–49. doi: 10.1007/s10565-021-09582-4 (PMC8490246; doi:10.1007/s10565-021-09582-4)
Supplement: Supplementary file 19 — (DOCX 48 kb) [file 10565_2021_9582_MOESM13_ESM.docx]

| **Supplementary Table 6: Sensitivity and specificity analysis of our study.** | | | | | | | | | | |  |  |  |  |  |  |  |
| --- | --- | --- | --- | --- | --- | --- | --- | --- | --- | --- | --- | --- | --- | --- | --- | --- | --- |
|  |  |  |  |  |  |  |  |  |  | | | |  |  |  |  |  |
|  |  | **Sensitivity** | **95% CI** | **Specificity** | **95% CI** | **PPV** | **95% CI** | **NPV** | **95% CI**  **Likelihood Ratio**  **RR** | | | **95% CI** | **Odds ratio** | **95% CI** | **P value** | **AUC** | **P value** |
| CpG 1 | N vs.P | 0 | 0 | 0 | 0 | 0 | 0 | 0 | 0 | 0 | 0.8889 | 0.0187 to 42.2640 |  |  | 0.9523 | 0.5 | 1 |
|  | P vs.T | 1 | 0.8049 to 1.000 | 0.3571 | 0.1276 to 0.6486 | 0.6538 | 0.4433 to 0.8279 | 1 | 0.4782 to 1.000 | 1.556 | 13.2 | 1.007 to 407.7 | 20.26 | 1.007 to 407.7 | 0.0118 | 0.6786 | 0.09166 |
|  | N vs.T | 1 | 0.7820 to 1.000 | 0.3571 | 0.1276 to 0.6486 | 0.625 | 0.4059 to 0.8120 | 1 | 0.4782 to 1.000 | 1.556 | 11.7333 | 0.8879 to 362.8 | 17.95 | 0.8879 to 362.8 | 0.0169 | 0.6786 | 0.1018 |
| CpG 2 | N vs.P | 1 | 0.7820 to 1.000 | 0.05882 | 0.001488 to 0.2869 | 0.4839 | 0.3015 to 0.6694 | 1 | 0.02500 to 1.000 | 1.063 | 2.6667 | 0.1167 to 60.9373 | 2.818 | 0.1065 to 74.57 | 1 | 0.502 | 0.9849 |
|  | P vs.T | 0.9412 | 0.7131 to 0.9985 | 0.3571 | 0.1276 to 0.6486 | 0.64 | 0.4252 to 0.8203 | 0.8333 | 0.3588 to 0.9958 | 1.464 | 3.84 | 0.6262 to 23.55 | 8.889 | 0.8933 to 88.45 | 0.0671 | 0.6597 | 0.1315 |
|  | N vs.T | 1 | 0.7820 to 1.000 | 0.3571 | 0.1276 to 0.6486 | 0.625 | 0.4059 to 0.8120 | 1 | 0.4782 to 1.000 | 1.556 | 11.7333 | 0.7077 to 194.5212 | 17.95 | 0.8879 to 362.8 | 0.0169 | 0.6571 | 0.1499 |
| CpG 3 | N vs.P | 0.7333 | 0.4490 to 0.9221 | 0.4118 | 0.1844 to 0.6708 | 0.5238 | 0.2978 to 0.7429 | 0.6364 | 0.3079 to 0.8907 | 1.247 | 1.44 | 0.5963 to 3.480 | 1.925 | 0.4304 to 8.609 | 0.4719 | 0.5569 | 0.584 |
|  | P vs.T | 0.5882 | 0.3292 to 0.8156 | 0.7857 | 0.4920 to 0.9534 | 0.7692 | 0.4619 to 0.9496 | 0.6111 | 0.3575 to 0.8270 | 2.745 | 1.978 | 1.031 to 3.794 | 5.238 | 1.056 to 25.98 | 0.0669 | 0.7521 | 0.01727 |
|  | N vs.T | 0.7333 | 0.4490 to 0.9221 | 0.7857 | 0.4920 to 0.9534 | 0.7857 | 0.4920 to 0.9534 | 0.7333 | 0.4490 to 0.9221 | 3.422 | 2.946 | 1.219 to 7.124 | 10.08 | 1.815 to 56.02 | 0.0092 | 0.7786 | 0.0107 |
| CpG 4 | N vs.P | 0.6 | 0.3229 to 0.8366 | 0.7059 | 0.4404 to 0.8969 | 0.6429 | 0.3514 to 0.8724 | 0.6667 | 0.4099 to 0.8666 | 2.04 | 1.929 | 0.9008 to 4.129 | 3.6 | 0.8290 to 15.63 | 0.1527 | 0.5588 | 0.5711 |
|  | P vs.T | 0.2941 | 0.1031 to 0.5596 | 0.9286 | 0.6613 to 0.9982 | 0.8333 | 0.3588 to 0.9958 | 0.52 | 0.3131 to 0.7220 | 4.118 | 1.736 | 1.009 to 2.988 | 5.417 | 0.5505 to 53.30 | 0.1848 | 0.8277 | 0.001971 |
|  | N vs.T | 0.6 | 0.3229 to 0.8366 | 0.9286 | 0.6613 to 0.9982 | 0.9 | 0.5550 to 0.9975 | 0.6842 | 0.4345 to 0.8742 | 8.4 | 2.85 | 1.424 to 5.702 | 19.5 | 1.991 to 191.0 | 0.0052 | 0.8071 | 0.004897 |
| CpG 5 | N vs.P | 0.3333 | 0.1182 to 0.6162 | 0.7059 | 0.4404 to 0.8969 | 0.5 | 0.1871 to 0.8129 | 0.5455 | 0.3221 to 0.7561 | 1.133 | 1.1 | 0.5090 to 2.377 | 1.2 | 0.2685 to 5.364 | 1 | 0.5706 | 0.4967 |
|  | P vs.T | 0.2941 | 0.1031 to 0.5596 | 0.9286 | 0.6613 to 0.9982 | 0.8333 | 0.3588 to 0.9958 | 0.52 | 0.3131 to 0.7220 | 4.118 | 1.736 | 1.009 to 2.988 | 5.417 | 0.5505 to 53.30 | 0.1848 | 0.7332 | 0.02764 |
|  | N vs.T | 0.3333 | 0.1182 to 0.6162 | 0.9286 | 0.6613 to 0.9982 | 0.8333 | 0.3588 to 0.9958 | 0.5652 | 0.3449 to 0.7681 | 4.667 | 1.917 | 1.065 to 3.450 | 6.5 | 0.6514 to 64.86 | 0.1686 | 0.8595 | 0.000991 |
| CpG 5 | N vs.P | 1 | 0.7820 to 1.000 | 0.3529 | 0.1421 to 0.6167 | 0.5769 | 0.3692 to 0.7665 | 1 | 0.5407 to 1.000 | 1.545 |  |  | 17.52 | 0.8930 to 343.8 | 0.0192 | 0.5706 | 0.4967 |
|  | P vs.T | 0.6471 | 0.3833 to 0.8579 | 0.7143 | 0.4190 to 0.9161 | 0.7333 | 0.4490 to 0.9221 | 0.625 | 0.3543 to 0.8480 | 2.265 | 1.956 | 0.9687 to 3.948 | 4.583 | 0.9943 to 21.13 | 0.0732 | 0.7332 | 0.02764 |
|  | N vs.T | 1 | 0.7820 to 1.000 | 0.7143 | 0.4190 to 0.9161 | 0.7895 | 0.5443 to 0.9395 | 1 | 0.6915 to 1.000 | 3.5 |  |  | 72.33 | 3.510 to 1491 | < 0.0001 | 0.8595 | 0.000991 |
| CpG 6 | N vs.P | 0.8667 | 0.5954 to 0.9834 | 0.5 | 0.2465 to 0.7535 | 0.619 | 0.3844 to 0.8189 | 0.8 | 0.4439 to 0.9748 | 1.733 | 3.095 | 0.8567 to 11.18 | 6.5 | 1.093 to 38.65 | 0.0538 | 0.7216 | 0.03292 |
|  | P vs.T | 0.5 | 0.2465 to 0.7535 | 0.8571 | 0.5719 to 0.9822 | 0.8 | 0.4439 to 0.9748 | 0.6 | 0.3605 to 0.8088 | 3.5 | 2 | 1.076 to 3.718 | 6 | 1.002 to 35.92 | 0.0577 | 0.7122 | 0.04506 |
|  | N vs.T | 0.8667 | 0.5954 to 0.9834 | 0.8571 | 0.5719 to 0.9822 | 0.8667 | 0.5954 to 0.9834 | 0.8571 | 0.5719 to 0.9822 | 6.067 | 6.067 | 1.656 to 22.23 | 39 | 4.720 to 322.2 | 0.0001 | 0.8714 | 0.000669 |
| CpG 7 | N vs.P | 0.9333 | 0.6805 to 0.9983 | 0.3529 | 0.1421 to 0.6167 | 0.56 | 0.3493 to 0.7560 | 0.8571 | 0.4213 to 0.9964 | 1.442 | 3.92 | 0.6176 to 24.88 | 7.636 | 0.7968 to 73.18 | 0.0881 | 0.6373 | 0.1863 |
|  | P vs.T | 0.6471 | 0.3833 to 0.8579 | 0.5714 | 0.2886 to 0.8234 | 0.6471 | 0.3833 to 0.8579 | 0.5714 | 0.2886 to 0.8234 | 1.51 | 1.51 | 0.7501 to 3.039 | 2.444 | 0.5717 to 10.45 | 0.2895 | 0.7458 | 0.02027 |
|  | N vs.T | 0.9333 | 0.6805 to 0.9983 | 0.5714 | 0.2886 to 0.8234 | 0.7 | 0.4572 to 0.8811 | 0.8889 | 0.5175 to 0.9972 | 2.178 | 6.3 | 0.9705 to 40.90 | 18.67 | 1.892 to 184.1 | 0.0052 | 0.831 | 0.002432 |
| CpG 8 | N vs.P | 1 | 0.7820 to 1.000 | 0.2353 | 0.06811 to 0.4990 | 0.5357 | 0.3387 to 0.7249 | 1 | 0.3976 to 1.000 | 1.308 |  |  | 10.33 | 0.5082 to 210.1 | 0.1041 | 0.5686 | 0.5087 |
|  | P vs.T | 0.7647 | 0.5010 to 0.9319 | 0.7857 | 0.4920 to 0.9534 | 0.8125 | 0.5435 to 0.9595 | 0.7333 | 0.4490 to 0.9221 | 3.569 |  |  | 17.52 | 0.8930 to 343.8 | 0.0192 | 0.8739 | 0.000415 |
|  | N vs.T | 1 | 0.7820 to 1.000 | 0.7857 | 0.4920 to 0.9534 | 0.8333 | 0.5858 to 0.9642 | 1 | 0.7151 to 1.000 | 4.667 |  |  | 101.9 | 4.773 to 2174 | < 0.0001 | 0.9381 | < 0.0001 |
| CpG 9 | N vs.P | 1 | 0.7820 to 1.000 | 0.2353 | 0.06811 to 0.4990 | 0.5357 | 0.3387 to 0.7249 | 1 | 0.3976 to 1.000 | 1.308 |  |  | 10.33 | 0.5082 to 210.1 | 0.1041 | 0.5686 |  |
|  | P vs.T | 0.7647 | 0.5010 to 0.9319 | 0.9286 | 0.6613 to 0.9982 | 0.9286 | 0.6613 to 0.9982 | 0.7647 | 0.5010 to 0.9319 | 10.71 | 3.946 | 1.654 to 9.414 | 42.25 | 4.140 to 431.1 | 0.0002 | 0.9286 | < 0.0001 |
|  | N vs.T | 1 | 0.7820 to 1.000 | 0.9286 | 0.6613 to 0.9982 | 0.9375 | 0.6977 to 0.9984 | 1 | 0.7529 to 1.000 | 14 |  |  | 279 | 10.46 to 7441 | < 0.0001 | 0.9571 | < 0.0001 |
| CpG 10 | N vs.P | 1 | 0.7820 to 1.000 | 0.2353 | 0.06811 to 0.4990 | 0.5357 | 0.3387 to 0.7249 | 1 | 0.3976 to 1.000 | 1.308 |  |  | 10.33 | 0.5082 to 210.1 | 0.1041 | 0.6196 | 0.2495 |
|  | P vs.T | 0.7647 | 0.5010 to 0.9319 | 1 | 0.7684 to 1.000 | 1 | 0.7529 to 1.000 | 0.7778 | 0.5236 to 0.9359 |  | 4.5 | 1.896 to 10.68 | 87 | 4.267 to 1774 | < 0.0001 | 0.9496 | < 0.0001 |
|  | N vs.T | 1 | 0.7820 to 1.000 | 1 | 0.7684 to 1.000 | 1 | 0.7820 to 1.000 | 1 | 0.7684 to 1.000 |  |  |  | 899 | 16.70 to 48390 | < 0.0001 | 1 | < 0.0001 |
| CpG 11 | N vs.P | 0.9333 | 0.6805 to 0.9983 | 0.5294 | 0.2781 to 0.7702 | 0.6364 | 0.4066 to 0.8280 | 0.9 | 0.5550 to 0.9975 | 1.983 | 6.364 | 0.9647 to 41.98 | 15.75 | 1.674 to 148.2 | 0.0073 | 0.7451 | 0.0183 |
|  | P vs.T | 0.4706 | 0.2298 to 0.7219 | 0.9286 | 0.6613 to 0.9982 | 0.8889 | 0.5175 to 0.9972 | 0.5909 | 0.3635 to 0.7929 | 6.588 | 2.173 | 1.250 to 3.777 | 11.56 | 1.222 to 109.2 | 0.0207 | 0.8277 | 0.001971 |
|  | N vs.T | 0.9333 | 0.6805 to 0.9983 | 0.9286 | 0.6613 to 0.9982 | 0.9333 | 0.6805 to 0.9983 | 0.9286 | 0.6613 to 0.9982 | 13.07 | 13.07 | 1.966 to 86.84 | 182 | 10.28 to 3221 | < 0.0001 | 0.9429 | < 0.0001 |
| CpG 12 | N vs.P | 1 | 0.7820 to 1.000 | 0.2353 | 0.06811 to 0.4990 | 0.5357 | 0.3387 to 0.7249 | 1 | 0.3976 to 1.000 | 1.308 |  |  | 10.33 | 0.5082 to 210.1 | 0.1041 | 0.5412 | 0.6917 |
|  | P vs.T | 0.7647 | 0.5010 to 0.9319 | 0.9286 | 0.6613 to 0.9982 | 0.9286 | 0.6613 to 0.9982 | 0.7647 | 0.5010 to 0.9319 | 10.71 | 3.946 | 1.654 to 9.414 | 42.25 | 4.140 to 431.1 | 0.0002 | 0.9349 | < 0.0001 |
|  | N vs.T | 1 | 0.7820 to 1.000 | 0.9286 | 0.6613 to 0.9982 | 0.9375 | 0.6977 to 0.9984 | 1 | 0.7529 to 1.000 | 14 |  |  | 279 | 10.46 to 7441 | < 0.0001 | 0.9524 | < 0.0001 |
| CpG 13 | N vs.P | 0.9333 | 0.6805 to 0.9983 | 0.2353 | 0.06811 to 0.4990 | 0.5185 | 0.3195 to 0.7133 | 0.8 | 0.2836 to 0.9949 | 1.221 | 2.593 | 0.4325 to 15.54 | 4.308 | 0.4241 to 43.76 | 0.3382 | 0.6137 | 0.2735 |
|  | P vs.T | 0.7647 | 0.5010 to 0.9319 | 0.9286 | 0.6613 to 0.9982 | 0.9286 | 0.6613 to 0.9982 | 0.7647 | 0.5010 to 0.9319 | 10.71 | 3.946 | 1.654 to 9.414 | 42.25 | 4.140 to 431.1 | 0.0002 | 0.9139 | < 0.0001 |
|  | N vs.T | 0.9333 | 0.6805 to 0.9983 | 0.9286 | 0.6613 to 0.9982 | 0.9333 | 0.6805 to 0.9983 | 0.9286 | 0.6613 to 0.9982 | 13.07 | 13.07 | 1.966 to 86.84 | 182 | 10.28 to 3221 | < 0.0001 | 0.9548 | < 0.0001 |
| CpG 14 | N vs.P | 1 | 0.7820 to 1.000 | 0.1765 | 0.03799 to 0.4343 | 0.5172 | 0.3253 to 0.7055 | 1 | 0.2924 to 1.000 | 1.214 |  |  | 7.483 | 0.3546 to 157.9 | 0.2288 | 0.5882 | 0.3956 |
|  | P vs.T | 0.8235 | 0.5657 to 0.9620 | 0.9286 | 0.6613 to 0.9982 | 0.9333 | 0.6805 to 0.9983 | 0.8125 | 0.5435 to 0.9595 | 11.53 | 4.978 | 1.779 to 13.93 | 60.67 | 5.579 to 659.7 | < 0.0001 | 0.9412 | < 0.0001 |
|  | N vs.T | 1 | 0.7820 to 1.000 | 0.9286 | 0.6613 to 0.9982 | 0.9375 | 0.6977 to 0.9984 | 1 | 0.7529 to 1.000 | 14 |  |  | 279 | 10.46 to 7441 | < 0.0001 | 0.9643 | < 0.0001 |
| CpG 15 | N vs.P | 1 | 0.7820 to 1.000 | 0.3529 | 0.1421 to 0.6167 | 0.5769 | 0.3692 to 0.7665 | 1 | 0.5407 to 1.000 | 1.545 |  |  | 17.52 | 0.8930 to 343.8 | 0.0192 | 0.6765 | 0.08932 |
|  | P vs.T | 0.6471 | 0.3833 to 0.8579 | 0.9286 | 0.6613 to 0.9982 | 0.9167 | 0.6152 to 0.9979 | 0.6842 | 0.4345 to 0.8742 | 9.059 | 2.903 | 1.465 to 5.751 | 23.83 | 2.475 to 229.5 | 0.0023 | 0.8866 | 0.000263 |
|  | N vs.T | 1 | 0.7820 to 1.000 | 0.9286 | 0.6613 to 0.9982 | 0.9375 | 0.6977 to 0.9984 | 1 | 0.7529 to 1.000 | 14 |  |  | 279 | 10.46 to 7441 | < 0.0001 | 0.9286 | < 0.0001 |
| CpG 16 | N vs.P | 0.9333 | 0.6805 to 0.9983 | 0.3529 | 0.1421 to 0.6167 | 0.56 | 0.3493 to 0.7560 | 0.8571 | 0.4213 to 0.9964 | 1.442 | 3.92 | 0.6176 to 24.88 | 7.636 | 0.7968 to 73.18 | 0.0881 | 0.6549 |  |
|  | P vs.T | 0.6471 | 0.3833 to 0.8579 | 0.9286 | 0.6613 to 0.9982 | 0.9167 | 0.6152 to 0.9979 | 0.6842 | 0.4345 to 0.8742 | 9.059 | 2.903 | 1.465 to 5.751 | 23.83 | 2.475 to 229.5 | 0.0023 | 0.9139 | < 0.0001 |
|  | N vs.T | 0.9333 | 0.6805 to 0.9983 | 0.9286 | 0.6613 to 0.9982 | 0.9333 | 0.6805 to 0.9983 | 0.9286 | 0.6613 to 0.9982 | 13.07 | 13.07 | 1.966 to 86.84 | 182 | 10.28 to 3221 | < 0.0001 | 0.9619 | < 0.0001 |
| CpG 17 | N vs.P | 1 | 0.7820 to 1.000 | 0.3529 | 0.1421 to 0.6167 | 0.5769 | 0.3692 to 0.7665 | 1 | 0.5407 to 1.000 | 1.545 |  |  | 17.52 | 0.8930 to 343.8 | 0.0192 | 0.7137 | 0.03964 |
|  | P vs.T | 0.6471 | 0.3833 to 0.8579 | 0.8571 | 0.5719 to 0.9822 | 0.8462 | 0.5455 to 0.9808 | 0.6667 | 0.4099 to 0.8666 | 4.529 | 2.538 | 1.269 to 5.078 | 11 | 1.822 to 66.40 | 0.0094 | 0.895 | 0.000193 |
|  | N vs.T | 1 | 0.7820 to 1.000 | 0.8571 | 0.5719 to 0.9822 | 0.8824 | 0.6356 to 0.9854 | 1 | 0.7354 to 1.000 | 7 |  |  | 155 | 6.795 to 3536 | < 0.0001 | 0.9571 | < 0.0001 |
| CpG 18 | N vs.P | 1 | 0.7820 to 1.000 | 0.1176 | 0.01458 to 0.3644 | 0.5 | 0.3130 to 0.6870 | 1 | 0.1581 to 1.000 | 1.133 |  |  | 5 | 0.2213 to 113.0 | 0.4859 | 0.6 | 0.3356 |
|  | P vs.T | 0.8824 | 0.6356 to 0.9854 | 0.9286 | 0.6613 to 0.9982 | 0.9375 | 0.6977 to 0.9984 | 0.8667 | 0.5954 to 0.9834 | 12.35 | 7.031 | 1.923 to 25.72 | 97.5 | 7.897 to 1204 | < 0.0001 | 0.937 | < 0.0001 |
|  | N vs.T | 1 | 0.7820 to 1.000 | 0.9286 | 0.6613 to 0.9982 | 0.9375 | 0.6977 to 0.9984 | 1 | 0.7529 to 1.000 | 14 |  |  | 279 | 10.46 to 7441 | < 0.0001 | 0.9571 | < 0.0001 |
| CpG 19 | N vs.P | 0.9333 | 0.6805 to 0.9983 | 0.4118 | 0.1844 to 0.6708 | 0.5833 | 0.3664 to 0.7789 | 0.875 | 0.4735 to 0.9968 | 1.587 | 4.667 | 0.7230 to 30.12 | 9.8 | 1.035 to 92.75 | 0.0411 | 0.6843 | 0.07598 |
|  | P vs.T | 0.5882 | 0.3292 to 0.8156 | 0.9286 | 0.6613 to 0.9982 | 0.9091 | 0.5872 to 0.9977 | 0.65 | 0.4078 to 0.8461 | 8.235 | 2.597 | 1.389 to 4.857 | 18.57 | 1.953 to 176.6 | 0.0036 | 0.8172 | 0.002741 |
|  | N vs.T | 0.9333 | 0.6805 to 0.9983 | 0.9286 | 0.6613 to 0.9982 | 0.9333 | 0.6805 to 0.9983 | 0.9286 | 0.6613 to 0.9982 | 13.07 | 13.07 | 1.966 to 86.84 | 182 | 10.28 to 3221 | < 0.0001 | 0.95 | < 0.0001 |
| CpG 20 | N vs.P | 1 | 0.7820 to 1.000 | 0.05882 | 0.001488 to 0.2869 | 0.4839 | 0.3015 to 0.6694 | 1 | 0.02500 to 1.000 | 1.063 |  |  | 2.818 | 0.1065 to 74.57 | 1 | 0.502 | 0.9849 |
|  | P vs.T | 0.9412 | 0.7131 to 0.9985 | 0.8571 | 0.5719 to 0.9822 | 0.8889 | 0.6529 to 0.9862 | 0.9231 | 0.6397 to 0.9981 | 6.588 | 11.56 | 1.745 to 76.54 | 96 | 7.762 to 1187 | < 0.0001 | 0.9454 | < 0.0001 |
|  | N vs.T | 1 | 0.7820 to 1.000 | 0.8571 | 0.5719 to 0.9822 | 0.8824 | 0.6356 to 0.9854 | 1 | 0.7354 to 1.000 | 7 |  |  | 155 | 6.795 to 3536 | < 0.0001 | 0.9571 | < 0.0001 |
| CpG 21 | N vs.P | 0.8 | 0.5191 to 0.9567 | 0.1765 | 0.03799 to 0.4343 | 0.4615 | 0.2659 to 0.6663 | 0.5 | 0.1181 to 0.8819 | 0.9714 | 0.9231 | 0.3747 to 2.274 | 0.8571 | 0.1450 to 5.067 | 1 | 0.502 | 0.9849 |
|  | P vs.T | 0.8235 | 0.5657 to 0.9620 | 0.7857 | 0.4920 to 0.9534 | 0.8235 | 0.5657 to 0.9620 | 0.7857 | 0.4920 to 0.9534 | 3.843 | 3.843 | 1.376 to 10.73 | 17.11 | 2.871 to 102.0 | 0.0011 | 0.8739 | 0.000415 |
|  | N vs.T | 0.8 | 0.5191 to 0.9567 | 0.7857 | 0.4920 to 0.9534 | 0.8 | 0.5191 to 0.9567 | 0.7857 | 0.4920 to 0.9534 | 3.733 | 3.733 | 1.327 to 10.51 | 14.67 | 2.430 to 88.53 | 0.0028 | 0.9 | 0.000249 |
|  |  |  |  |  |  |  |  |  |  |  |  |  |  |  |  |  |  |
| NPV: Negative predictive value, PPV: Positive predictive value,  CI: Confidence interval, RR: Relative risk, N: Normal, P: Pre-malignant,  T: Tumor | | | | | | | | | |  |  |  |  |  |  |  |  |
