# Supplementary material for: ZNF471 modulates EMT and functions as methylation regulated tumor suppressor with diagnostic and prognostic significance in cervical cancer
Source: Cell Biol Toxicol. 2021 Feb 10;37(5):731–49. doi: 10.1007/s10565-021-09582-4 (PMC8490246; doi:10.1007/s10565-021-09582-4)
Supplement: Supplementary file 20 — (DOCX 36 kb) [file 10565_2021_9582_MOESM14_ESM.docx]

| **Supplementary Table 7: Sensitivity and specificity analysis of public datasets** | | | | | | |  |  |  |  |  |
| --- | --- | --- | --- | --- | --- | --- | --- | --- | --- | --- | --- |
|  |  |  |  |  |  |  |  |  |  |  |  |
|  | **Sensitivity** | **Specificity** | **Positive** | **Negative** | **Disease** | **Positive** | **Negative** | **ROC** | **P value** | **RR** | **P value** |
|  |  |  | **Likelihood** | **Likelihood** | **prevalence** | **Predictive** | **Predictive** |  |  |  |  |
|  |  |  | **Ratio** | **Ratio** |  | **Value** | **Value** |  |  |  |  |
| Total Group |  |  |  |  |  |  |  |  |  |  |  |
| **cg14289985** |  |  |  |  |  |  |  |  |  |  |  |
| **Normal vs. LSIL** | 14.29% (95% CI: 2.37 % to 57.77 %) | 100.00 % (95% CI: 98.08 % to 100.00 %) |  | 0.86 (95% CI: 0.63 to 1.16) | 3.52% (95% CI: 1.43 % to 7.12 %) | 100% (95% CI: 16.55 % to 100.00 %) | 96.97% (95% CI: 93.52 % to 98.87 %) | 0.5322 | 0.7728 | 72.375 | 0.0072 |
| **Normal vs.** | 23.81% (95% CI: 8.31 % to 47.17 %) | 100% (95% CI: 98.08 % to 100.00 %) |  | 0.76 (95% CI: 0.60 to 0.97) | 9.86% (95% CI: 6.21 % to 14.68 %) | 100% (95% CI: 47.95 % to 100.00 %) | 92.31 % (95% CI: 87.81 % to 95.54 %) | 0.5593 | 0.3725 | 96.5 | 0.0017 |
| **HSIL** |  |  |  |  |  |  |  |  |  |  |  |
| **Normal vs. Tumor** | 68.03% (95% CI: 58.98 % to 76.18 %) | 100% (95% CI: 98.08 % to 100.00 %) |  | 0.32 (95% CI: 0.25 to 0.41) | 38.85% (95% CI: 33.43 % to 44.49 %) | 100% (95% CI: 95.61 % to 100.00 %) | 83.12% (95% CI: 77.65 % to 87.71 %) | 0.8395 | < 0.0001 | 262.0407 | 0.0001 |
| **LSIL vs. HSIL** | 23.81% (95% CI: 8.31 % to 47.17 %) | 85.71% (95% CI: 42.23 % to 97.63 %) | 1.67 (95% CI: 0.23 to 11.94) | 0.89 (95% CI: 0.60 to 1.3) | 75% (95% CI: 55.12 % to 89.26 %) | 83.33% (95% CI: 36.10 % to 97.24 %) |  | 0.517 | 0.8945 | 1.6667 | 0.6112 |
| **LSIL vs. Tumor** | 68.03% (95% CI: 58.98 % to 76.18 %) | 85.71% (95% CI: 42.23 % to 97.63 %) | 4.76 (95% CI: 0.77 to 29.35) | 0.37(95% CI: 0.25 to 0.56) | 94.57% (95% CI: 89.13 % to 97.78 %) | 98.81% (95% CI: 93.52 % to 99.80 %) | 13.33% (95% CI: 5.09 % to 26.80 %) | 0.829 | 0.0035 | 4.7623 | 0.0926 |
| **HSIL vs. Tumor** | 68.03% (95% CI: 58.98 % to 76.18 %) | 76.19 % (95% CI: 52.83 % to 91.69 %) | 2.86 (95% CI: 1.32 to 6.20) | 0.42 (95% CI: 0.29 to 0.60) | 85.31 % (95% CI: 78.43 % to 90.67 %) | 94.32% (95% CI: 87.23 % to 98.11 %) | 29.09% (95% CI: 17.64 % to 42.90 %) | 0.8201 | < 0.0001 | 2.8574 | 0.0079 |
| **cg24713204** |  |  |  |  |  |  |  |  |  |  |  |
| **Normal vs. LSIL** | 42.86% (95% CI: 10.42 % to 81.25 %) | 84.29% (95% CI: 78.34 % to 89.14 %) | 2.73 (95% CI: 1.09 to 6.82) | 0.68 (95% CI: 0.36 to 1.29) | 3.54% (95% CI: 1.44 % to 7.15 %) | 9.09% (95% CI: 2.02 % to 24.36 %) | 97.58 % (95% CI: 93.90 % to 99.32 %) | 0.5247 | 0.8246 | 2.7286 | 0.0318 |
| **Normal vs. HSIL** | 80.95% (95% CI: 58.08 % to 94.44 %) | 84.29 % (95% CI: 78.34 % to 89.14 %) | 5.15 (95% CI: 3.49 to 7.60) | 0.23 (95% CI: 0.09 to 0.55) | 9.91% (95% CI: 6.24 % to 14.74 %) | 36.17% (95% CI: 22.68 % to 51.48 %) | 97.58 % (95% CI: 93.90 % to 99.32 %) | 0.91 | < 0.0001 | 5.154 | < 0.0001 |
| **Normal vs Tumor** | 90.16% (95% CI: 83.45 % to 94.81 %) | 84.29 % (95% CI: 78.34 % to 89.14 %) | 5.74 (95% CI: 4.11 to 8.01) | 0.12 (95% CI: 0.07 to 0.20) | 38.98 % (95% CI: 33.54 % to 44.62 %) | 78.57% (95% CI: 70.84 % to 85.05 %) | 93.06% (95% CI: 88.19 % to 96.36 %) | 0.9628 | < 0.0001 | 5.7404 | < 0.0001 |
| **LSIL vs. HSIL** | 80.95 % (95% CI: 58.08 % to 94.44 %) | 57.14% (95% CI: 18.75 % to 89.58 %) | 1.89 (95% CI: 0.78 to 4.55) | 0.33 (95% CI: 0.11 to 0.99) | 75.00 %  (95% CI: 55.12 % to 89.26 %) | 85.00 % (95% CI: 62.08 % to 96.62 %) | 50% (95% CI: 16.01 % to 83.99 %) | 0.7721 | 0.03387 | 1.8889 | 0.1567 |
| **LSIL vs. Tumor** | 90.16% (95% CI: 83.45 % to 94.81 %) | 57.14% (95% CI: 18.75 % to 89.58 %) | 2.10 (95% CI: 0.89 to 4.96) | 0.17 (95% CI: 0.07 to 0.40) | 94.57 % (95% CI: 89.13 % to 97.78 %) | 97.35% (95% CI: 92.43 % to 99.42 %) | 25% (95% CI: 7.42 % to 52.37 %) | 0.8993 | 0.000396 | 2.1038 | 0.0891 |
| **HSIL vs. Tumor** | 90.16% (95% CI: 83.45 % to 94.81 %) | 19.05% (95% CI: 5.56 % to 41.92 %) | 1.11 (95% CI: 0.90 to 1.38) | 0.52 (95% CI: 0.18 to 1.45) | 85.31% (95% CI: 78.43 % to 90.67 %) | 86.61% (95% CI: 79.43 % to 92.00 %) | 25% (95% CI: 7.42 % to 52.37 %) | 0.7395 | 0.000471 | 1.1138 | 0.3272 |
|  |  |  |  |  |  |  |  |  |  |  |  |
| **Individual Studies** |  |  |  |  |  |  |  |  |  |  |  |
| **E-GEOD-30760** |  |  |  |  |  |  |  |  |  |  |  |
|  | Sensitivity | Specificity | Positive | Negative | Disease | Positive | Negative | ROC | P value | Relative risk | P value |
|  |  |  | Likelihood | Likelihood | prevalence | Predictive | Predictive |  |  |  |  |
|  |  |  | Ratio | Ratio |  | Value | Value |  |  |  |  |
| **cg14289985** |  |  |  |  |  |  |  |  |  |  |  |
| **Normal vs Tumor** | 62.9% (95% CI: 49.69 % to 74.83 %) | 95% CI: 97.59 % to 100.00 %) |  | 0.37 (95% CI: 0.27 to 0.51) | 28.84% (95% CI: 22.88 % to 35.39 %) | 95% CI: 90.89 % to 100.00 %) | 86.93% (95% CI: 81.04 % to 91.53 %) | 0.7752 | < 0.0001 | 193.1111 | 0.0002 |
| **cg24713204** |  |  |  |  |  |  |  |  |  |  |  |
| **Normal vs Tumor** | 83.87 (95% CI: 72.33 % to 91.97 %) | 99.35% (95% CI: 96.40 % to 99.89 %) | 128.32 (95% CI: 18.14 to 907.94) | 0.16 (95% CI: 0.09 to 0.29) | 28.84% (95% CI: 22.88 % to 35.39 %) | 98.11% (95% CI: 89.89 % to 99.68 %) | 93.83% (95% CI: 88.94 % to 97.00 %) | 0.9894 | < 0.0001 | 133.2581 | < 0.0001 |
| **E-GEOD-30759** |  |  |  |  |  |  |  |  |  |  |  |
|  | Sensitivity | Specificity | Positive | Negative | Disease | Positive | Negative | ROC | P value | Relative risk | P value |
|  |  |  | Likelihood | Likelihood | prevalence | Predictive | Predictive |  |  |  |  |
|  |  |  | Ratio | Ratio |  | Value | Value |  |  |  |  |
| **cg14289985** |  |  |  |  |  |  |  |  |  |  |  |
| **Normal vs Tumor** | 79.17% (95% CI: 65.00 % to 89.51 %) | 100% (95% CI: 78.03 % to 100.00 %) |  | 0.21 (95% CI: 0.12 to 0.36) | 76.19 % (95% CI: 63.79 % to 86.01 %) | 100% (95% CI: 90.66 % to 100.00 %) | 60% (95% CI: 38.68 % to 78.84 %) | 0.95 | < 0.0001 | 25.1429 | 0.0207 |
| **cg24713204** |  |  |  |  |  |  |  |  |  |  |  |
| **Normal vs Tumor** | 89.58 % (95% CI: 77.33 % to 96.49 %) | 86.67 % (95% CI: 59.51 % to 97.95 %) | 6.72 (95% CI: 1.84 to 24.50) | 0.12 (95% CI: 0.05 to 0.28) | 76.19% (95% CI: 63.79 % to 86.01 %) | 95.56% (95% CI: 84.82 % to 99.33 %) | 72.22% (95% CI: 46.53 % to 90.20 %) | 0.9194 | < 0.0001 | 6.7 | 0.0039 |
|  |  |  |  |  |  |  |  |  |  |  |  |
| **E-GEOD-46306** |  |  |  |  |  |  |  |  |  |  |  |
|  | Sensitivity | Specificity | Positive | Negative | Disease | Positive | Negative | ROC | P value | Relative risk | P value |
|  |  |  | Likelihood | Likelihood | prevalence | Predictive | Predictive |  |  |  |  |
|  |  |  | Ratio | Ratio |  | Value | Value |  |  |  |  |
| **cg14289985** |  |  |  |  |  |  |  |  |  |  |  |
| **Normal vs. HSIL** | 1 (0.7820 to 1.000) | 1 (95% CI: 1.00 to 1.00) | NaN | NaN | 42.86% (95% CI: 26.33 % to 60.64 %) | NaN | 57.14% (95% CI: 39.36 % to 73.67 %) | 0.5533 | 0.5938 | 1.3125 | 0.8903 |
| **HSIL vs. Tumor** | 33.33% (95% CI: 7.88 % to 69.93 %) | 100% (95% CI: 78.03 % to 100.00 %) | NaN | 0.67 (95% CI: 0.42 to 1.06) | 37.5% (95% CI: 18.84 % to 59.40 %) | 100% (95% CI: 30.48 % to 100.00 %) | 71.43% (95% CI: 47.83 % to 88.65 %) | 0.7852 | 0.02173 | 11.2 | 0.0973 |
| **Normal vs. Tumor** | 33.33% (95% CI: 7.88 % to 69.93 %) | 100% (95% CI: 83.01 % to 100.00 %) | NaN | 0.67 (95% CI: 0.42 to 1.06) | 31.03% (95% CI: 15.32 % to 50.83 %) | 100% (95% CI: 30.48 % to 100.00 %) | 76.92 %  (95% CI: 56.35 % to 90.97 %) | 0.8556 | 0.002566 | 14.7 | 0.066 |
| **cg24713204** |  |  |  |  |  |  |  |  |  |  |  |
| **Normal vs. HSIL** | 46.67% (95% CI: 21.34 % to 73.35 %) | 95% (95% CI: 75.05 % to 99.17 %) | 9.33 (95% CI: 1.28 to 67.97) | 0.56 (95% CI: 0.35 to 0.91) | 42.86% (95% CI: 26.33 % to 60.64 %) | 87.5% (95% CI: 47.38 % to 97.93 %) | 70.37 %  (95% CI: 49.82 % to 86.21 %) | 0.52 | 0.8415 | 9.3333 | 0.0275 |
| **HSIL vs. Tumor** | 88.89% (95% CI: 51.74 % to 98.16 %) | 53.33% (95% CI: 26.65 % to 78.66 %) | 1.9 (95% CI: 1.06 to 3.43) | 0.21 (95% CI: 0.03 to 1.40) | 37.5% (95% CI: 18.84 % to 59.40 %) | 53.33% (95% CI: 26.65 % to 78.66 %) | 88.89% (95% CI: 51.74 % to 98.16 %) | 0.7926 | 0.01854 | 1.9048 | 0.0318 |
| **Normal vs. Tumor** | 88.89% (95% CI: 51.74 % to 98.16 %) | 95% (95% CI: 75.05 % to 99.17 %) | 17.78 (95% CI: 2.60 to 121.78) | 0.12 (95% CI: 0.02 to 0.74) | 31.03% (95% CI: 15.32 % to 50.83 %) | 88.89 % (95% CI: 51.74 % to 98.16 %) | 95% (95% CI: 75.05 % to 99.17 %) | 0.7889 | 0.01427 | 17.7778 | 0.0034 |
|  |  |  |  |  |  |  |  |  |  |  |  |
| **E-GEOD-41384** |  |  |  |  |  |  |  |  |  |  |  |
|  | Sensitivity | Specificity | Positive | Negative | Disease | Positive | Negative | ROC | P value | Relative risk | P value |
|  |  |  | Likelihood | Likelihood | prevalence | Predictive | Predictive |  |  |  |  |
|  |  |  | Ratio | Ratio |  | Value | Value |  |  |  |  |
| **cg14289985** |  |  |  |  |  |  |  |  |  |  |  |
| **Normal vs. LSIL** | 14.29% (95% CI: 2.37 % to 57.77 %) | 100% (95% CI: 30.48 % to 100.00 %) | NaN | 0.86 (95% CI: 0.63 to 1.16) | 70.00 % (95% CI: 34.84 % to 92.97 %) | 100.00 % (95% CI: 16.55 % to 100.00 %) | 33.33 %  (95% CI: 7.88 % to 69.93 %) | 0.7143 | 0.3051 | 2.5 | 0.5196 |
| **Normal vs. HSIL** | 83.33%(95% CI: 36.10 % to 97.24 %) | 100% (95% CI: 30.48 % to 100.00 %) | NaN | 0.17 (95% CI: 0.03 to 1.00) | 66.67 % (95% CI: 30.07 % to 92.12 %) | 100% (95% CI: 47.95 % to 100.00 %) | 75% (95% CI: 20.34 % to 95.88 %) | 1 | 0.02018 | 6.2857 | 0.1693 |
| **Normal vs. Tumor** | 100% (95% CI: 30.48 % to 100.00 %) | 100% (95% CI: 30.48 % to 100.00 %) | NaN | 0 | 50% (95% CI: 12.42 % to 87.58 %) | 100% (95% CI: 30.48 % to 100.00 %) | 100% (95% CI: 30.48 % to 100.00 %) | 1 | 0.04959 | 7 | 0.1453 |
| **LSIL vs. HSIL** | 83.33% (95% CI: 36.10 % to 97.24 %) | 85.71% (95% CI: 42.23 % to 97.63 %) | 5.83 (95% CI: 0.92 to 37.08) | 0.19 (95% CI: 0.03 to 1.19) | 46.15 % (95% CI: 19.33 % to 74.78 %) | 83.33% (95% CI: 36.10 % to 97.24 %) | 85.71 % (95% CI: 42.23 % to 97.63 %) | 0.9762 | 0.004292 | 5.8333 | 0.0616 |
| **LSIL vs. Tumor** | 100% (95% CI: 30.48 % to 100.00 %) | 85.71% (95% CI: 42.23 % to 97.63 %) | 7 (95% CI: 1.14 to 42.97) | 0 | 30% (95% CI: 7.03 % to 65.16 %) | 75% (95% CI: 20.34 % to 95.88 %) | 100% (95% CI: 54.05 % to 100.00 %) | 1 | 0.01674 | 7 | 0.0356 |
| **HSIL vs. Tumor** | 100% (95% CI: 30.48 % to 100.00 %) | 16.67% (95% CI: 2.76 % to 63.90 %) | 1.2 (95% CI: 0.84 to 1.72) | 0 | 33.33% (95% CI: 7.88 % to 69.93 %) | 37.50 % (95% CI: 8.97 % to 75.30 %) | 100.00 % (95% CI: 16.55 % to 100.00 %) | 0.8889 | 0.07076 | 1.2 | 0.3180 |
| **cg24713204** |  |  |  |  |  |  |  |  |  |  |  |
| **Normal vs. LSIL** | 28.57% (95% CI: 4.52 % to 70.73 %) | 100% (95% CI: 30.48 % to 100.00 %) | NaN | 0.71 (95% CI: 0.45 to 1.14) | 70%(95% CI: 34.84 % to 92.97 %) | 100% (95% CI: 19.29 % to 100.00 %) | 37.5% (95% CI: 8.97 % to 75.30 %) | 0.7143 | 0.3051 | 2.5 | 0.5196 |
| **Normal vs. HSIL** | 83.33% (95% CI: 36.10 % to 97.24 %) | 100.00 % (95% CI: 30.48 % to 100.00 %) | NaN | 0.17 (95% CI: 0.03 to 1.00) | 66.67% (95% CI: 30.07 % to 92.12 %) | 100% (95% CI: 47.95 % to 100.00 %) | 75% (95% CI: 20.34 % to 95.88 %) | 0.8889 | 0.07076 | 6.285 | 0.1693 |
| **Normal vs. Tumor** | 50.00 % (95% CI: 8.30 % to 91.70 %) | 100% (95% CI: 30.48 % to 100.00 %) | NaN | 0.50 (95% CI: 0.19 to 1.33) | 57.14% (95% CI: 18.75 % to 89.58 %) | 100.00 % (95% CI: 19.29 % to 100.00 %) | 60% (95% CI: 15.40 % to 93.51 %) | 0.6667 | 0.5127 | 4 | 0.3208 |
| **LSIL vs. HSIL** | 83.33% (95% CI: 36.10 % to 97.24 %) | 71.43% (95% CI: 29.27 % to 95.48 %) | 2.92 (95% CI: 0.86 to 9.93) | 0.23 (95% CI: 0.04 to 1.48) | 46.15% (95% CI: 19.33 % to 74.78 %) | 71.43% (95% CI: 29.27 % to 95.48 %) | 83.33 %  (95% CI: 36.10 % to 97.24 %) | 0.9524 | 0.006665 | 2.9 | 0.0867 |
| **LSIL vs. Tumor** | 50% (95% CI: 8.30 % to 91.70 %) | 71.43% (95% CI: 29.27 % to 95.48 %) | 1.75 (95% CI: 0.38 to 8.06) | 0.7 (95% CI: 0.24 to 2.07) | 36.36% (95% CI: 11.15 % to 69.12 %) | 50% (95% CI: 8.30 % to 91.70 %) | 71.43% (95% CI: 29.27 % to 95.48 %) | 0.8571 | 0.08744 | 1.75 | 0.4726 |
| **HSIL vs. Tumor** | 50% (95% CI: 8.30 % to 91.70 %) | 16.67% (95% CI: 2.76 % to 63.90 %) | 0.6 (95% CI: 0.21 to 1.70) | 3 (95% CI: 0.39 to 23.07) | 40% (95% CI: 12.40 % to 73.63 %) | 28.57% (95% CI: 4.52 % to 70.73 %) | 33.33 %  (95% CI: 5.47 % to 88.45 %) | 0.5 | 1 | 0.6 | 0.3372 |
| LSIL: Low grade squamous intra epithelial lesion, HSIL: High grade squamous intra epithelial lesion, NaN: Not applicable | | | | | | | | | |  |  |
