# Supplementary material for: ZNF471 modulates EMT and functions as methylation regulated tumor suppressor with diagnostic and prognostic significance in cervical cancer
Source: Cell Biol Toxicol. 2021 Feb 10;37(5):731–49. doi: 10.1007/s10565-021-09582-4 (PMC8490246; doi:10.1007/s10565-021-09582-4)
Supplement: Supplementary file 21 — (DOCX 15 kb) [file 10565_2021_9582_MOESM15_ESM.docx]

**Supplementary Table 8: Pearson correlation analysis between *ZNF471* methylation with EMT marker expression**

|  | ***ZNF  vs. CDH1*** | ***ZNF  vs. CDH2*** | ***ZNF  vs. VIM*** | ***ZNF  vs. ZEB1*** | ***ZNF  vs. TWIST1*** | ***ZNF  vs. SNAI1*** | ***ZNF  vs. SNAI2*** | ***ZNF  vs. CTNNB1*** |
| --- | --- | --- | --- | --- | --- | --- | --- | --- |
| **Pearson r** | 0.1424 | -0.2497 | -0.3382 | -0.2425 | -0.2649 | -0.2036 | -0.09053 | -0.2667 |
| **95% confidence interval** | 0.03132 to 0.2500 | -0.3518 to -0.1417 | -0.4337 to -0.2351 | -0.3450 to -0.1342 | -0.3659 to -0.1576 | -0.3085 to -0.09377 | -0.2004 to 0.02162 | -0.3677 to -0.1595 |
| **R squared** | 0.02028 | 0.06236 | 0.1143 | 0.05879 | 0.07015 | 0.04144 | 0.008197 | 0.07113 |
| **P (two-tailed)** | 0.0122 | <0.0001 | <0.0001 | <0.0001 | <0.0001 | 0.0003 | 0.1134 | <0.0001 |
