# Supplementary material for: ZNF471 modulates EMT and functions as methylation regulated tumor suppressor with diagnostic and prognostic significance in cervical cancer
Source: Cell Biol Toxicol. 2021 Feb 10;37(5):731–49. doi: 10.1007/s10565-021-09582-4 (PMC8490246; doi:10.1007/s10565-021-09582-4)
Supplement: Supplementary file 22 — (DOCX 15 kb) [file 10565_2021_9582_MOESM16_ESM.docx]

| **CpG sites** | **Highly correlated CpG sites** |
| --- | --- |
| BRCA | cg19358877 |
| COAD | cg14042851 |
| ESCA | cg19358877 |
| GBM | cg19811761 |
| HNSC | cg14042851 |
| KIRC | cg19358877 |
| KIRP | cg14277392 |
| LUAD | cg00674365 |
| LUSC | cg11539780 |
| PAAD | cg19358877 |
| READ | cg14289985 |
| SARC | cg14277392 |
| STAD | cg11539780 |
| THCA | cg14289985 |
| UCEC | cg00674365 |

**Supplementary Table 9:** **The highly correlated co-methylated CpG sites with potential to distinguish between cancers**
